# PrimeStack editors enable efficient and site-specific integration of large multigene cargos in plants

**DOI:** 10.1371/journal.pbio.3003778

**Published:** 2026-10-13

**Authors:** Edith Sánchez, Khalid Sedeek, Haroon Butt, Magdy Mahfouz

**Affiliations:** Laboratory for Genome Engineering and Synthetic Biology, Division of Biomedical Sciences, King Abdullah University of Science and Technology (KAUST), Thuwal, Saudi Arabia; ETH Zurich, SWITZERLAND

## Abstract

Precise, site-specific integration of large DNA sequences into plant genomes is a long-standing goal of crop biotechnology and synthetic biology, yet remains constrained by random insertion, inefficient homologous recombination, and gene targeting. Here, we present PrimeStack, a DSB-independent platform that integrates PE with the unidirectional large serine integrase Bxb1, leveraging evolved variants for enhanced activity, to achieve the programmable insertion of multigene, multikilobase cargos at predefined genomic safe harbors (GSH) in rice (*Oryza sativa*). Optimized prime editors first install *attP* landing sites with high fidelity and heritability followed by Bxb1-mediated recombination that generates irreversible integration of genetic information. PrimeStack enabled targeted large-cargo integration in regenerated rice plants with efficiencies of up to 45%, as determined by junction-specific PCR analyses. No obvious morphological differences from WT plants were observed at 5 weeks after transfer to soil under the evaluated conditions. We validate its utility by achieving targeted insertion of a carotenoid cassette at an experimentally confirmed genomic safe harbor. PrimeStack delivers a modular, site-specific gene stacking platform that enables predictable, multigene trait pyramiding and pathway construction at GSH, thereby accelerating the development of improved and resilient crop varieties, as well as scalable plant-based biomanufacturing and a powerful chassis for synthetic biology.

## Abstract 2

Precise integration of large DNA sequences into plant genomes remains a major challenge. This study presents PrimeStack, a platform that combines prime editing with the Bxb1 integrase to achieve the programmable insertion of multigene cargos at plant genomic safe harbors with high efficiency.

## Introduction

A central challenge in plant biotechnology and synthetic biology is achieving highly efficient, precise, and site-specific integration of multikilobase genetic information into genomic safe harbors (GSH), which could unlock transformative advances in crop improvement and green biomanufacturing [1–4]. Targeted addition of single or multigene traits into predefined, neutral loci promises predictable expression, stable inheritance, and modular engineering, enabling enhanced yield, climate resilience, nutritional biofortification, and the sustainable production of high-value therapeutics, biomaterials, and industrially relevant compounds in plant chassis [5–8]. However, despite decades of progress, achieving high-efficiency, universal, and precise large DNA insertion remains a central bottleneck, limiting the rational redesign of complex traits and the full exploitation of plants as programmable biofactories [9–12].

Transgenesis, pioneered through *Agrobacterium*-mediated T-DNA transfer and biolistic delivery, has revolutionized crop biotechnology by introducing traits of value such as herbicide tolerance, pest resistance, and nutritional enhancements [13–16]. However, the inherent random integration of genetic information frequently results in positional variegation, epigenetic silencing, gene disruption, multiple copy insertions, and reliance on selectable markers, which have contributed to regulatory hurdles and public concerns about transgenic crops, including uncertainty over insertional effects, reliance on antibiotic resistance markers, and the unpredictable nature of random integration events, among multiple other societal and institutional factors [17–22]. Cisgenic and intragenic strategies mitigated some concerns by using compatible genetic elements, but failed to resolve unpredictability and expression instability [23–26].

The advent of sequence-specific nucleases such as meganucleases, zinc-finger nucleases, TALENs, and CRISPR–Cas9 has enabled programmable generation of site-specific double-strand breaks (DSBs), shifting the paradigm toward precision editing [27–33]. CRISPR–Cas systems are highly programmable and efficient for the generation of site-specific DSBs across diverse species, including plants. While DSBs excel at generating knockouts via nonhomologous end joining, homology-directed repair for targeted insertions of genetic cargo is notoriously inefficient in plant somatic cells, rendering multikilobase integrations impractical [34–38]. Prime editing (PE), a DSB-independent technology that precisely rewrites genetic information via pegRNAs, has expanded capabilities for base substitutions, indels, and small gene sequence addition in plants [39–43]. However, its cargo size limitation (up to 200 bp) precludes the insertion of full genes or pathways essential for most agronomic and synthetic biology applications [44–46].

Site-specific recombinases (SSRs) offer an alternative for large DNA integration. Tyrosine recombinases (e.g., Cre-lox, FLP-FRT) and serine integrases (e.g., PhiC31, Bxb1) have long been used in plants for marker excision and limited cassette exchange [47–52]. Their utility for de novo insertions, however, requires pre-installation of recognition sites, a challenge recently addressed by coupling PE with SSRs. PrimeRoot, for instance, employs PE to insert lox sites followed by Cre-mediated integration of up to 11.1-kb cargos in rice, achieving 6%–8% efficiency and marker-free trait delivery [13]. However, Cre’s bidirectional nature, even with pseudo-unidirectional mutants, risks insert instability across generations, while shorter recognition sites raise specificity concerns in repetitive plant genomes [53–55].

Large serine integrases such as Bxb1 provide compelling advantages: strict unidirectionality yielding irreversible *attL*/*attR* hybrids, longer asymmetric sites (38–50 bp) for enhanced fidelity, and established robustness for gene stacking in plants [9,10,56–59]. In mammalian cells, prime-editing-assisted site-specific integrase gene editing (PASSIGE) and its evolved eePASSIGE variant harness phage-assisted evolution of Bxb1 to achieve >30% integration of >10-kb sequences, outperforming fusion-based approaches and demonstrating therapeutic potential [12,14,60]. Recent megabase-scale chromosome engineering further underscores the power of optimized recombinases combined with scar-free strategies [61].

Here, we introduce PrimeStack, a DSB-free platform that integrates PE with Bxb1, including evolved variants, for programmable, irreversible insertion of multigene cassettes into experimentally validated GSH in rice. By generating recombination-ready germplasm with precisely installed *attP* sites at neutral loci, PrimeStack enables high-efficiency docking of large genetic payloads, resulting in phenotypically neutral lines with stable cassette insertion. This technology overcomes the reversibility and efficiency constraints of Cre-based systems, establishes a universal pipeline for large-scale DNA integration across crops, and paves the way for predictive trait pyramiding, complex pathway assembly, and the realization of plants as scalable, sustainable biofactories [61–64].

## Results

### Selection of genomic safe-harbor loci and prime-editing strategy for Bxb1 *attP* landing pad installation

Although locus-directed integration has been reported in rice [13], experimentally validated GSH sites remain limited, and the feasibility of installing standardized recombinase landing pads at such loci has not been systematically assessed. We therefore sought to empirically evaluate candidate GSH loci for their ability to tolerate precise installation of a Bxb1 *attP* landing site without detectable fitness penalties, thereby establishing a platform for modular, site-specific transgene integration. We selected eight candidate loci for experimental validation in *Oryza sativa* ssp. *japonica* cv. Nipponbare: six loci nominated from prior computational GSH predictions [13] and two loci within the 25S rDNA region, which has been proposed as a permissive genomic compartment for transgene insertion (Fig 1A) [65]. Non-rDNA candidates were defined using stringent exclusion criteria, prioritizing intergenic regions separated from annotated regulatory and noncoding features (≥5 kb from protein-coding genes and predicted promoters/enhancers, ≥30 kb from microRNA loci, ≥20 kb from lncRNA and tRNA genes, and ≥10 kb from centromeric regions). The final panel comprised two targets within a genomic safe harbor on chromosome 1, previously identified as conserved across 33 analyzed rice genome assemblies [13], one target each on chromosomes 3, 6, 7, and 11, and two targets within the 25S rDNA region on chromosome 9 (Fig 1B). To benchmark landing-pad installation in a potentially less “neutral” context, we additionally included two loci proximal to coding genes, near the promoter regions of *OsMOC1* and *OsTT1* [66,67]. The precise genomic coordinates of each installed *attP* site are provided in S1 Table.

**Fig 1:**
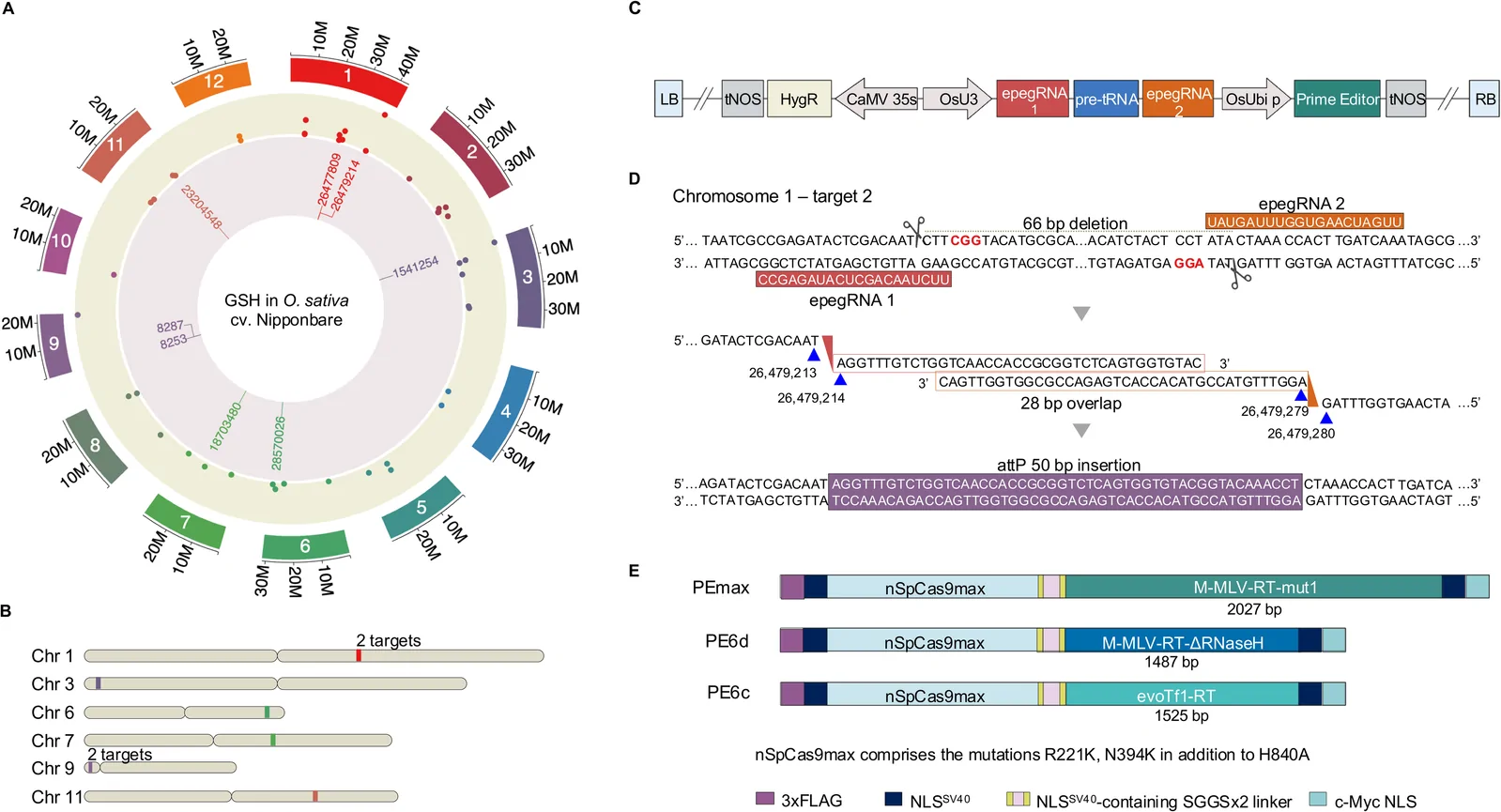
Selection of genomic safe harbors (GSH) and reagents for installation of Bxb1 landing pads (*attP*) in rice genome. **(A)** Circular ideogram of *O. sativa* ssp. *japonica* cv. Nipponbare genome, generated with Circa 2.0 (OMGenomics Labs, https://circa.omgenomics.com), depicting its relative chromosome sizes (outer ring), the genome-wide distribution of the 44 computationally predicted GSH loci (middle ring), and the genomic positions of the engineered Bxb1 *attP* landing pads introduced at selected GSH (inner ring). Numbers in the inner ring indicate the genomic coordinate of the first nucleotide of each inserted *attP* site. **(B)** Chromosomal distribution of GSH loci across individual rice chromosomes. **(C)** Structure of the *Agrobacterium*-transformation cassette encoding prime-editing components for *attP* installation. **(D)** Representative target site Chr 1-2 illustrating Twin-PE-mediated insertion of the Bxb1 *attP* site. Selected PAM sequences are highlighted in red, and blue arrows indicate the position and orientation of the *attP* insertion relative to the native genome sequence. **(E)** Overview of the prime editor architectures evaluated in this study. Created in BioRender. Sanchez Hernandez, E. (2026) https://BioRender.com/uwdtl51.

To install Bxb1 *attP* landing sites, we implemented a dual-pegRNA (Twin-PE) strategy that enables programmable insertion of defined sequences and has been shown to be effective in human cells [7,12] and plants [13,46]. For each targeted locus, Twin-PE constructs were delivered into rice callus via *Agrobacterium*-mediated transformation. The T-DNA was designed to express two epegRNAs under the OsU3 promoter and a prime editor under the rice ubiquitin promoter. A hygromycin selection cassette was also used to enable selection of the transformants (Fig 1C). Details about the structure and sequences of the dual-epegRNA cassettes can be found in S4 Fig and S6 and S3 Tables.

Twin-PE utilizes two pegRNAs positioned on opposite DNA strands, each with a reverse-transcription (RT) template that is complementary to the other. The RT templates share a 28-bp overlap, which supports the productive joining of the edited intermediates and the insertion of the full 50-bp *attP* sequence (Fig 1D) [68]. To stabilize pegRNAs and increase editing performance, we appended the tevopreQ1 stabilizing motif to the 3′ end of each pegRNA [68] across the 10 target sites, generating engineered pegRNAs (epegRNAs). This architecture enabled the installation of an identical 50-bp *attP* element at each selected locus [7,12].

Because PE performance can be strongly locus- and editor-dependent, we compared three prime editor variants, PEmax, PE6c, and PE6d, which were previously reported to exhibit target-dependent differences in mammalian systems and plants. These editors primarily differ in their reverse transcriptase (RT) modules and overall size. PEmax contains the SpCas9 (R221K, N394K, and H840A) nickase fused to the M-MLV-RT-mut1, a human codon-optimized reverse transcriptase of 2,027 bp. In contrast, the PE6d variant incorporates the mutations T128N, V223Y, and D200C within the RT domain and lacks the RNase H module, resulting in a substantially smaller protein with a coding sequence of 1,487 bp. Finally, PE6c carries an evolved Tf1 reverse transcriptase of approximately 1,525 bp [69–72]. These architectural differences provided an opportunity to evaluate how RT origin, size, and composition influence Twin-PE performance across target loci (Fig 1E [73]).

### Precise and heritable installation of *attP* landing pads

Regenerated T_0_ plants were screened by PCR to identify *attP* insertion events. Among the 10 targets evaluated, correct *attP* installation was detected at four genomic safe-harbor loci located in Chr 1-2, Chr 3, Chr 7, and Chr 11, as well as at one site proximal to the promoter region of *OsMOC1*. Figure 2A shows representative events containing the edited amplicons at the engineered GSHs in comparison with the WT counterparts, with expected edited bands indicated by red arrows. Single bands at the expected edited sizes for the Chr 1-2 and Chr11 (263 bp and 194 bp, respectively) evidenced homozygous insertion events. The edited event at Chr 3 displayed two bands, one corresponding to the expected edited amplicon (291 bp) and an additional band of ~250 bp that does not correspond to the WT allele, whose expected size is 340 bp. For the selected event at the Chr 7 GSH, three bands were observed, suggesting chimerism.

**Fig 2:**
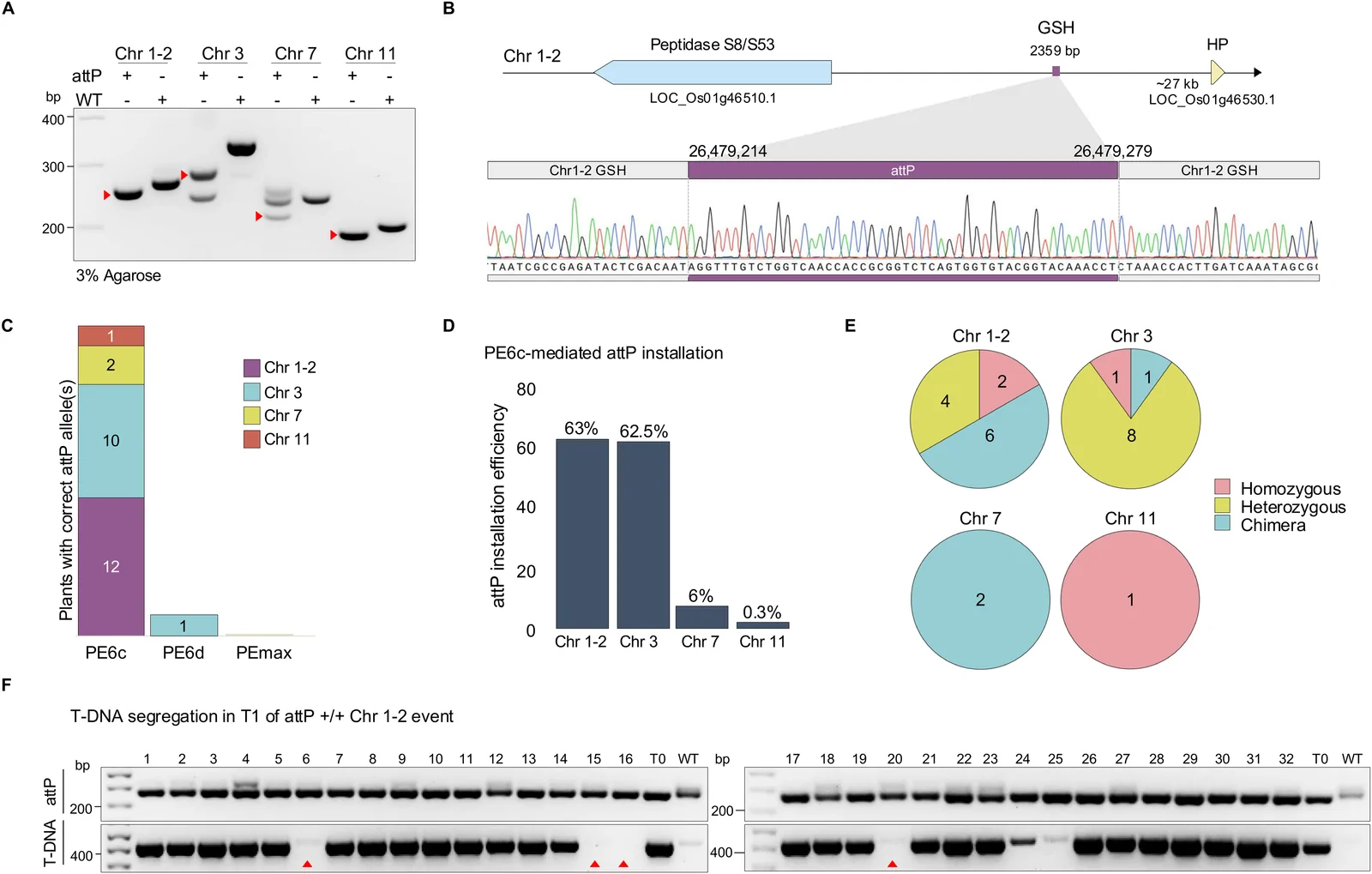
Precise integration and germline transmission of Bxb1 *attP* landing pads. **(A)** 3% gel electrophoresis confirming successful installation of the *attP* landing pad at selected genomic safe harbor (GSH) loci. Arrowheads indicate the expected amplicons corresponding to correct on-target insertions. **(B)** Genomic context of the representative *attP* insertion at the chromosome 1-2 GSH, with a Sanger sequencing chromatogram confirming precise on-target integration. **(C)** Number of regenerated rice plants harboring correctly installed *attP* alleles generated using three prime editor variants (PE6c, PE6d, and PEmax). PE6c produced the highest number of correctly edited plants across multiple loci. **(D)** Editing efficiency of PE6c-mediated *attP* allele installation across the targeted GSH loci in rice. **(E)** Zygosity distribution of regenerated plants carrying *attP* insertions at each target site. **(F)** T-DNA segregation analysis in T1 progeny derived from a homozygous *attP* insertion event at the chromosome 1-2 GSH locus, demonstrating stable *attP* germline transmission and transgene segregation. The underlying numerical data for panels C–E are provided in S1 Data.

To evaluate the fidelity of *attP* landing-pad installation, amplicons from PCR positive T_0_ plants were subjected to Sanger sequencing. Sequence analysis confirmed the presence of accurate and complete *attP* insertions in the selected events. Figure 2B shows a representative chromatogram demonstrating precise installation of the *attP* site at Chr 1-2.

During the initial screening, five regenerated plants were analyzed for each prime editor at each of the five target sites. PE6c yielded regenerated plants carrying precisely installed *attP* alleles at multiple loci, whereas PE6d yielded one regenerated plant with a precisely installed *attP* allele, and no precise *attP* installations were detected among the regenerated plants analyzed using PEmax under our experimental conditions (Fig 2C and S7 Table). Given the limited number of regenerated plants evaluated per editor at each target site, these observations do not support a statistically robust comparison of the relative editing efficiencies of the three PE systems. Based on the observed outcomes, PE6c was selected for subsequent experiments because it reproducibly supported precise *attP* installation across multiple loci. Additional regenerated plants were subsequently screened using PE6c, yielding a total of 25 plants carrying correctly installed *attP* alleles across four GSH loci: 12 at Chr 1-2, 10 at Chr 3, two at Chr 7, and one at Chr 11 (Fig 2C and S7 and S9 Tables).

Using PE6c, at least one correctly inserted *attP* allele was detected in 63% of regenerated plants at the GSH in chromosome 1-target 2 and 62.5% at the chromosome 3 GSH, whereas markedly lower efficiencies were observed at the chromosome 7 GSH (6%) and chromosome 11 GSH (0.3%) (Fig 2D). It should be noted that these percentages represent the proportion of regenerated plants in which any *attP*-containing allele was detectable by PCR and Sanger sequencing, and are therefore qualitative in nature. At the *OsMOC1* locus, one of eight regenerated plants contained a sequence-confirmed precise *attP* insertion, corresponding to an installation frequency of 12.5%; however, the zygosity of this event could not be conclusively determined (S5 Fig). All recovered plants were morphologically normal and produced viable seeds, consistent with phenotypic neutrality of landing-pad installation at these sites under our growth conditions. The total number of independent lines is summarized in S7 Table, including the numbers of lines carrying precise insertions classified as homozygous, heterozygous, chimeric, or of undetermined zygosity, as well as the numbers of nonprecisely edited and wild-type (WT) lines.

Consistent with its higher installation frequency, the chromosome 1-target 2 GSH yielded two independent T_0_ events that were homozygous for a sequence-accurate *attP* insertion. One of these events was selected for segregation of the T-DNA in the T_1_ generation. T_1_ plants were analyzed via PCR and Sanger sequencing to confirm *attP* presence and sequence integrity, while PCR screening for T-DNA enabled identification of T-DNA-free individuals (Figs 2F and S9). Plants lacking T-DNA were advanced to maturity, and T_2_ seeds were collected for downstream applications.

### Bxb1-mediated site-specific integration of minicircle donor transgenes in *attP*-engineered rice calli

Minicircle DNA vectors are minimal, backbone-free circular DNA molecules that are widely used for gene delivery and transgene expression in mammalian systems [12]; however, their application in plant genome engineering remains limited. We therefore explored whether minicircle donors could serve as “clean” payloads for site-specific, recombinase-mediated integration in plants when coupled to Bxb1. To our knowledge, this provides an early demonstration of Bxb1-mediated integration of minicircle donors into engineered *attP* landing pads in the plant genome, offering a potentially streamlined route to precise transgene insertion with minimal vector-derived sequence.

The carotenoid cassette comprises two expression units, each driven by the endosperm-specific glutelin promoter. The first encodes SSU-*crtI*, consisting of the bacterial *Erwinia uredovora crtI* carotenoid desaturase fused to the chloroplast transit peptide from the pea RUBISCO small subunit (SSU), enabling plastid targeting where carotenoid biosynthesis occurs. The second unit encodes *ZmPsy*, the maize phytoene synthase that catalyzes the first step of the pathway by converting geranylgeranyl diphosphate (GGPP) into phytoene (Fig 3A).

**Fig 3:**
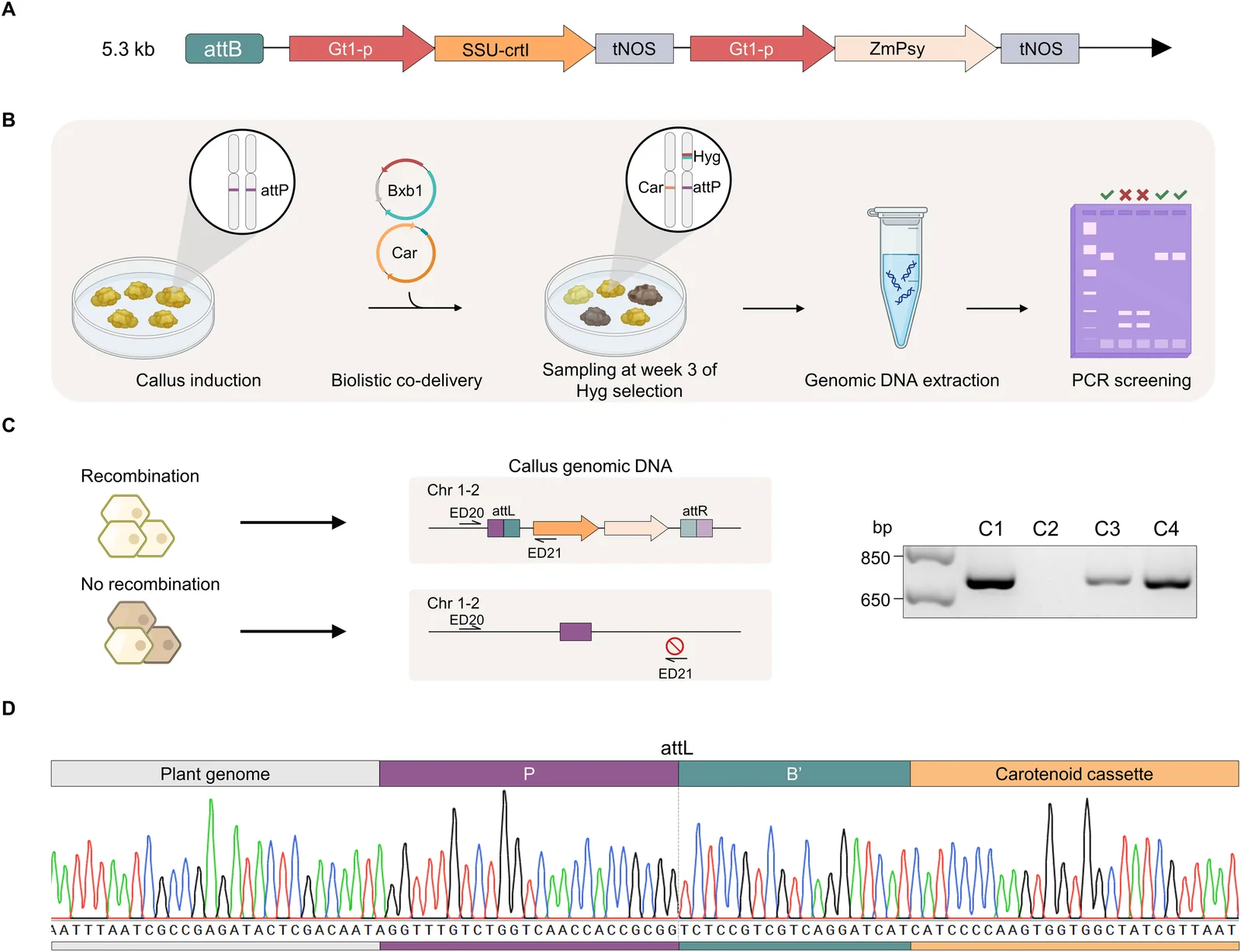
Biolistic co-delivery of a carotenoid minicircle donor and Bxb1 recombinase into *attP*-engineered callus. **(A)** Schematic of the carotenoid biosynthesis cassette highlighting the *attB* site and the metabolic pathway components inserted in this study. **(B)** Transformation workflow: calli were induced from seeds harboring *attP* sites at defined genomic loci. A plant expression vector encoding wild-type (WT) Bxb1 recombinase and a hygromycin resistance marker, together with the minicircle carotenoid donor carrying *attB* sites, were introduced into engineered calli by particle bombardment. **(C)** Identification of precise insertion events by junction PCR. **(D)** Representative Sanger sequencing chromatogram of PCR-positive callus samples confirming correct formation of the *attL* junction at the targeted integration site. Created in BioRender. Sanchez hernandez, E. (2026) https://BioRender.com/xi0j927.

To test the Bxb1-mediated insertion of the minicircle donor, callus derived from the T-DNA-free homozygous line containing *attP* at the GSH Chr 1-2 was subjected to particle bombardment with the purified minicircle donor carrying the Bxb1 *attB* site and the carotenoid biosynthesis cassette (5.3 kb), together with a plasmid expressing the Bxb1 recombinase (Fig 3B [74]). Junction-specific products were detected, demonstrating successful Bxb1-mediated integration of the donor cassette at the intended genomic landing pad (Fig 3C [74]). Sanger sequencing of representative left-junction amplicons verified the presence of the expected *attL* junction and showed that the flanking genomic and donor sequences were accurately preserved without detectable perturbations (Fig 3D).

### Targeted integration of a larger dual cassette at a single genomic locus

Having established site-specific integration of a 5.3-kb metabolic cassette, we next challenged our platform with a larger, dual-function payload. We assembled a 9.4-kb cassette combining the carotenoid biosynthesis pathway with a mutated version of the acetolactate synthase gene (*OsALS* W548L), which confers resistance to ALS-inhibiting herbicides such as bispyribac sodium (Fig 4A). T-DNA-free callus derived from the rice line containing *attP* landing pad lines on GSH Chr 1-2 was bombarded with the 9.4-kb minicircle donor together with each of the three Bxb1 recombinase variants. After three weeks of hygromycin selection, site-specific integration was assessed by junction PCR. Left junction integration was detected by amplification of an 810-bp product across multiple callus mixtures for all three Bxb1 variants, and right junction amplification yielded products of the expected 502 bp (Fig 4B–4D). Sanger sequencing confirmed accurate left and right recombination junctions (Fig 4E, 4F), with targeted integration detected in 40–50% of the pooled callus samples analyzed (S8 Table). Each pooled sample consisted of 10 callus pieces, and 10 pooled samples were analyzed for each recombinase variant. Given the limited number of pooled samples, these data are presented as a proof-of-concept demonstration of 9.4-kb targeted integration rather than as a statistically robust comparison of recombination efficiencies among the three Bxb1 variants. Although regeneration of plants carrying the dual-trait donor was not achieved under the tested selection conditions, the robust molecular validation at the callus stage supports site-specific integration of the dual-cassette cargo at a single genomic locus.

**Fig 4:**
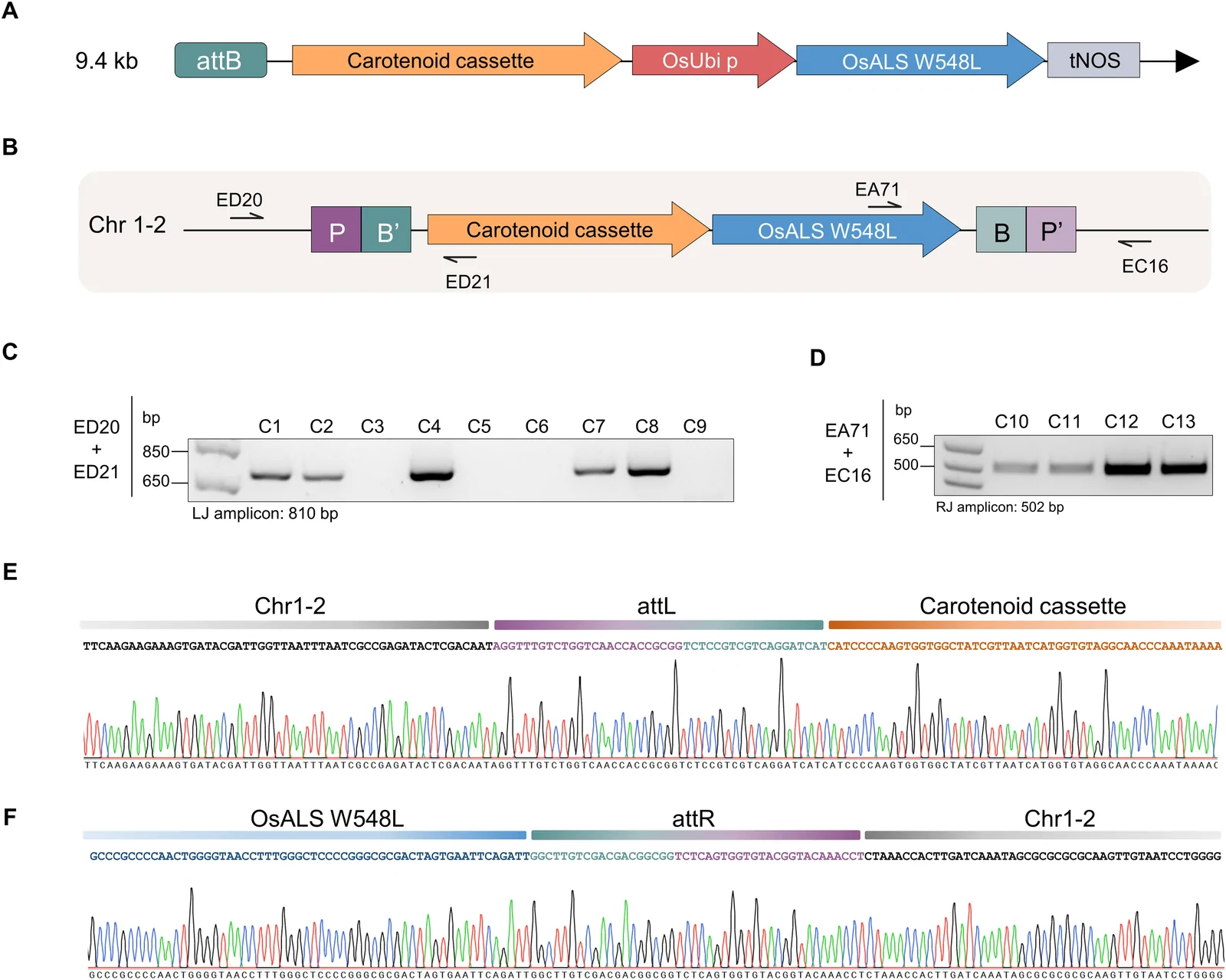
Precise Bxb1-mediated integration of the carotenoid-*OsALS* W548L cassette in callus. **(A)** Schematic of the carotenoid-*OsALS* W548L cassette used for site-specific integration. **(B)** Diagram illustrating Bxb1-mediated recombination and targeted insertion of the carotenoid-*OsALS* W548L cassette into the plant genome. **(C)** Agarose gel electrophoresis of PCR amplicons confirming the presence of the left junction in callus samples. Nontransformed callus (no Bxb1 or donor) was included as a negative control and produced no junction amplicon. **(D)** Agarose gel electrophoresis confirming the presence of the right junction. Nontransformed callus was included as a negative control. **(E)** Sanger sequencing chromatograms of the left junction and **(F)** right junction, confirming precise insertion of the carotenoid-*OsALS* W548L cassette at the Chr 1-2 genomic safe harbor.

### Site-specific integration efficiencies using engineered Bxb1 variants

Recent protein engineering efforts have generated Bxb1 variants with enhanced recombination activity in mammalian cells, including evoBxb1 and eeBxb1 [12]. We hypothesized that these variants could improve integration performance in plant cells. To evaluate this, the carotenoid donor minicircle was co-delivered with WT Bxb1, evoBxb1, or eeBxb1, and integration outcomes were quantified by junction PCR. Both recombination junctions were detected using primer pairs designed to anneal to the carotenoid cassette and the rice Chr 1-2 GSH locus (Fig 5A–5C). It should be noted that genotyping by junction-specific PCR, including with nested amplification, is inherently biased toward detecting successful integration events and does not capture un-recombined alleles in the bulk genomic DNA. Therefore, the “installation frequency” reported here reflects the proportion of sampled calli/plants in which at least one integration event was detectable by PCR, possibly down to a single template molecule, and is a qualitative rather than quantitative measure of allele-level integration efficiency. Amplicon deep sequencing (NGS) and long-read sequencing (e.g., Oxford Nanopore or PacBio) would provide an unbiased estimate of stable allelic integration frequency and enable formal comparison between Bxb1 variants.

**Fig 5:**
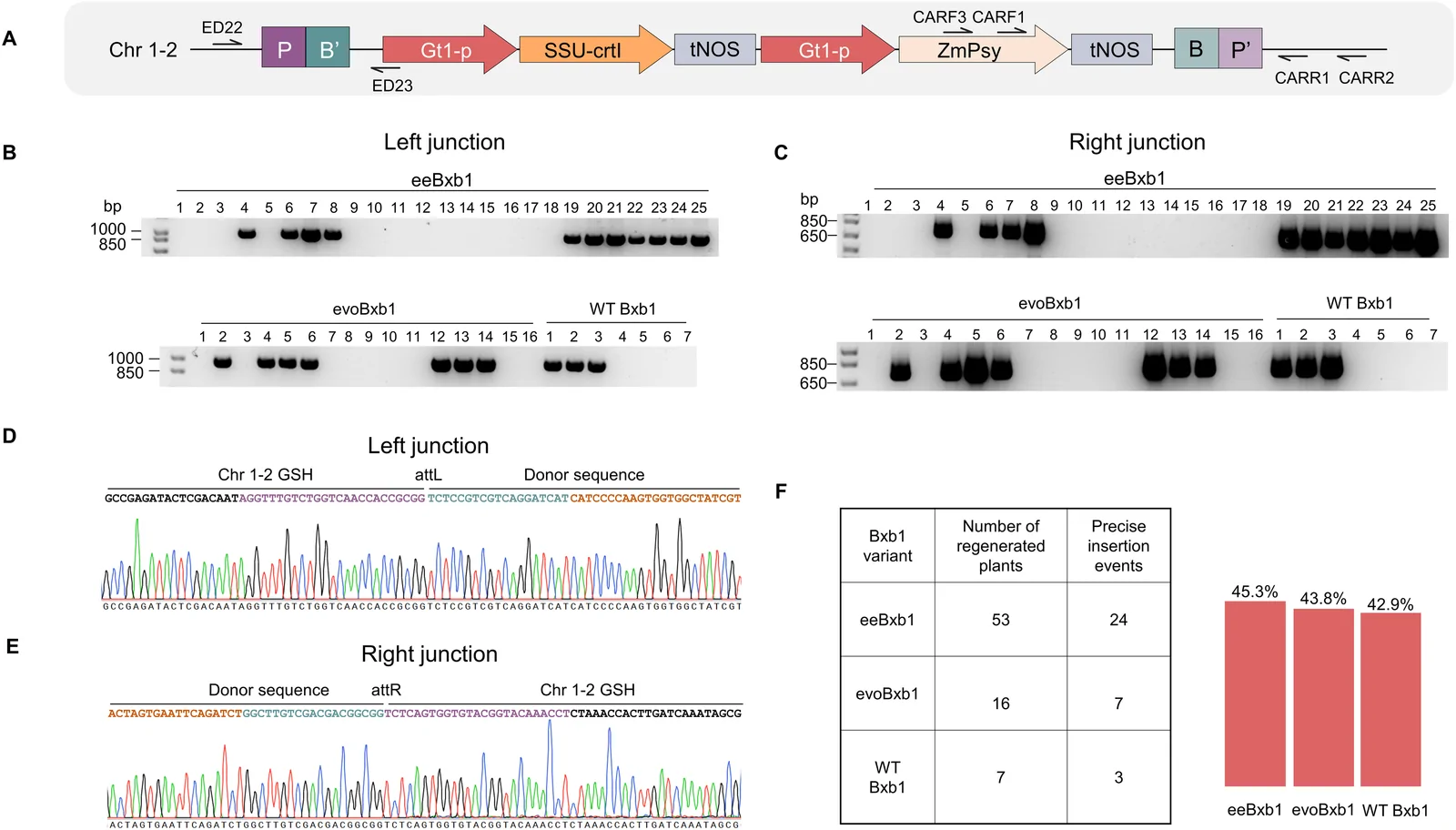
Comparison of Bxb1 recombinase variants for integration of large DNA donors in rice. **(A)** Schematic of the carotenoid biosynthesis cassette integrated into the plant genome via Bxb1-mediated recombination. Black arrows denote primer pairs used to amplify the left and right junctions. **(B)** Agarose gel electrophoresis of PCR amplicons confirming the presence of the left junction and **(C)** the right junction in regenerated plants. Nontransformed callus (no Bxb1 or donor) was included as a negative control in both panels B and C and produced no junction amplicon. In panel C, the right-junction amplicon in some samples was detected only after nested PCR; expected band sizes are indicated by molecular weight markers on the gel. Thick bands in panel C reflect PCR amplification under nested conditions; annotated ladder positions are indicated to assist size estimation. **(D)** Representative Sanger sequencing chromatograms of the left junction and **(E)** the right junction, confirming accurate and precise recombination at the attachment sites following transformation with eeBxb1, evoBxb1, or WT Bxb1. **(F)** Integration efficiency of the carotenoid cassette at the Chr 1-2 GSH, quantified in regenerated plants by junction-specific PCR and validated by Sanger sequencing. The underlying numerical data for panel F are provided in S1 Data.

All recovered sequences precisely matched the predicted recombination products generated by Bxb1-mediated integration, confirming accurate site-specific insertion (Fig 5D, 5E). For the eeBxb1 and minicircle co-delivery, 24 plants carrying validated site-specific cassette insertion were identified via junction-PCR and Sanger sequencing. Numerically, eeBxb1 showed the highest integration frequency (45.3%), followed by evoBxb1 (43.8%) and WT Bxb1 (42.9%) (Fig 5F). However, given the sample size (*n* = 53 for eeBxb1), the numerical differences among the three variants were modest, and no formal statistical test was performed; therefore, these proportions are interpreted descriptively. Accordingly, we do not conclude that eeBxb1 is statistically superior to WT Bxb1 under the conditions tested, although the trend toward higher efficiency is consistent with its improved activity in mammalian systems. The limited improvement relative to WT Bxb1 was notably smaller than enhancements reported in human cells [12], suggesting that the advantages conferred by engineered Bxb1 variants may be context dependent and influenced by delivery method, genomic locus, or host-specific constraints.

Moreover, the 34 regenerated plants previously confirmed positive for both the right and left integration junctions were further analyzed for the presence of the unrecombined *attP* allele. Amplicons corresponding to the *attP*-containing allele were detected in all 34 plants. A representative analysis showing the identification of the *attP* allele in regenerated plants has been included in S15 Fig. These results indicate that all regenerated plants positive for targeted integration also retained the unrecombined *attP*-containing allele, suggesting that the regenerated lines are likely heterozygous and/or chimeric.

Regarding the phenotypic effects associated with the integration of the 5.3 kb carotenoid cassette, no obvious morphological differences were observed between regenerated positive plants and WT controls under the evaluated growth conditions. Regenerated plants displayed normal growth and overall morphology comparable to WT plants following transfer to soil. Representative regenerated plants carrying the targeted 5.3-kb carotenoid cassette integration are shown in S16 Fig. No obvious morphological differences relative to WT plants were observed five weeks after transfer to soil. Because the carotenoid cassette is driven by endosperm-specific promoters, these vegetative-stage images were included only to document plant morphology and were not intended to assess the functional activity of the integrated cassette. Seeds derived from these T₀ plants did not display an obvious visible phenotype associated with carotenoid accumulation. Because transgene expression and carotenoid accumulation were not further evaluated, no conclusions regarding the functional activity of the integrated carotenoid cassette were drawn from this observation.

## Discussion

The precise, site-specific integration of large DNA sequences into plant genomes has long represented a formidable barrier in crop biotechnology and synthetic biology. In this study, we introduce PrimeStack, a pioneering DSB-independent platform that synergistically couples CRISPR-mediated PE with the large serine integrase Bxb1 recombinase to enable programmable, precise insertion of kilobase-scale genetic cargos into GSH. PrimeStack exploits Bxb1’s strictly unidirectional recombination to stack multigene cassettes, generating stable *attL*/*attR* hybrids that are inherently resistant to reversal without the need for exogenous factors.

PrimeRoot, the closest prior system, couples enhanced paired prime editors with Cre for DSB-free insertions of up to 11.1 kb in rice, enabling promoter swaps and marker-free disease resistance stacking at efficiencies of 6–8% [13]. However, Cre’s bidirectional nature necessitates transient expression or segregation to avert instability, constraints absent in PrimeStack’s unidirectional framework. Similarly, the mammalian PASSIGE/eePASSIGE system, employing PACE-evolved Bxb1 for 23%–30% gene-sized integrations in human cells [12], provides a blueprint for PrimeStack’s enhancements. Evolved and engineered Bxb1 recombinase variants (evoBxb1 and eeBxb1) have previously been reported to mediate up to ~60% donor integration in human cell lines [12], corresponding to multi-fold improvements over WT Bxb1. In contrast, application of the PE–Bxb1 system in rice yielded integration frequencies of 42.9–45.3% across Bxb1 variants, with eeBxb1 achieving the highest frequency (45.3%), followed closely by evoBxb1 (43.8%) and wtBxb1 (42.9%) as detected by junction PCR.

Notably, the relative performance differences among Bxb1 variants in plants were modest, in contrast to the more pronounced gains reported in mammalian systems. This suggests that, in plant cells, host-specific constraints, delivery modality (particle bombardment), tissue culture context, and regeneration capacity may play a dominant role in limiting recombination outcomes, thereby reducing the apparent benefit of engineered recombinase variants. Importantly, despite these constraints, our platform supported robust site-specific integration, demonstrating that high-efficiency recombinase-mediated cassette integration is achievable in plants using a marker-free minicircle donor cargo.

Several limitations of the current study warrant acknowledgement and point toward key future priorities. First, genotyping was performed exclusively by junction-specific PCR and Sanger sequencing; while this confirms precise recombination at the targeted locus, it cannot exclude additional random integration of minicircle donor DNA elsewhere in the genome via illegitimate recombination. Whole-genome sequencing (WGS) of a representative set of regenerated plants will be required to comprehensively assess integration specificity and exclude off-target insertion events, a particularly important consideration given the use of biolistic delivery, which can introduce multiple DNA copies simultaneously. Second, two of our candidate loci were located within the 25S rDNA region. Because the rice genome harbors hundreds of tandemly repeated rDNA units on chromosome 9, PE at these sites may target multiple loci simultaneously, and the precise number and chromosomal identity of targeted copies may vary between experiments. The rDNA-targeted results should therefore be regarded as exploratory; future experiments should incorporate long-read WGS or fiber-FISH to confirm single-locus targeting. Third, the Bxb1 *attP* and *attB* recognition sequences used here are 38–50 bp in length. This length is relevant because the European Commission’s proposal on New Genomic Techniques (NGTs) (COM(2023) 411) generally limits insertions or substitutions in Category 1 NGT plants to no more than 20 nucleotides. Therefore, lines carrying these *attP* sites may not fall within these criteria and could remain subject to full GMO regulatory oversight depending on the jurisdiction and final genomic configuration of the engineered plants [75]. Engineering shorter functional *att* site variants, or adopting recombinase systems with compact recognition sequences compatible with such thresholds, represents an important future direction for translational deployment in regulated markets. Fourth, the current PrimeStack workflow is inherently stepwise: it requires (i) *attP* installation by PE with selection and plant regeneration, (ii) T-DNA segregation to recover transgene-free germplasm, and (iii) a second biolistic transformation with the donor and integrase, a process that is labor-intensive and time-consuming. An “all-in-one” co-delivery strategy that simultaneously provides the PE machinery, the Bxb1 integrase, and the donor DNA in a single transformation event, as demonstrated for PrimeRoot and the recently described PCE system [14], would substantially streamline the workflow and broaden its practical applicability. Fifth, a direct quantitative comparison of PrimeStack efficiency with Cre-lox-mediated approaches at identical loci and with matched cargo sizes was not performed here; such head-to-head experiments are warranted in follow-up work. Sixth, the recently reported PCE system [14] demonstrates that the bidirectionality limitation of classical Cre-lox can be overcome in plants, further expanding the toolkit for precision plant genome engineering and warranting systematic comparative evaluation against Bxb1-based approaches. Seventh, although targeted integration of the 5.3-kb carotenoid cassette was supported by junction-specific PCR and Sanger sequencing of both recombination junctions, the present study did not evaluate transgene expression or carotenoid accumulation. Seeds obtained from the regenerated T_0_ plants did not display an obvious visible phenotype associated with carotenoid accumulation; however, visual inspection alone is insufficient to determine the functional activity of the integrated pathway. Future studies should assess cassette expression, quantify carotenoid composition and accumulation, and evaluate the stability and inheritance of the integrated cassette across generations. Accordingly, the present results demonstrate the technical feasibility of targeted multikilobase DNA integration in rice but do not establish functional carotenoid biofortification.

PrimeStack’s emphasis on GSH engineering amplifies its utility (S17 Fig. [76]). These loci are intended to support consistent transgene expression while minimizing disruption of endogenous functions, mitigating biosafety concerns, and facilitating multiplexed trait stacking for synthetic biology applications, such as biofactories or phytoremediation. PrimeStack offers a paradigm shift toward predictive, design-centric plant engineering. In crop biotechnology, it could facilitate the prototyping of climate-adaptive varieties by integrating nitrogen-use efficiency modules, carbon-sequestration pathways, or drought-tolerance networks into elite germplasm. Notably, precise GSH-targeted integration of a carotenoid biosynthesis cassette establishes a technical foundation for future evaluation of carotenoid biofortification in rice, highlighting the potential of PrimeStack for sustainable crop intensification. In plant synthetic biology, PrimeStack provides a powerful chassis for assembling orthogonal circuits, biosensors, and reprogrammed metabolisms at reliable loci. Future iterations, incorporating AI-guided Bxb1 optimizations and expanded GSH repertoires, promise to further amplify its scope, cementing its role in reshaping targeted trait engineering in diverse crop species.

## Materials and methods

### Plant material

Mature dry seeds of *Oryza sativa* ssp. *japonica* cv. Nipponbare were used as explants for callus induction. Seeds were dehusked and surface-sterilized with 70% (v/v) ethanol for 1 min, followed by incubation in 80% (v/v) commercial bleach solution containing one drop of Tween 20 for 1 hour with continuous shaking. Seeds were rinsed five times with sterile distilled water and placed on 2N6 medium [77] for callus induction.

### General methods

Gibson assembly was used to clone all the prime editors, Bxb1 variants and parental plasmid (PP) using NEBuilder HiFi DNA assembly master mix (New England Biolabs). PCR was performed using Phusion High-Fidelity DNA Polymerase (New England Biolabs), Q5 High-Fidelity DNA Polymerase (New England Biolabs), or Phire Plant Direct PCR Master Mix (ThermoFisher). DNA oligonucleotides were obtained from Integrated DNA Technologies (IDT). All PCR products were purified using the QIAquick PCR Purification Kit (QIAGEN). Dual-epegRNAs were obtained from Genscript. All vectors were purified using QIAprep Spin Miniprep kits (Qiagen). All plasmids used in this study are presented in S1 Table. All PCR primers used in this study are presented in S2 Table.

### Prime editor plasmid construction and cloning strategy

DNA fragments encoding prime editors and epegRNAs were cloned into the pCREATE-51 (pC-51) backbone. pC-51 is a modified plasmid derived from pRGEB32 that enables expression of the prime editors PE6c, PE6d, and PEmax under the maize Ubiquitin promoter with a NOS terminator, and the pegRNAs under the OsU3 promoter and the Pol III terminator. The PE6c, PE6d, and PEmax template plasmids were obtained from Addgene (#207853, #207854, and #174820). Ligation of each epegRNA cassette into the corresponding intermediate backbone yielded a total of 30 prime editor constructs, 10 per editor variant, each designed to target the 10 selected genomic regions (S1 Fig).

### Bxb1 plasmid cloning

For expression of the Bxb1 variants, the pCREATE-51 plasmid was digested with BsrGI and SacI. Addgene plasmids encoding WT Bxb1, evoBxb1, and eeBxb1 (#222337, #222338, and #222339) were digested with EcoRI, gel-purified, and ligated into the prepared backbone to generate three expression vectors (pC-51-WT-Bxb1, pC-51-evoBxb1, and pC-51-eeBxb1). Each final construct drives expression of the corresponding Bxb1 variant under the rice Ubiquitin promoter with a NOS terminator (S2 Fig).

### Minicircle donor production

The minicircle donors were prepared using the MC-Easy Minicircle DNA Production Kit (System Biosciences, MA925A-1). The PPs were cloned into the *E. coli* ZYCY10P3S2T minicircle producer strain. A single colony was inoculated into 2 mL of LB medium supplemented with 50 μg/mL kanamycin and incubated at 30 °C for 4 hours. Subsequently, 1 mL of the starter culture was inoculated into 200 mL of TB medium and grown overnight at 30 °C (10–12 hours) until reaching OD600 4–6. Minicircle induction was initiated by adjusting the culture pH to 7.0 and adding 200 mL of induction medium. Minicircle DNA was purified using the Qiagen Plasmid Maxi kit according to the manufacturer’s instructions. Minicircle quality and integrity were assessed by restriction enzyme digestion and visualization on a 1% agarose gel.

### *Agrobacterium*-mediated rice transformation for *attP* installation

A total of 30 plasmids encoding prime editors and pegRNAs were transformed into *A. tumefaciens* strain EHA105 by electroporation. Calli derived from mature seeds were pre-cultured on fresh 2N6 medium at 32 °C under continuous light for 3 days and then immersed in the *Agrobacterium* suspension for 2 min. Following co-cultivation at 28 °C in the dark for 3 days, calli were transferred to selection medium containing 40 μg/mL hygromycin for 2 weeks, and subsequently moved to a second selection medium containing 50 μg/mL hygromycin for 5 days. Resistant calli were transferred to N6RH50 regeneration medium. Regenerated shoots were moved to N6FH50 medium for root development and successfully rooted plantlets were transferred to soil and screened by PCR to identify *attP* insertion events [77].

### Identification of *attP* insertion events

Genomic DNA was extracted from calli 19 days post-transformation under selection or from regenerated plantlets. Transgenic tissue was PCR-genotyped to identify *attP* insertion events. For edited amplicons differing from wild type by 8 bp or less, HincII restriction digestion was used to detect the restriction site introduced within the *attP* sequence and thereby support identification of *attP*-containing alleles. Amplicons were resolved on 1–3% agarose gel, excised, purified, cloned into the pJET1.2/blunt vector, and submitted for Sanger sequencing. Sequencing chromatograms were aligned to reference sequences using SnapGene Software (GSL Biotech) to confirm precise installation and sequence integrity of the inserted Bxb1 *attP* site. Primer sequences for each target and the expected sizes of WT and edited amplicons are provided in S4 Table.

### Generation of homozygous *attP* and transgene-free lines

T_1_ progeny of heterozygous and chimeric T_0_ events were screened by PCR to identify homozygous *attP* individuals. In parallel, T_1_ plants were genotyped to identify individuals that had segregated away the T-DNA. Selected T_1_ plants were grown to maturity, and T_2_ seeds were collected for subsequent experimental procedures.

### Biolistic co-delivery of minicircle donors and Bxb1 into *attP*-engineered rice callus

Rice calli harboring Bxb1 *attP* sites were subjected to biolistic co-delivery of minicircle donor DNA and plasmids expressing Bxb1 variants. For each bombardment, 1 μg of minicircle DNA and 1 μg of Bxb1 plasmid were mixed with 50 μL of 0.6 μm gold particles (60 μg/μL), followed by addition of 50 μL of 2.5 M CaCl_2_ and 20 μL of 0.1 M spermidine. Bombardment was performed using a Bio-Rad PDS-1000/He particle delivery system equipped with 1,100 psi rupture disks. Bombarded calli were transferred to 2NBK medium containing 40 μg/mL hygromycin and incubated at 32 °C under continuous light for 2 weeks, then moved to nN6C selection medium with 50 μg/mL hygromycin for 5 days before transfer to R8 regeneration medium [77–79]. Regenerated plants were screened by PCR for site-specific integration.

### Analysis of Bxb1-mediated long DNA fragment integration

Genomic DNA was extracted from calli 19 days post-transformation under selection or from regenerated plantlets. The left and right genome-donor junctions were PCR amplified and resolved by 1% agarose gel electrophoresis, excised, purified, cloned into the pJET1.2/blunt vector, and submitted for Sanger sequencing. Sequencing chromatograms were aligned to reference sequences using SnapGene Software (GSL Biotech; www.snapgene.com) to confirm junction accuracy and detect potential sequence alterations.

### Genomic DNA extraction from plant tissues

Frozen plant leaves were ground to a fine powder, whereas callus tissue was homogenized to release cellular contents. Samples were resuspended in extraction buffer (0.1 M Tris-HCl pH 8.0, 1 mM EDTA, 0.1 M NaCl, 0.1 M LiCl, 0.1 M β-mercaptoethanol, 0.4% [w/v] RNase I) at a ratio of 500 μL per 300 μL of leaf tissue or five callus pieces. Samples were subjected to phenol:chloroform:isoamyl alcohol extraction, and DNA was precipitated with absolute ethanol and 30 μL of 3 M sodium acetate (pH 5.2). Pellets were washed twice with 70% ethanol, air-dried, and resuspended in nuclease-free water. DNA concentration was assessed by NanoDrop UV/Vis spectrophotometry.

## Supporting information

S1 Fig Prime editor constructs for *attP* installation at 10 genomic targets. Schematic representation of the pCREATE-51-based constructs encoding PE6c, PE6d, and PEmax prime editors with dual-epegRNA cassettes targeting each of the 10 selected loci. Expression cassettes for the prime editor protein and dual epegRNAs are shown.
(TIF)

S2 Fig Bxb1 expression vector maps. Schematics of the pCREATE-51-based constructs encoding WT Bxb1, evoBxb1, and eeBxb1 recombinases under the rice Ubiquitin promoter.
(TIF)

S3 Fig Cloning strategy overview for parental plasmids. Schematic depicting the sequential assembly steps for PP-Carotenoid, PP-*OsALS* W548L, and PP-Carotenoid-*OsALS* W548L parental constructs.
(TIF)

S4 Fig Dual-epegRNA cassette architecture. **Structure and sequence details for the dual-epegRNA cassettes used for *attP* installation, including spacer sequences, PBS lengths, RT template sequences, and tevopreQ1 stabilizing motif positions for each of the 10 target loci**.
(TIF)

S5 Fig *attP* installation at the *OsMOC1* promoter locus. (A) PCR results showing *attP*-containing amplicons at the *OsMOC1* locus. (B) Sanger sequencing chromatogram confirming precise *attP* insertion near the *OsMOC1* promoter. (C) Installation efficiency at the *OsMOC1* locus. (D) T-DNA segregation analysis in T1 progeny derived from the *attP*-*OsMOC1* event.
(TIF)

S6 Fig Sanger sequencing chromatograms confirming *attP* insertion at Chr 3, Chr 7, and Chr 11 GSHs. Representative chromatograms showing accurate and complete *attP* insertion sequences at each locus.
(TIF)

S7 Fig Characterization of aberrant amplicons at Chr 3 and Chr 7. Sanger sequencing chromatograms of amplicons deviating from expected size at the Chr 3 and Chr 7 GSHs, indicating defective or rearranged versions of edited or wild-type alleles.
(TIF)

S8 Fig Sequence analysis of *OsTT1* candidate events. Sanger sequencing data from six candidate homozygous events at the *OsTT1* locus showing a 15-bp deletion within the *attP* sequence.
(TIF)

S9 Fig T-DNA segregation analysis at Chr 1-2. PCR-based genotyping of T1 progeny from the homozygous *attP* Chr 1-2 event showing T-DNA segregation and identification of T-DNA-free individuals.
(TIF)

S10 Fig T1 segregation analysis at additional loci. (A) PCR screening of T1 progeny from *attP*-Chr 11 event. (B, C) Genotyping of heterozygous and chimeric T0 events advanced to T1. (D) Confirmation of T-DNA retention in T1 plants.
(TIF)

S11 Fig Sequence integrity of *attP* in T1 homozygotes. Sanger sequencing confirming accurate *attP* sequence in T1 homozygous lines derived from heterozygous T0 events.
(TIF)

S12 Fig [80] Minicircle production and validation. (A) Parental plasmid structure for carotenoid minicircle production. (B) Minicircle induction protocol overview. (C, D) Restriction enzyme digestion confirming identity of parental plasmid and resulting minicircle. (E) Bxb1 expression vector schematics. (F) Diagnostic restriction digestion of maxiprep-purified Bxb1 recombinase variants. Created in BioRender. Sanchez Hernandez, E. (2026) https://BioRender.com/8knw1ak.
(TIF)

S13 Fig Validation of the carotenoid-*OsALS* W548L minicircle donor. (A) Schematics of the parental plasmid and minicircle donor. Positions of the XmnI and ApaI sites used for diagnostic digestion are indicated. (B) Restriction enzyme digestion profiles confirming the identity of the 13.6-kb parental plasmid and the 9.4-kb minicircle donor.
(TIF)

S14 Fig Junction PCR analysis of eeBxb1-regenerated plants. A total of 53 plants were regenerated following co-delivery of eeBxb1 and the carotenoid minicircle donor; (A) left and (B) right junction-PCR results for the complete set of regenerated plants are shown.
(TIF)

S15 Fig Detection of unrecombined *attP* alleles in regenerated plants carrying targeted carotenoid cassette integration. PCR analysis was performed on the 34 regenerated plants previously confirmed to be positive for both left and right integration junctions. Using primers EC13 and EC14, clear 263 bp amplicons corresponding to the unrecombined *attP*-containing allele were detected in all analyzed plants. *attP* Ctrl; the homozygous plants carrying *attP* sites were used as a positive control.
(TIF)

S16 Fig Representative regenerated rice plants carrying targeted integration of the 5.3 kb carotenoid cassette mediated by eeBxb1 (plant 4, corresponds to main Fig 5) or evoBxb1 (plant 2, corresponds to main Fig 5) recombinases, shown 5 weeks after transfer to soil. WT, wild-type control plant. No obvious morphological differences were observed between regenerated and WT plants under the evaluated growth conditions.
(TIF)

S17 Fig [76] Graphical abstract. Schematic overview of the PrimeStack platform for targeted large DNA integration in plants. In the first step, Twin prime editing (TwinPE) installs a Bxb1 *attP* recombination site at a predefined genomic safe harbor (GSH) in rice using paired epegRNAs and a prime editor, generating a landing pad without introducing double-strand breaks. In the second step, *attP*-containing seeds are subjected to callus induction and biolistic co-delivery of an *attB*-containing minicircle donor carrying the gene of interest (GOI) together with eeBxb1, evoBxb1, or wild-type (WT) Bxb1 recombinase, resulting in site-specific integration of large DNA cargos at the engineered genomic locus. *Created in BioRen*der. Sanchez Hernandez, E. (2026) https://BioRender.com/kwbygr8.
(TIF)

S1 Table List of all plasmids used in this study. (XLSX)

S2 Table List of all PCR primers used in this study. (XLSX)

S3 Table List of synthesized dual-epegRNA sequences used for *attP* installation. (XLSX)

S4 Table Primer sequences for genotyping each target locus and expected WT and edited amplicon sizes. (XLSX)

S5 Table Precise genomic coordinates of each installed *attP* site. (XLSX)

S6 Table Sequences of dual-epegRNA cassettes for all 10 targets. (XLSX)

S7 Table Genotyping outcomes of independently regenerated rice lines obtained with each prime editor variant (PE6c, PE6d, and PEmax). The table includes the total number of independent lines analyzed; the numbers of precisely edited lines classified as homozygous, heterozygous, chimeric, or of undetermined zygosity; and the numbers of nonprecisely edited and WT lines.
(XLSX)

S8 Table Detection of 9.4-kb targeted integration in rice callus using eeBxb1, evoBxb1, and WT Bxb1. For each recombinase variant, 10 callus pieces were pooled to constitute one sample, and 10 pooled samples were genotyped. The percentage of positive samples was calculated as the number of pooled samples in which targeted integration was detected divided by the total number of pooled samples analyzed.
(XLSX)

S9 Table Source for Fig 2C. Number of independently regenerated plants carrying correctly installed *attP* alleles generated using the three prime editor variants (PE6c, PE6d, and PEmax) across the four genomic target loci (Chr 1-2, Chr 3, Chr 7, and Chr 11).
(XLSX)

S10 Table Source for Fig 2D. Number of regenerated plants carrying correctly installed *attP* alleles at each genomic target locus (Chr 1-2, Chr 3, Chr 7, and Chr 11) following PE6c-mediated editing, together with the total number of regenerated plants screened and the corresponding *attP* installation efficiency (%). Efficiency was calculated as the percentage of regenerated plants containing the precise *attP* insertion relative to the total number of regenerated plants analyzed for each target locus. The data presented in this table were used to generate the bar graph shown in Fig 2D.
(XLSX)

S11 Table Source for Fig 2E. Distribution of precisely edited plants according to editing outcome (homozygous, heterozygous, or chimera) for each genomic target locus (Chr 1-2, Chr 3, Chr 7, and Chr 11) following PE6c-mediated *attP* installation. Classification was based on molecular genotyping of plants carrying the correct *attP* insertion. The total number of correctly edited plants analyzed at each locus is indicated in the final column. The data presented in this table were used to generate the pie charts shown in Fig 2E.
(XLSX)

S12 Table GenBank accession numbers for Sanger sequencing records generated in this study. This table maps each PrimeStack sequence identifier to its corresponding GenBank accession number and provides the associated sample type, targeted locus, and sequence content.
(XLSX)

S1 Data Numerical source data underlying Figs 2C–2E and 5F. Excel workbook containing four data worksheets corresponding to the indicated figure panels. Fig 2C worksheet: numbers of independently regenerated rice plants carrying correctly installed *attP* alleles following editing with PE6c, PE6d, or PEmax at the Chr 1-2, Chr 3, Chr 7, and Chr 11 loci. Fig 2D worksheet: numbers of precise *attP*-positive plants, the total numbers of regenerated plants screened, and the calculated PE6c-mediated installation frequencies at each locus. Fig 2E worksheet: numbers of homozygous, heterozygous, and chimeric plants among precisely edited PE6c plants. Fig 5F worksheet: numbers of validated integration-positive plants, total plants screened, and carotenoid cassette integration frequencies for WT Bxb1, evoBxb1, and eeBxb1. Integration was assessed by junction-specific PCR and validated by Sanger sequencing. Frequencies were calculated by dividing the number of positive plants by the total number screened for the corresponding locus or recombinase variant.
(XLSX)

S1 Raw Images Source for Fig 2A. Original, uncropped gel corresponding to Fig 2A, showing PCR analysis of *attP* installation at the Chr 1-2, Chr 3, Chr 7, and Chr 11 loci. **Source for Fig 2F, samples 1–16.** Original, uncropped gels showing PCR analysis of *attP* inheritance and T-DNA segregation in T1 progeny derived from a homozygous *attP* Chr 1-2 event. **Source for Fig 2F, samples 17–32.** Original, uncropped gels showing PCR analysis of *attP* inheritance and T-DNA segregation in T1 progeny derived from a homozygous *attP* Chr1–2 event. **Source for Figs 3C and 4C.** Original, uncropped gels showing left junction-PCR analysis of targeted integration events in rice callus. The lower gel corresponds to the carotenoid cassette in Fig 3C, and the upper gel corresponds to the carotenoid-*OsALS* W548L cassette in Fig 4C. **Source for Fig 4D.** Original, uncropped gel showing right-junction PCR analysis of the carotenoid-*OsALS* W548L cassette in rice callus samples C10–C13. **Source for Fig 5B, eeBxb1 samples.** Original, uncropped gels showing left-junction PCR analysis of regenerated plants obtained following transformation with eeBxb1 and the carotenoid minicircle donor. **Source for Fig 5C, eeBxb1 samples.** Original, uncropped gels showing right-junction PCR analysis of regenerated plants obtained following transformation with eeBxb1 and the carotenoid minicircle donor. **Source for Fig 5B, evoBxb1 and WT Bxb1 samples.** Original, uncropped gels showing left-junction PCR analysis of regenerated plants obtained using evoBxb1 and WT Bxb1 with the carotenoid minicircle donor. **Source for Fig 5C, evoBxb1 and WT Bxb1 samples.** Original, uncropped gels showing right-junction PCR analysis of regenerated plants obtained using evoBxb1 and WT Bxb1 with the carotenoid minicircle donor. **Source for** **S5A Fig**. Original, uncropped gel showing PCR screening for *attP* installation at the *OsMOC1* promoter locus in regenerated rice plants. **Source for** **S5A Fig** **(HincII digestion).** Original, uncropped gel showing HincII digestion of PCR amplicons used to validate *attP* installation at the *OsMOC1* promoter locus. Digestion of the *attP*-containing amplicon produces the expected 192-bp and 143-bp fragments. **Source for** **S5D Fig****, samples 1–16**. ***attP-OsMOC1* genotyping.** Original, uncropped gel showing PCR genotyping of the *attP-OsMOC1* allele in T1 progeny. **Source for** **S5D Fig****, samples 17–38. *attP-OsMOC1* genotyping.** Original, uncropped gel showing PCR genotyping of the *attP-OsMOC1* allele in T1 progeny. **Source for** **S5D Fig****, samples 1–38. T-DNA segregation.** Original, uncropped gel showing PCR-based T-DNA segregation analysis in T1 progeny derived from the *attP-OsMOC1* event. **Source for** **S7A** **and** **S8B Figs**. Original, uncropped gel containing the following source images. **S7A:** PCR amplicons from the Chr7 and Chr3 genomic safe-harbor loci. **S8B:** PCR amplicon from the 5′ UTR of *OsTT1*. **Source for** **S10A Fig****, samples 1–23.** Original, uncropped gel showing PCR screening for T-DNA presence in T1 progeny carrying *attP* at the Chr11 genomic safe-harbor locus. **Source for** **S10A Fig****, samples 24–38.** Original, uncropped gel showing PCR screening for T-DNA presence in T1 progeny carrying *attP* at the Chr 11 genomic safe-harbor locus. **Source for** **S10B Fig****, samples 1–16. *attP* GSH Chr3 genotyping.** Original, uncropped gel showing PCR genotyping of the *attP* allele at the Chr 3 genomic safe-harbor locus in T1 progeny. **Source for** **S10B Fig****, samples 17–42. *attP* GSH Chr 3 genotyping.** Original, uncropped gel showing PCR genotyping of the *attP* allele at the Chr3 genomic safe-harbor locus in T1 progeny. **Source for** **S10B Fig****, samples 1–32. T-DNA segregation.** Original, uncropped gel showing PCR screening for T-DNA presence in T1 progeny derived from a line carrying *attP* at the Chr 3 genomic safe-harbor locus. **Source for** **S10B Fig****, samples 33–42. T-DNA segregation.** Original, uncropped gel showing PCR screening for T-DNA presence in T1 progeny derived from a line carrying *attP* at the Chr 3 genomic safe-harbor locus. **Source for** **S10C Fig****, samples 1–32.** Original, uncropped gel used to genotype T1 samples 1–32 for *attP* installation at the Chr 7 target locus. **Source for** **S10C Fig****, samples 33–38.** Original, uncropped gel used to genotype T1 samples 33–38 for *attP* installation at the Chr 7 target locus. **Source for** **S10C Fig****, samples 1–38.** Original, uncropped gel used to screen for T-DNA presence in T1 samples derived from the line targeted for *attP* installation at the Chr 7 locus. **Source for** **S10C Fig****, samples 39–70.** Original, uncropped gel used to genotype T1 samples 39–70 for *attP* installation at the Chr 7 target locus. **Source for** **S10C Fig****, samples 39–70.** Original, uncropped gel used to screen for T-DNA presence in T1 samples derived from the line targeted for *attP* installation at the Chr 7 locus. **Source for** **S10D Fig**. Original, uncropped gel showing repeat T-DNA PCR analysis of initially negative events from the Chr 3 and Chr 7 target loci, confirming T-DNA presence in these lines. **Source for** **S12D Fig**. Original, uncropped gel showing restriction analysis of the carotenoid parental plasmid (PP) and its derived minicircle following digestion with AvrII alone or AvrII and XmnI. **Source for** **S12F Fig**. Original, uncropped gel showing diagnostic restriction digestion of maxiprep-purified plasmids encoding WT Bxb1, evoBxb1, and eeBxb1, confirming their expected construct identities. **Source for** **S13B Fig**. Original, uncropped gel showing diagnostic restriction analysis of the carotenoid-*OsALS* W548L parental plasmid (PP) and its derived minicircle (MC) following digestion with ApaI or XmnI. **Source for** **S14A Fig****, samples 26–28.** Original, uncropped gel showing left-junction PCR analysis of eeBxb1-regenerated plants 26–28. **Source for** **S14A Fig****, samples 29–53.** Original, uncropped gel showing left-junction PCR analysis of eeBxb1-regenerated plants 29–53. **Source for** **S14B Fig****, samples 26–53.** Original, uncropped gel showing right-junction PCR analysis of eeBxb1-regenerated plants 26–53. **Source for** **S15 Fig****, samples 1–17.** Original, uncropped gel showing unrecombined *attP* alleles in regenerated plants carrying targeted carotenoid cassette integration. **Source for** **S15 Fig****, samples 18–34.** Original, uncropped gel showing unrecombined *attP* alleles in regenerated plants carrying targeted carotenoid cassette integration.
(PDF)

## Abbreviations

DSBs: double-strand breaks
GOI: gene of interest
GSH: genomic safe harbors
IDT: Integrated DNA Technologies
KAUST: King Abdullah University of Science and Technology
NGTs: New Genomic Techniques
PASSIGE: prime-editing-assisted site-specific integrase gene editing
PE: Prime editing
PP: parental plasmid
RT: reverse-transcription
SSRs: Site-specific recombinases
SSU: RUBISCO small subunit
WGS: Whole-genome sequencing
WT: wild-type.
