# Supplementary material for: PrimeStack editors enable efficient and site-specific integration of large multigene cargos in plants
Source: PLoS Biol. 2026 Oct 13;24(10):e3003778. doi: 10.1371/journal.pbio.3003778 (PMC13693989; doi:10.1371/journal.pbio.3003778)
Supplement: Supplementary file 1 [file pbio.3003778.s031.pdf]

Source for Fig 2. Panel A

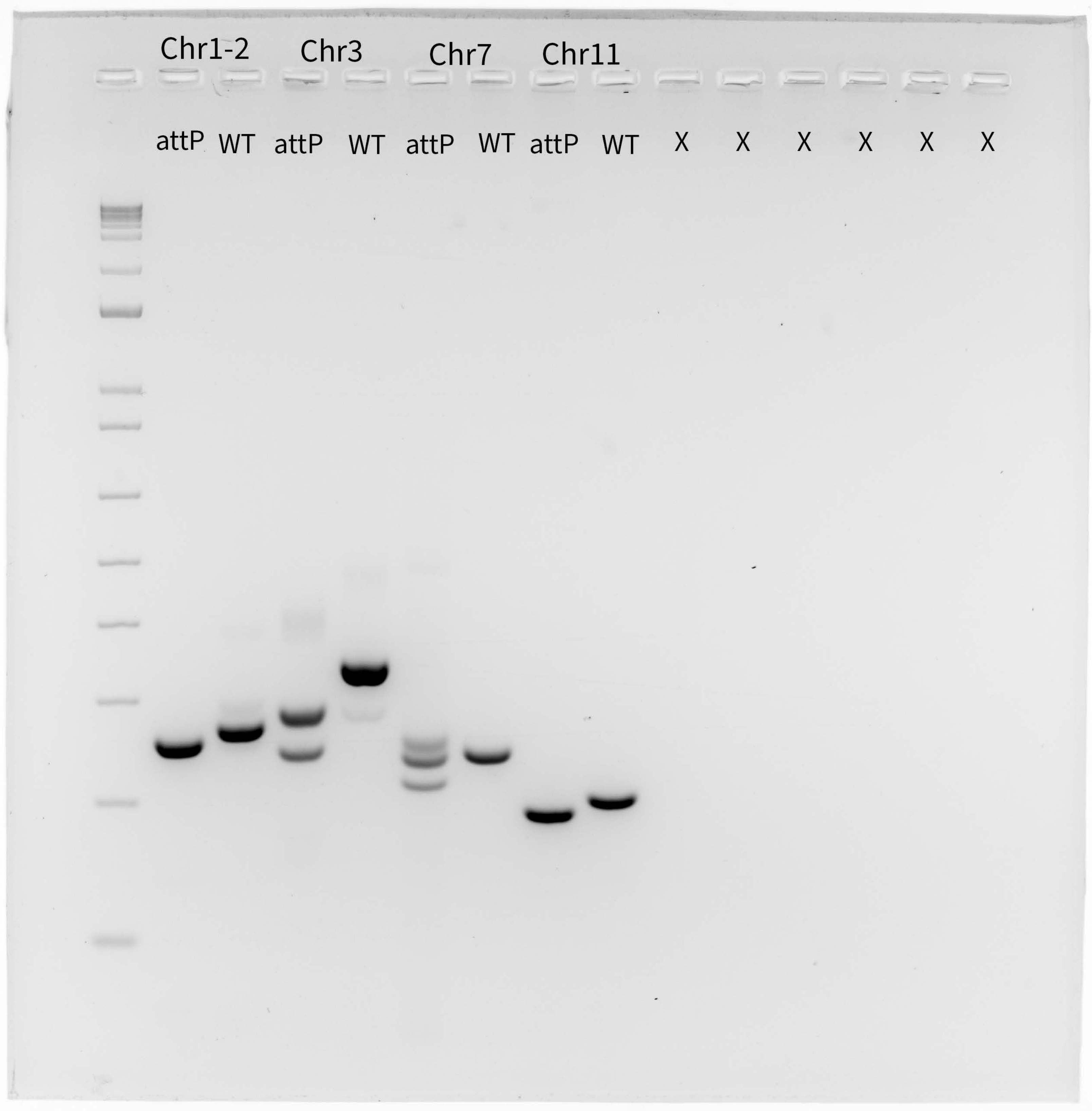

A 1 Kb Plus DNA Ladder (Invitrogen™, Thermo Fisher Scientific) was loaded in lane M and used as the molecular weight standard for estimation of PCR amplicon sizes. Gel images were acquired using an Invitrogen iBright™ 1500 Imaging System under standard imaging conditions. This original gel corresponds to Figure 2, panel A. Lane identities are indicated above the gel, and any lanes not included in the final figure are marked with an "X" above the corresponding lane label. All annotations were added without obscuring any bands, background signal, or other image features

Source for Fig 2. Panel F. Samples 1-16

attP

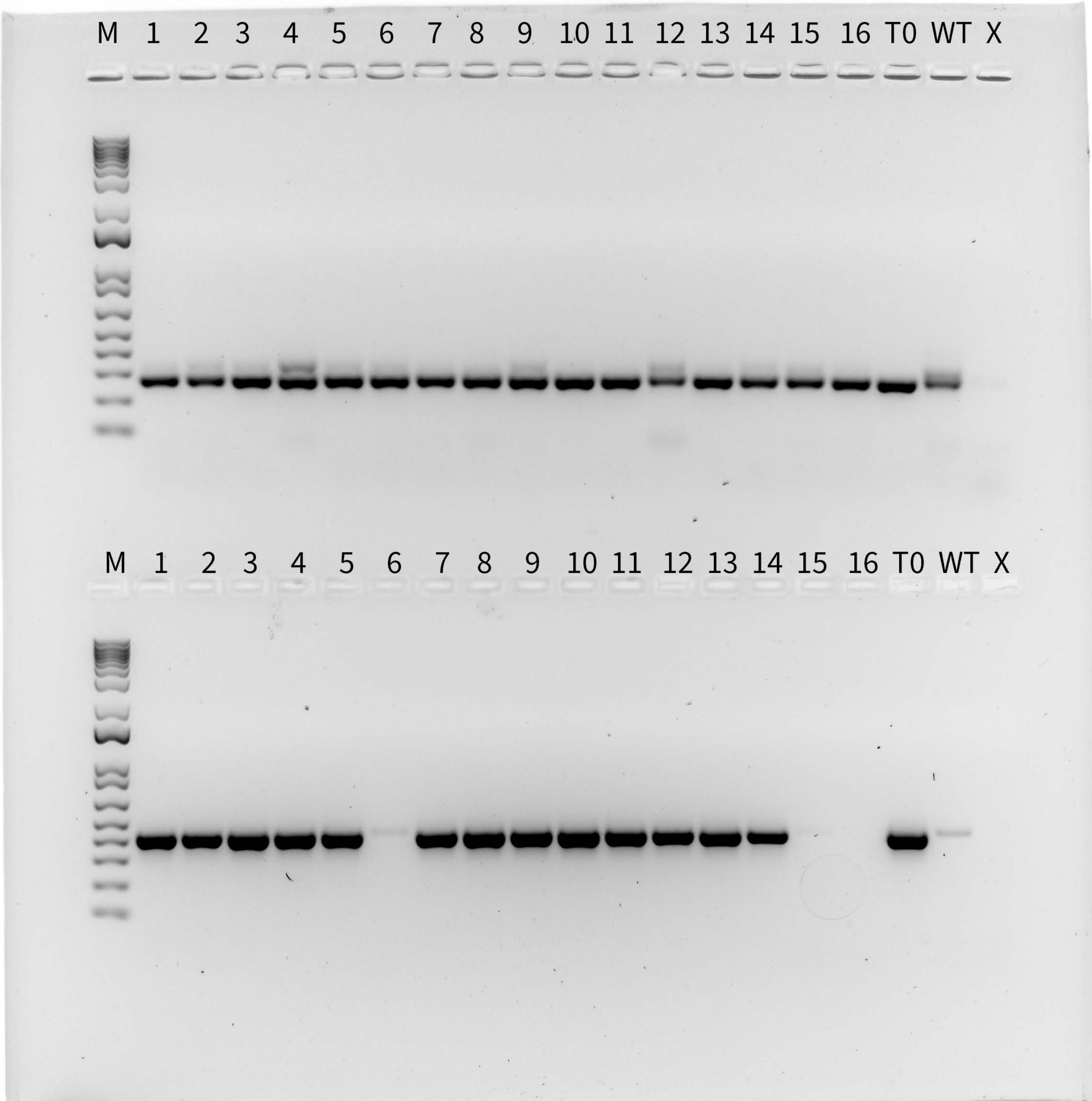

T-DNA

A 1 Kb Plus DNA Ladder (Invitrogen™, Thermo Fisher Scientific) was loaded in lane M and used as the molecular weight standard for estimation of PCR amplicon sizes. Gel images were acquired using an Invitrogen iBright™ 1500 Imaging System under standard imaging conditions. This original gel corresponds to Figure 2, panel F of the manuscript. Lane identities are indicated above the gel, and any lanes not included in the final figure are marked with an "X" above the corresponding lane label. All annotations were added without obscuring any bands, background signal, or other image features

Source for Fig 2. Panel F. Samples 17-32

attP

T-DNA

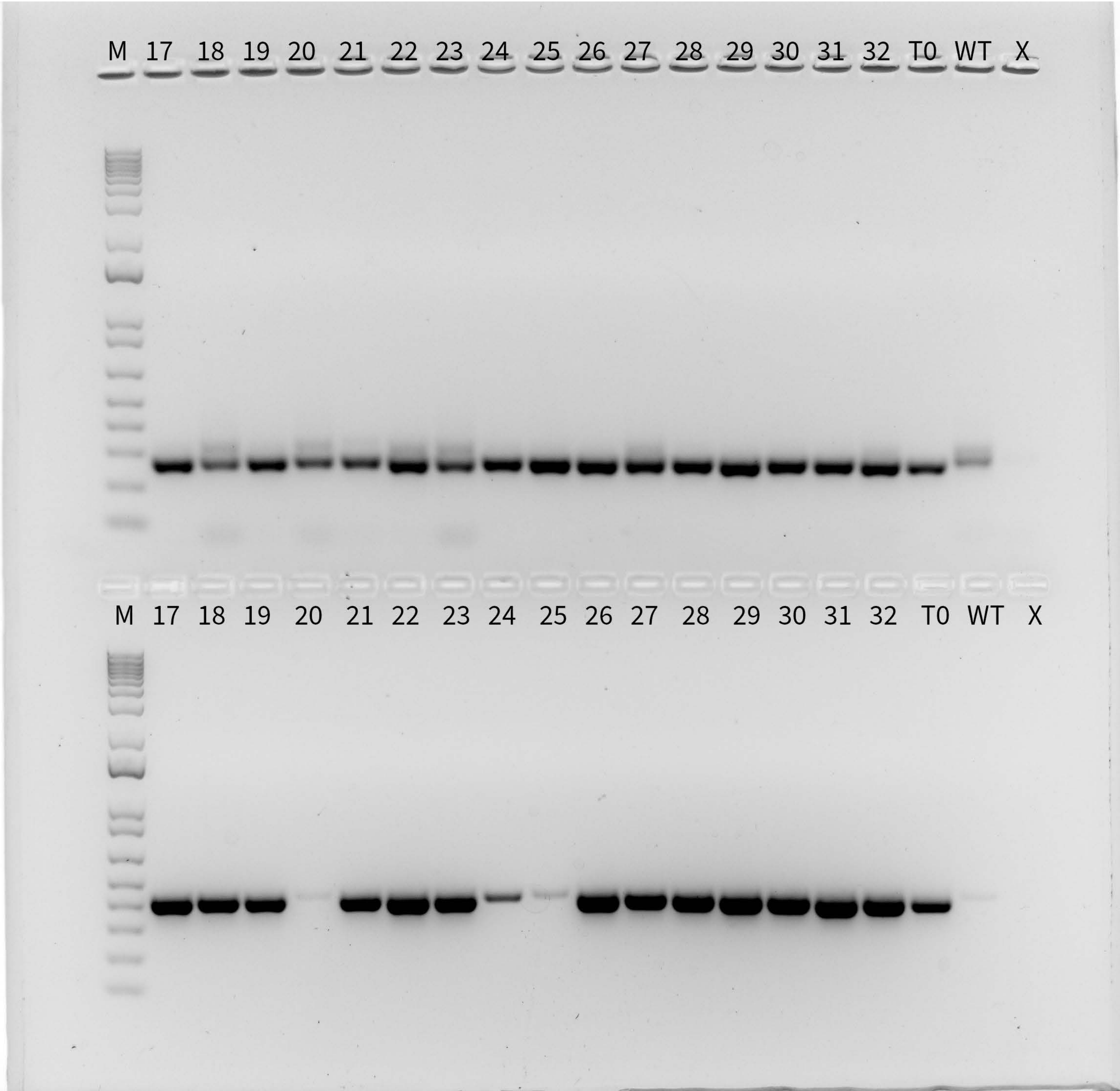

A 1 Kb Plus DNA Ladder (Invitrogen™, Thermo Fisher Scientific) was loaded in lane M and used as the molecular weight standard for estimation of PCR amplicon sizes. Gel images were acquired using an Invitrogen iBright™ 1500 Imaging System under standard imaging conditions. This original gel corresponds to Figure 2, panel F of the manuscript. Lane identities are indicated above the gel, and any lanes not included in the final figure are marked with an "X" above the corresponding lane label. All annotations were added without obscuring any bands, background signal, or other image features

Source for Figs 3 and 4. Panel C.

Fig 4. Panel C

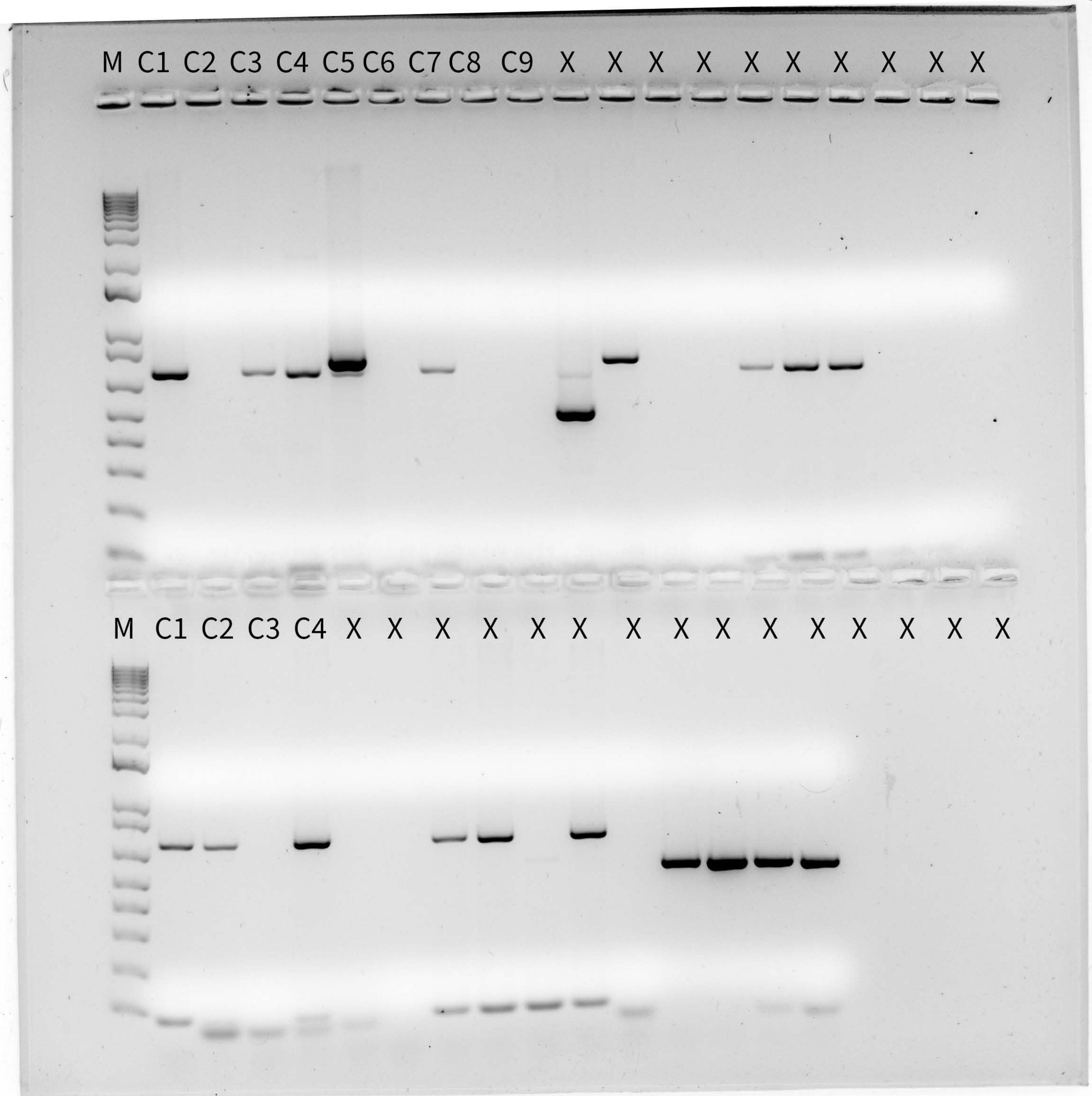

Fig 3. Panel C

A 1 Kb Plus DNA Ladder (Invitrogen™, Thermo Fisher Scientific) was loaded in lane M and used as the molecular weight standard for estimation of PCR amplicon sizes. Gel images were acquired using an Invitrogen iBright™ 1500 Imaging System under standard imaging conditions. This original gel corresponds to Figures 3 and 4, panel C of the manuscript. Lane identities are indicated above the gel, and any lanes not included in the final figure are marked with an "X" above the corresponding lane label. All annotations were added without obscuring any bands, background signal, or other image features

Source for Fig 4. Panel D

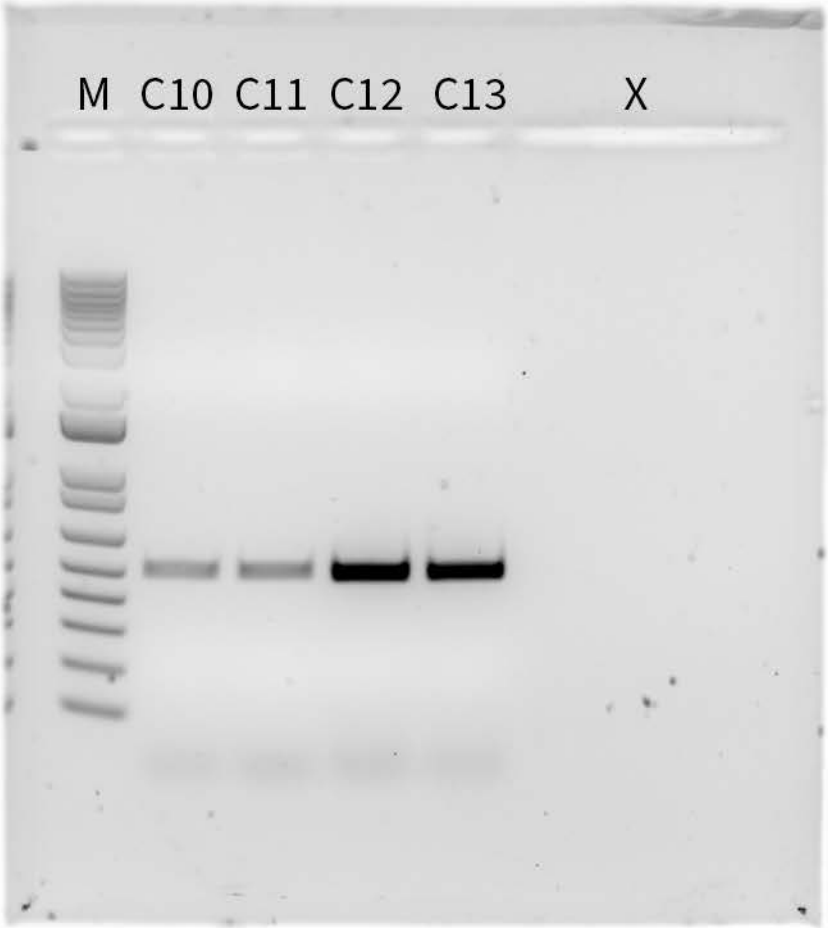

A 1 Kb Plus DNA Ladder (Invitrogen™, Thermo Fisher Scientific) was loaded in lane M and used as the molecular weight standard for estimation of PCR amplicon sizes. Gel images were acquired using an Invitrogen iBright™ 1500 Imaging System under standard imaging conditions. This original gel corresponds to Figure 4, panel D of the manuscript. Lane identities are indicated above the gel, and any lanes not included in the final figure are marked with an "X" above the corresponding lane label. All annotations were added without obscuring any bands, background signal, or other image features

Source for Fig 5. Gels B and C. Samples 1-25

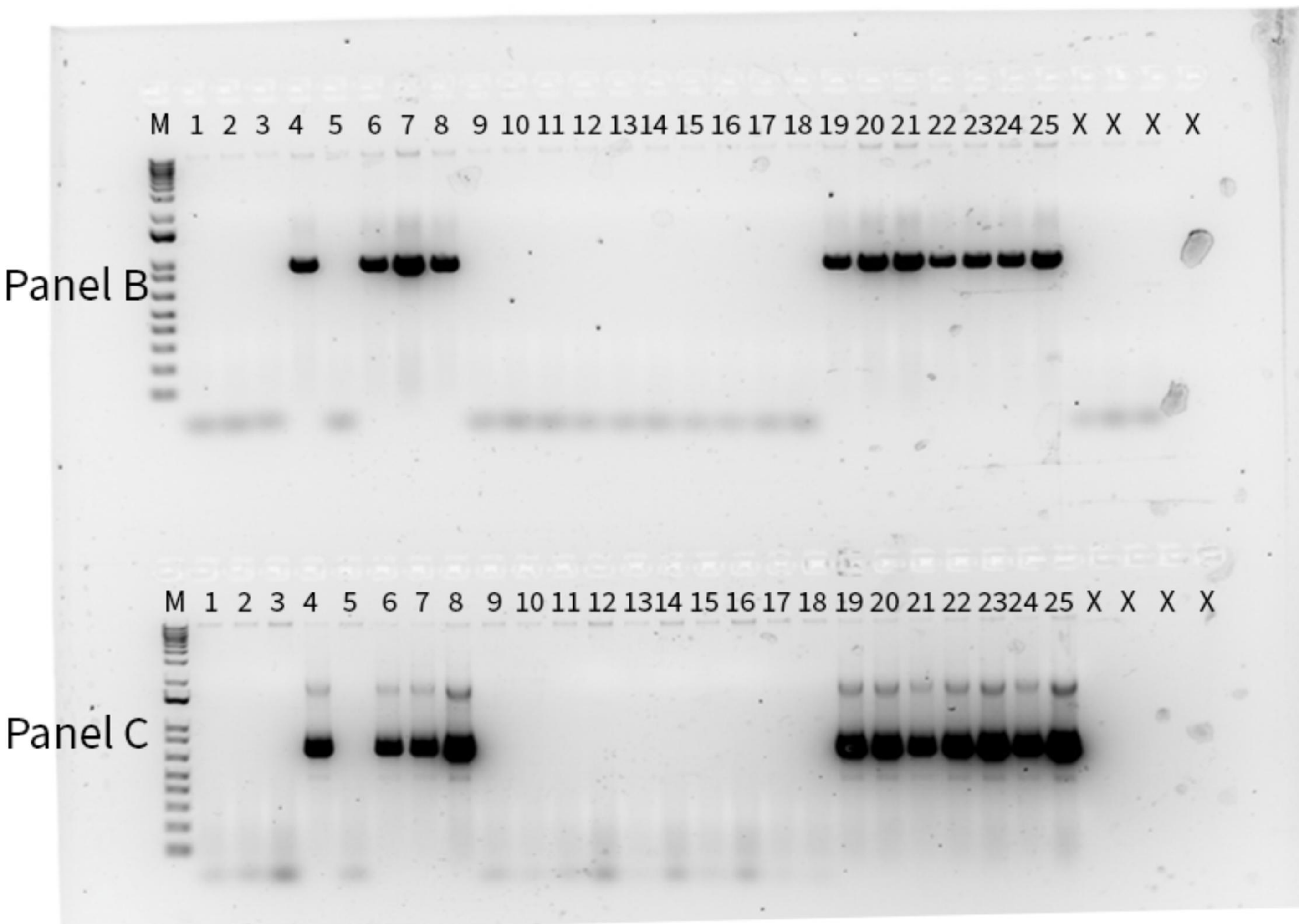

A 1 Kb Plus DNA Ladder (Invitrogen™, Thermo Fisher Scientific) was loaded in lane M and used as the molecular weight standard for estimation of PCR amplicon sizes. Gel images were acquired using an Invitrogen iBright™ 1500 Imaging System under standard imaging conditions. This original gel corresponds to Figure 5, panel B of the manuscript. Lane identities are indicated above the gel, and any lanes not included in the final figure are marked with an “X” above the corresponding lane label. All annotations were added without obscuring any bands, background signal, or other image features

Source for Fig 5. Panel B and C

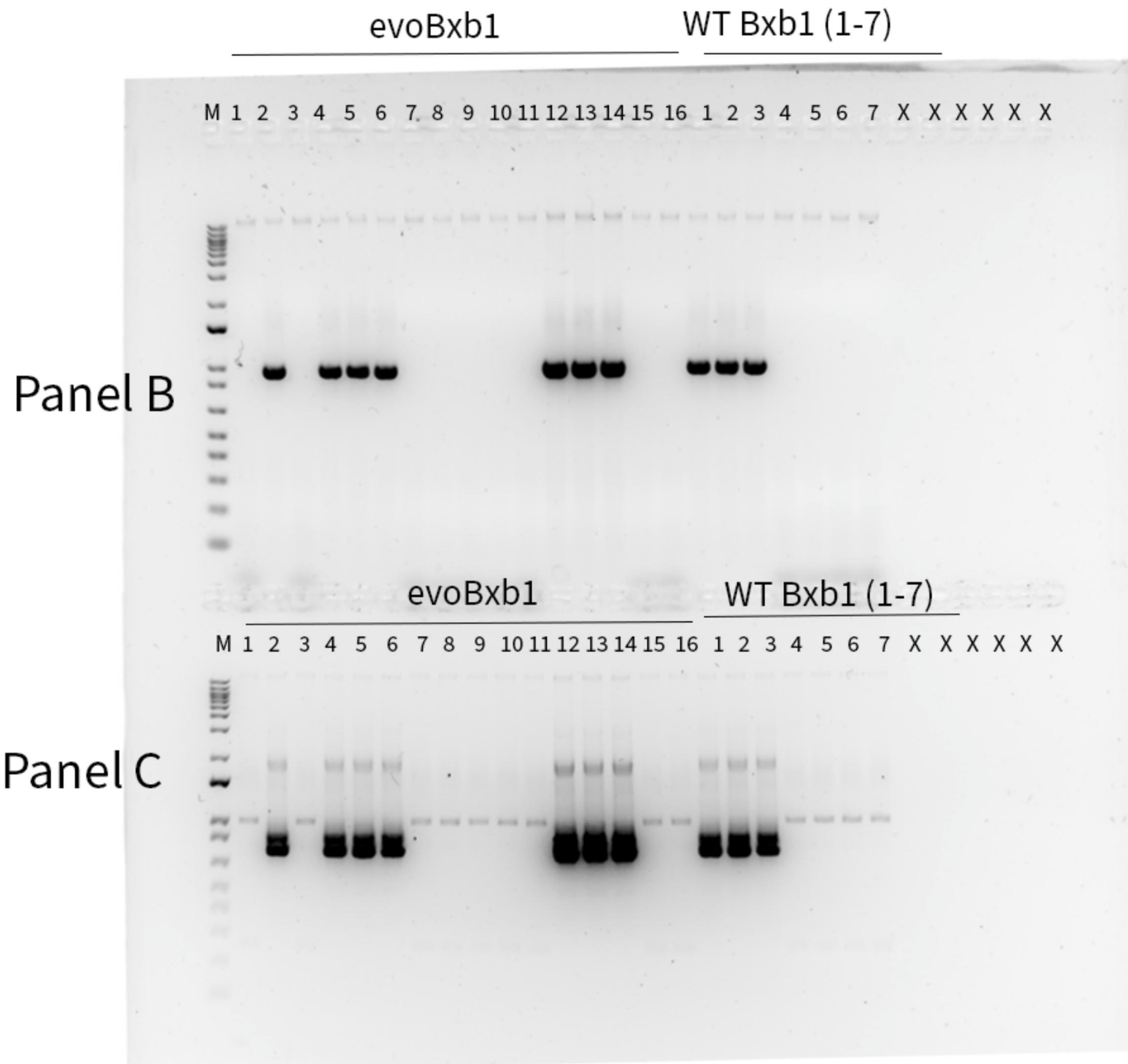

A 1 Kb Plus DNA Ladder (Invitrogen™, Thermo Fisher Scientific) was loaded in lane M and used as the molecular weight standard for estimation of PCR amplicon sizes. Gel images were acquired using an Invitrogen iBright™ 1500 Imaging System under standard imaging conditions. This original gel corresponds to Figure 5, panel B of the manuscript. Lane identities are indicated above the gel, and any lanes not included in the final figure are marked with an "X" above the corresponding lane label. All annotations were added without obscuring any bands, background signal, or other image features

Source for S5 Fig. Panel A

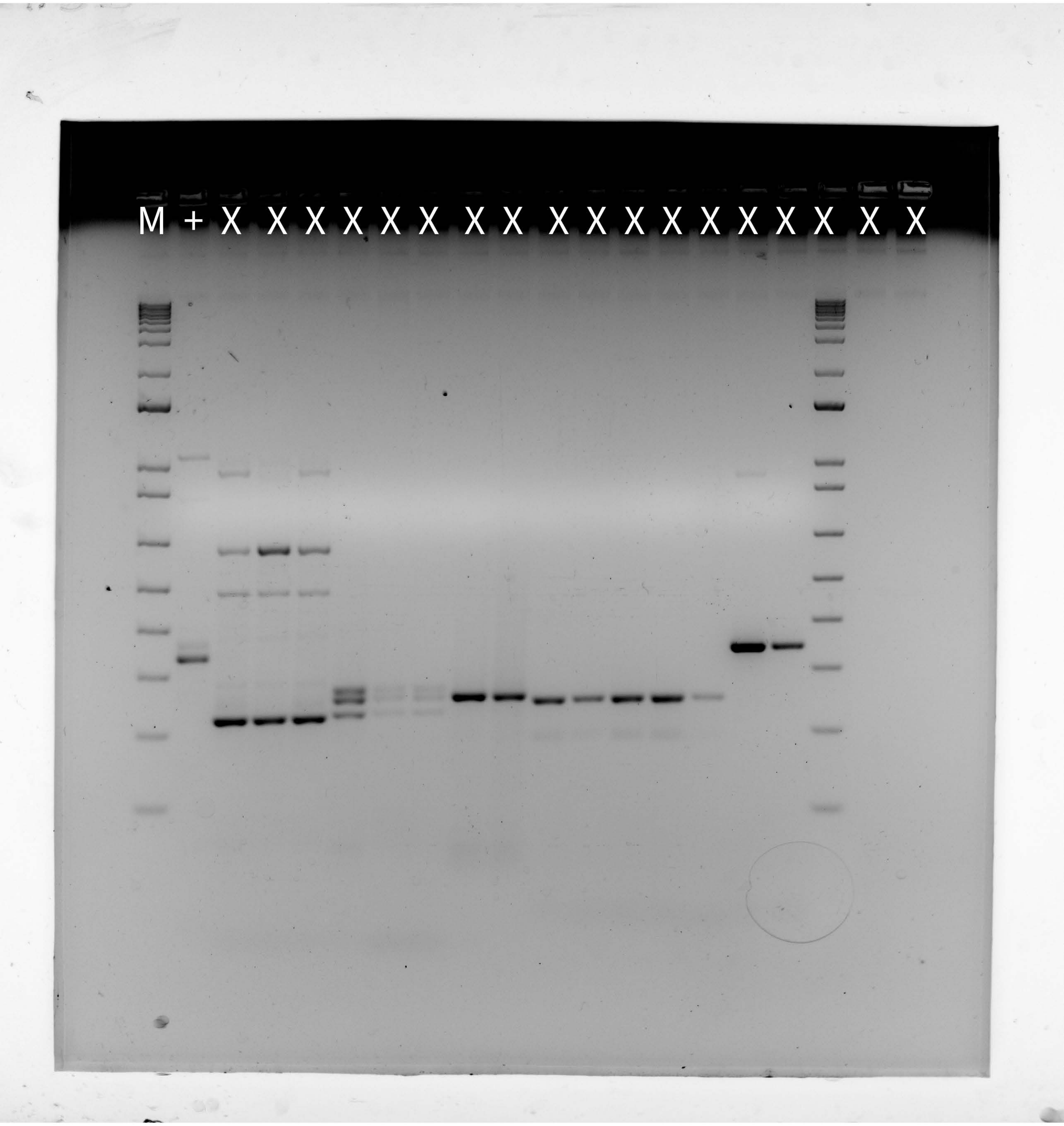

A 1 Kb Plus DNA Ladder (Invitrogen™, Thermo Fisher Scientific) was loaded in lane M and used as the molecular weight standard for estimation of PCR amplicon sizes. Gel images were acquired using an Invitrogen iBright™ 1500 Imaging System under standard imaging conditions. This original gel corresponds to S5 Fig., panel A. Lane identities are indicated above the gel, and any lanes not included in the final figure are marked with an "X" above the corresponding lane label. All annotations were added without obscuring any bands, background signal, or other image features

Source for S5 Fig. Panel A. (HincII digestion)

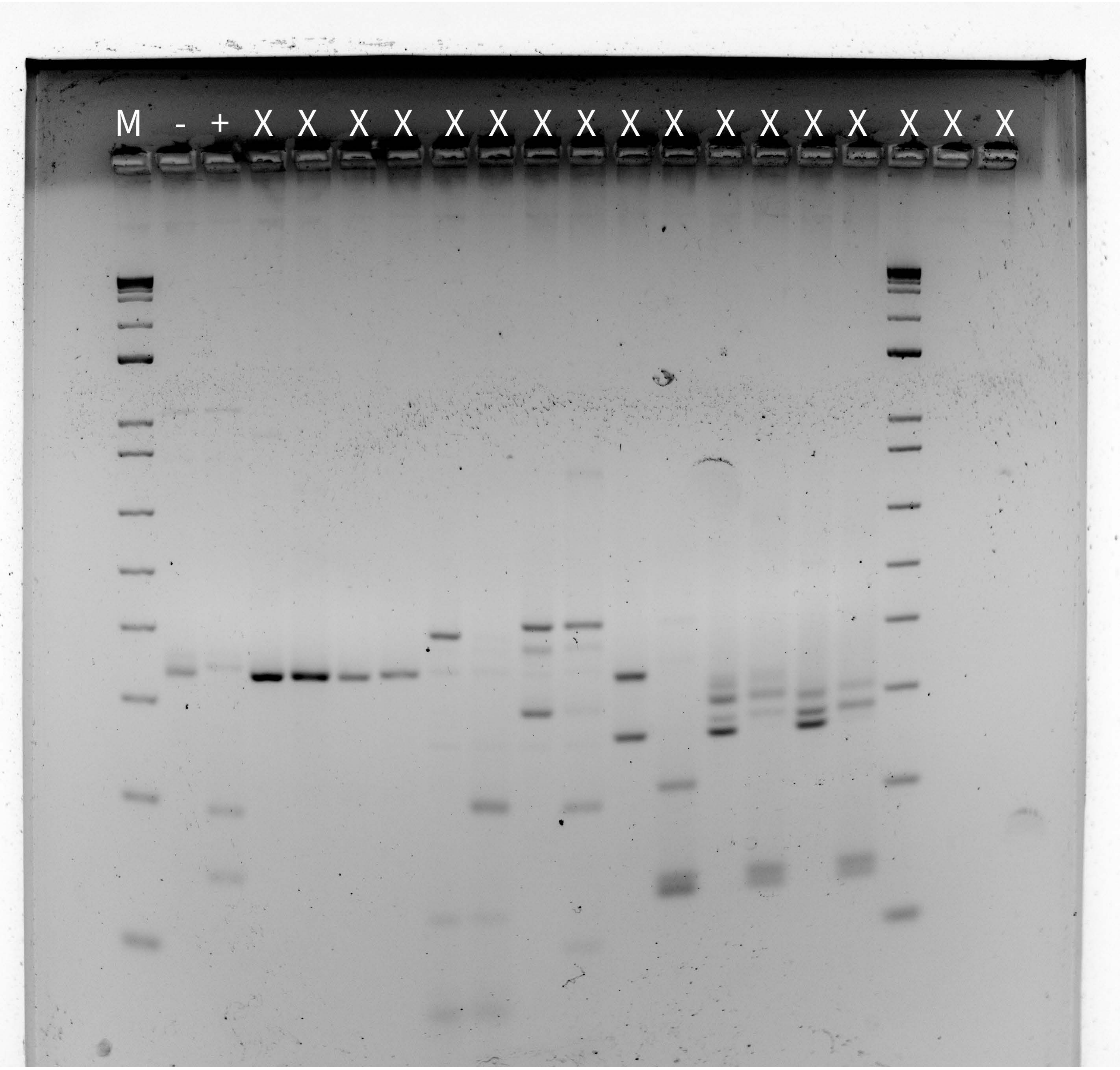

A 1 Kb Plus DNA Ladder (Invitrogen™, Thermo Fisher Scientific) was loaded in lane M and used as the molecular weight standard for estimation of PCR amplicon sizes. Gel images were acquired using an Invitrogen iBright™ 1500 Imaging System under standard imaging conditions. This original gel corresponds to S5 Fig., panel A. Lane identities are indicated above the gel, and any lanes not included in the final figure are marked with an "X" above the corresponding lane label. All annotations were added without obscuring any bands, background signal, or other image features

Source for S5 Fig. Panel D. Samples 1-16

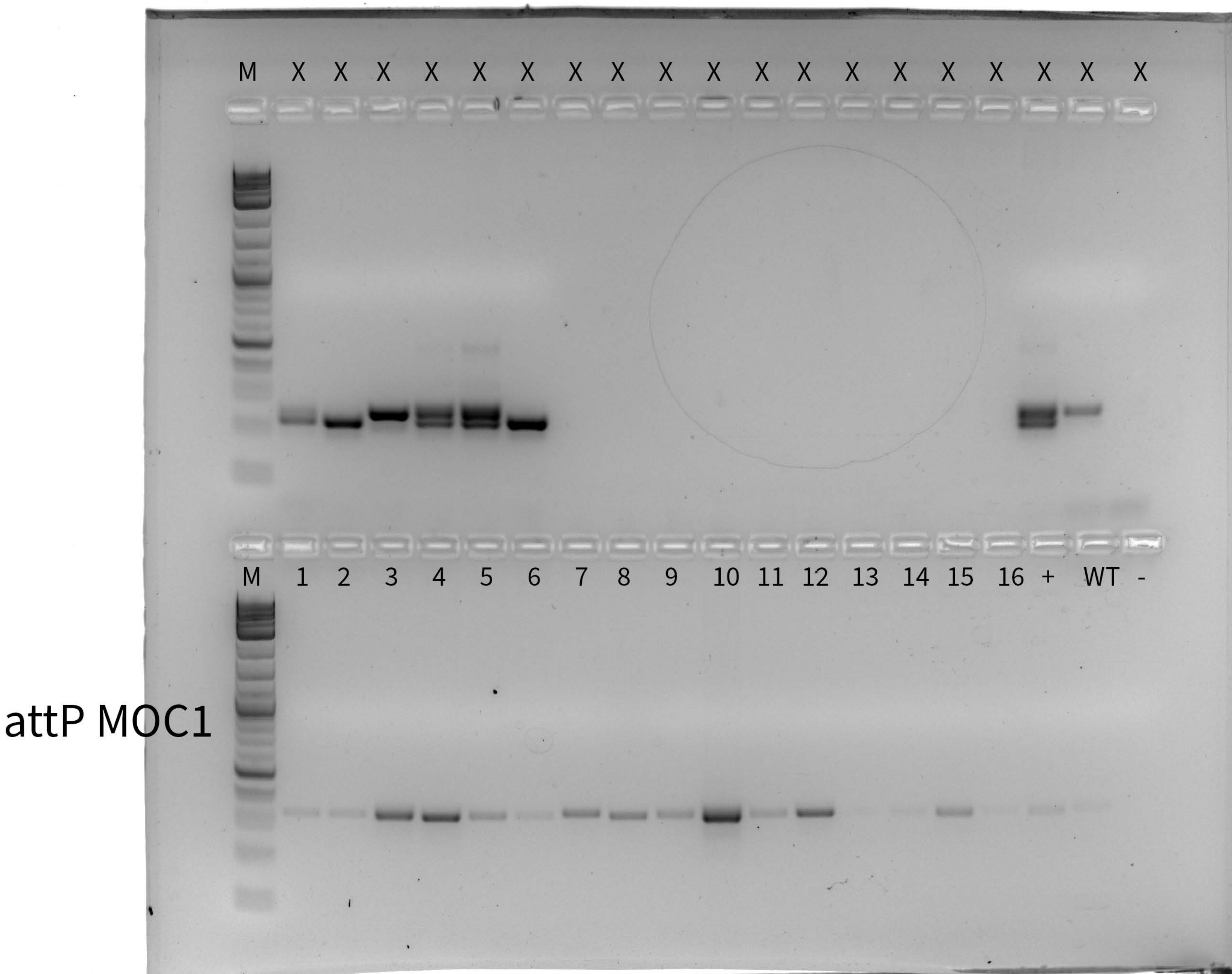

A 2-Log DNA Ladder (New England BioLabs; 0.1–10.0 kb) was loaded in lane M and used as the molecular weight standard for estimation of PCR amplicon sizes. Gel images were acquired using an Invitrogen iBright™ 1500 Imaging System under standard imaging conditions. This original gel corresponds to S5 Fig., panel D. Lane identities are indicated above the gel, and any lanes not included in the final figure are marked with an "X" above the corresponding lane label. All annotations were added without obscuring any bands, background signal, or other image features.

Source for S5 Fig. Panel D. Samples 17-38. attP MOC1

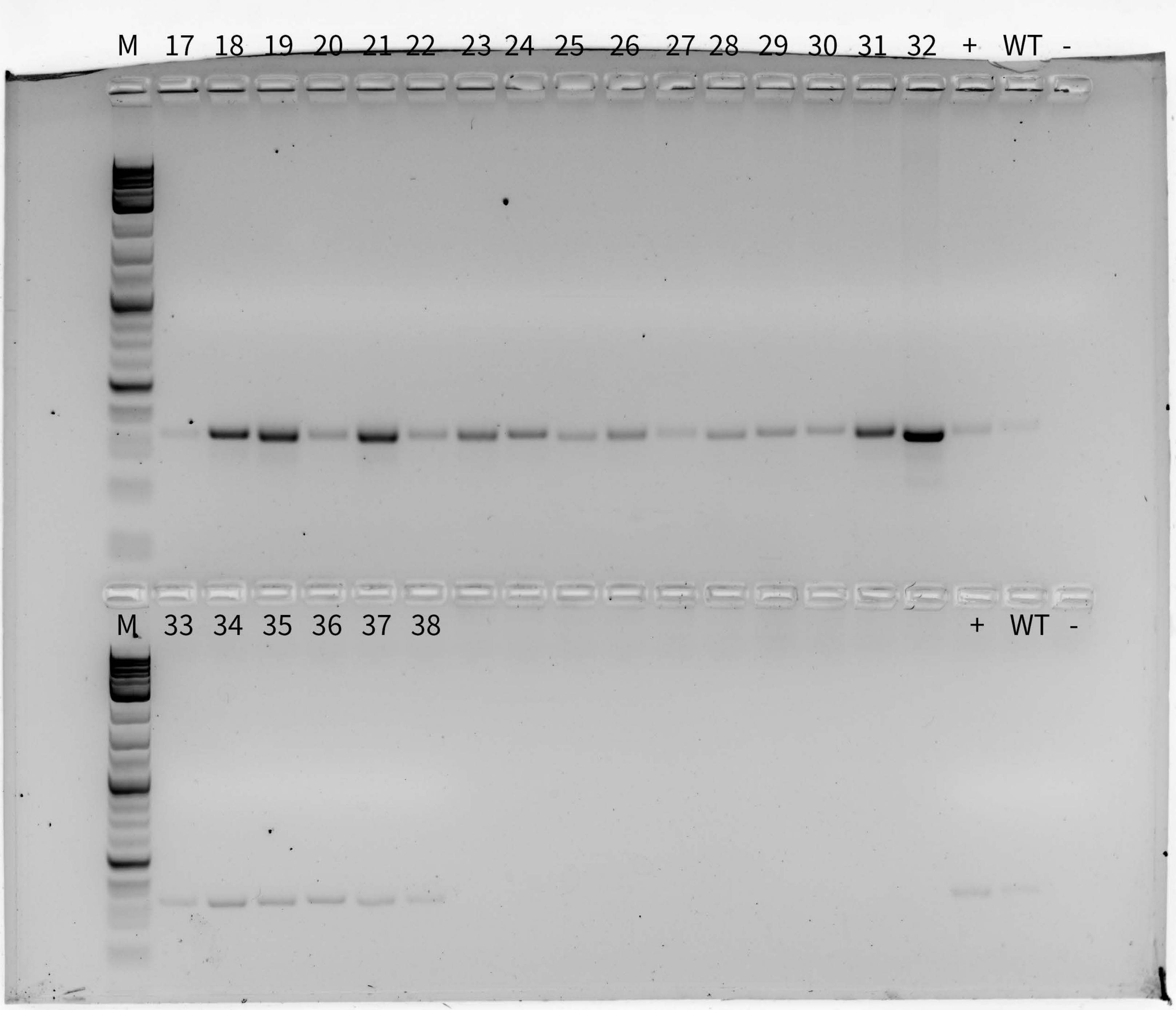

A 2-Log DNA Ladder (New England BioLabs; 0.1–10.0 kb) was loaded in lane M and used as the molecular weight standard for estimation of PCR amplicon sizes. Gel images were acquired using an Invitrogen iBright™ 1500 Imaging System under standard imaging conditions. This original gel corresponds to S5 Fig., panel D. Lane identities are indicated above the gel, and any lanes not included in the final figure are marked with an "X" above the corresponding lane label. All annotations were added without obscuring any bands, background signal, or other image features.

Source for S5 Fig. Panel D. Samples 1-38. T-DNA

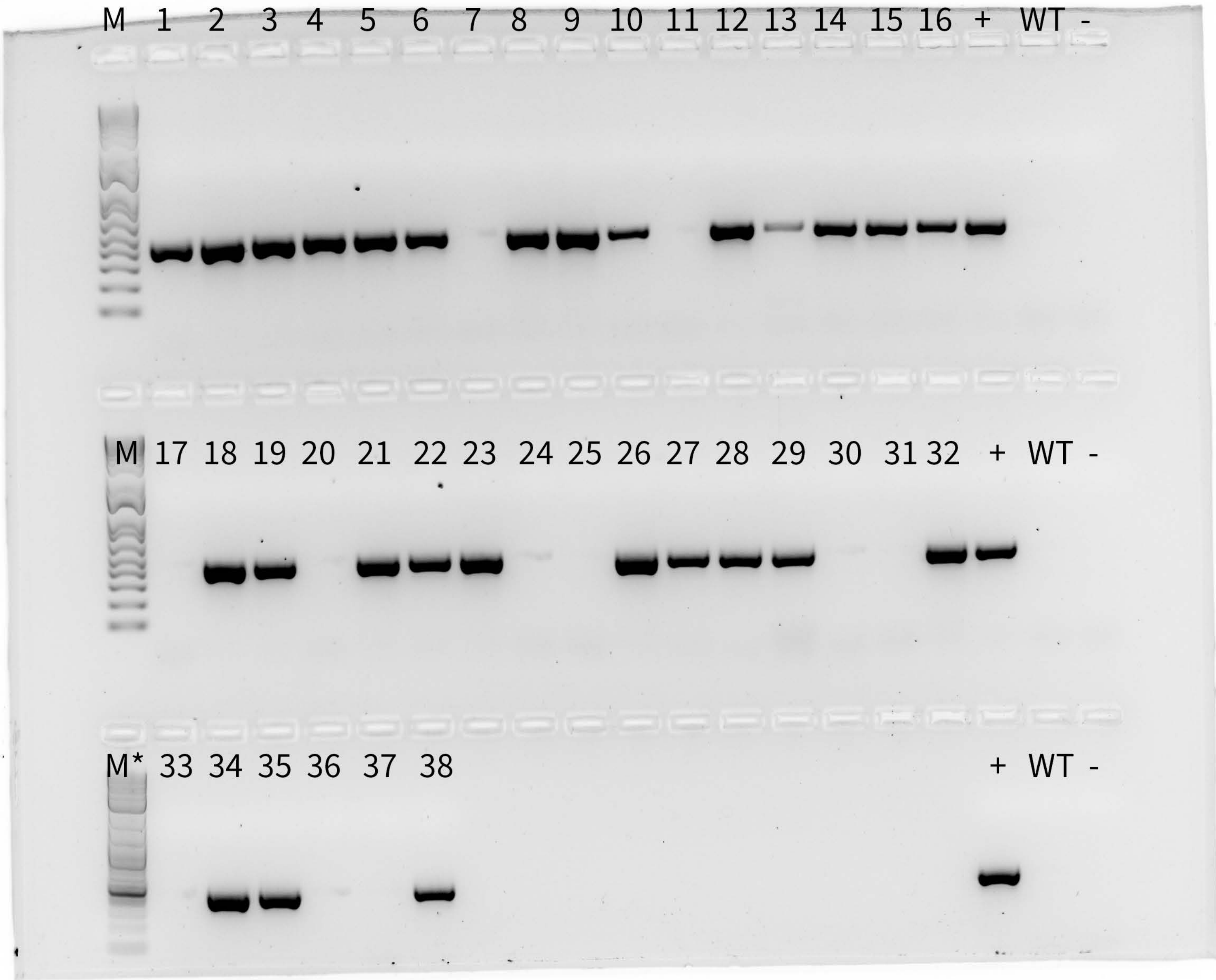

A 1 Kb Plus DNA Ladder (Invitrogen™, Thermo Fisher Scientific) (M) or 2-Log DNA Ladder (New England BioLabs; 0.1–10.0 kb) (M*) was loaded in lane M and used as the molecular weight standard for estimation of PCR amplicon sizes. Gel images were acquired using an Invitrogen iBright™ 1500 Imaging System under standard imaging conditions. This original gel corresponds to S5 Fig., panel D. Lane identities are indicated above the gel, and any lanes not included in the final figure are marked with an “X” above the corresponding lane label. All annotations were added without obscuring any bands, background signal, or other image features.

Source for S7 Fig. Panel A and S8 Fig. Panel B

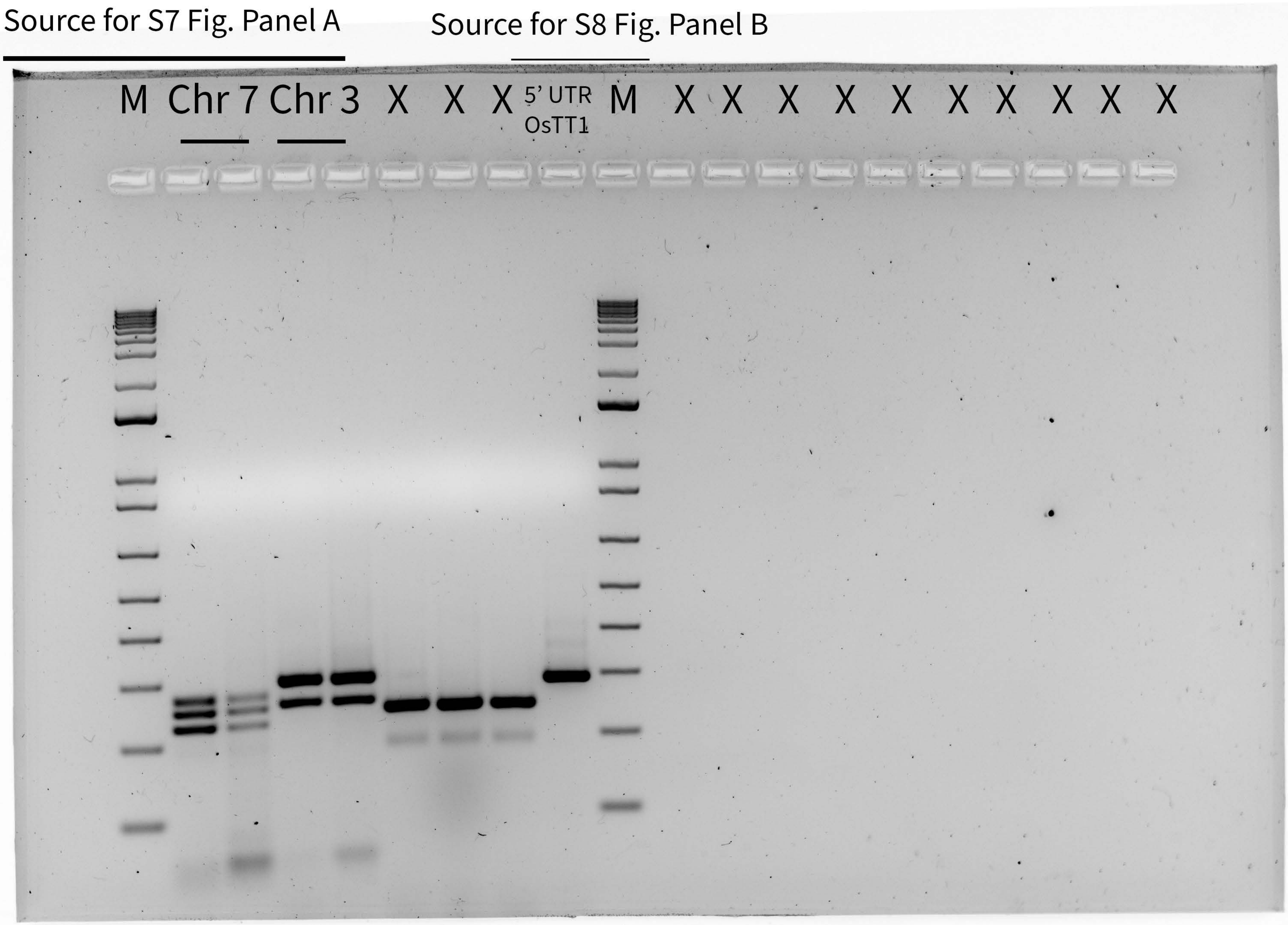

A 1 Kb Plus DNA Ladder (Invitrogen™, Thermo Fisher Scientific) was loaded in lane M and used as the molecular weight standard for estimation of PCR amplicon sizes. Gel images were acquired using an Invitrogen iBright™ 1500 Imaging System under standard imaging conditions. This original gel corresponds to S7 Fig., panel A. Lane identities are indicated above the gel, and any lanes not included in the final figure are marked with an "X" above the corresponding lane label. All annotations were added without obscuring any bands, background signal, or other image features

Source for S10 Fig. Panel A. Samples 1-23

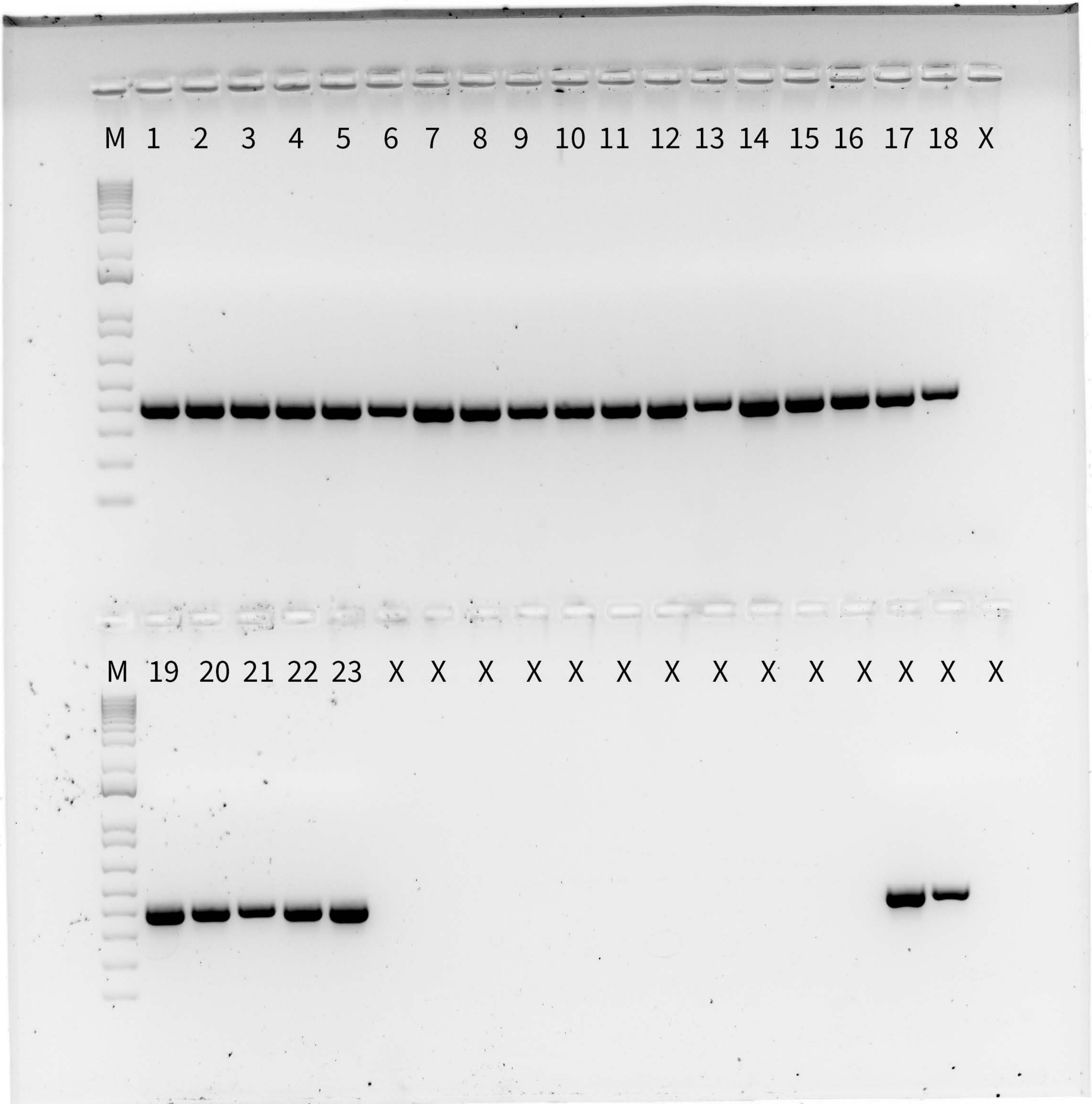

A 1 Kb Plus DNA Ladder (Invitrogen™, Thermo Fisher Scientific) was loaded in lane M and used as the molecular weight standard for estimation of PCR amplicon sizes. Gel images were acquired using an Invitrogen iBright™ 1500 Imaging System under standard imaging conditions. This original gel corresponds to S10 Fig., panel A. Lane identities are indicated above the gel, and any lanes not included in the final figure are marked with an "X" above the corresponding lane label. All annotations were added without obscuring any bands, background signal, or other image features

Source for S10 Fig. Panel A. Samples 24-38

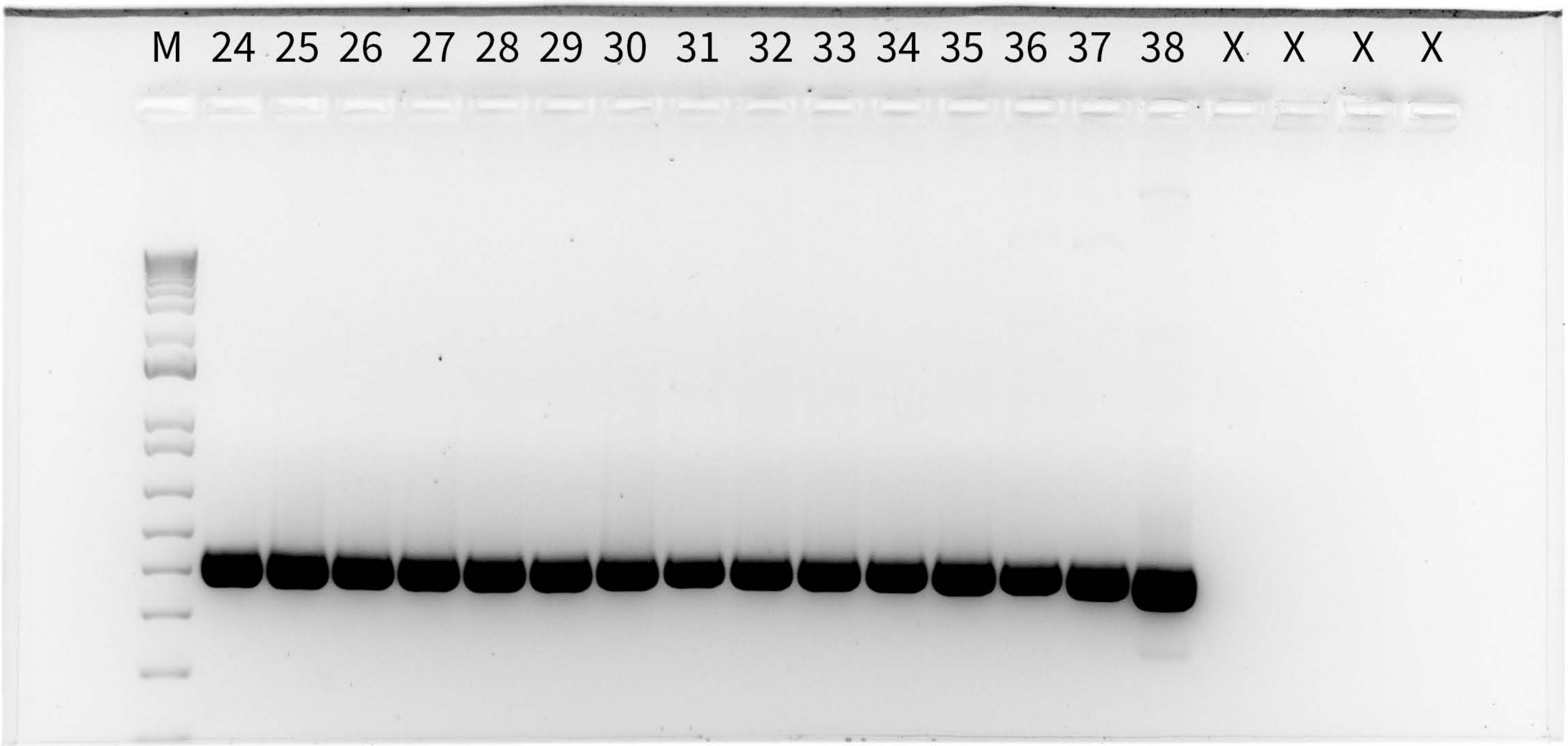

A 1 Kb Plus DNA Ladder (Invitrogen™, Thermo Fisher Scientific) was loaded in lane M and used as the molecular weight standard for estimation of PCR amplicon sizes. Gel images were acquired using an Invitrogen iBright™ 1500 Imaging System under standard imaging conditions. This original gel corresponds to S10 Fig., panel A. Lane identities are indicated above the gel, and any lanes not included in the final figure are marked with an “X” above the corresponding lane label. All annotations were added without obscuring any bands, background signal, or other image features

Source for S10 Fig. Panel B. Samples 1-16. attP GSH Chr3

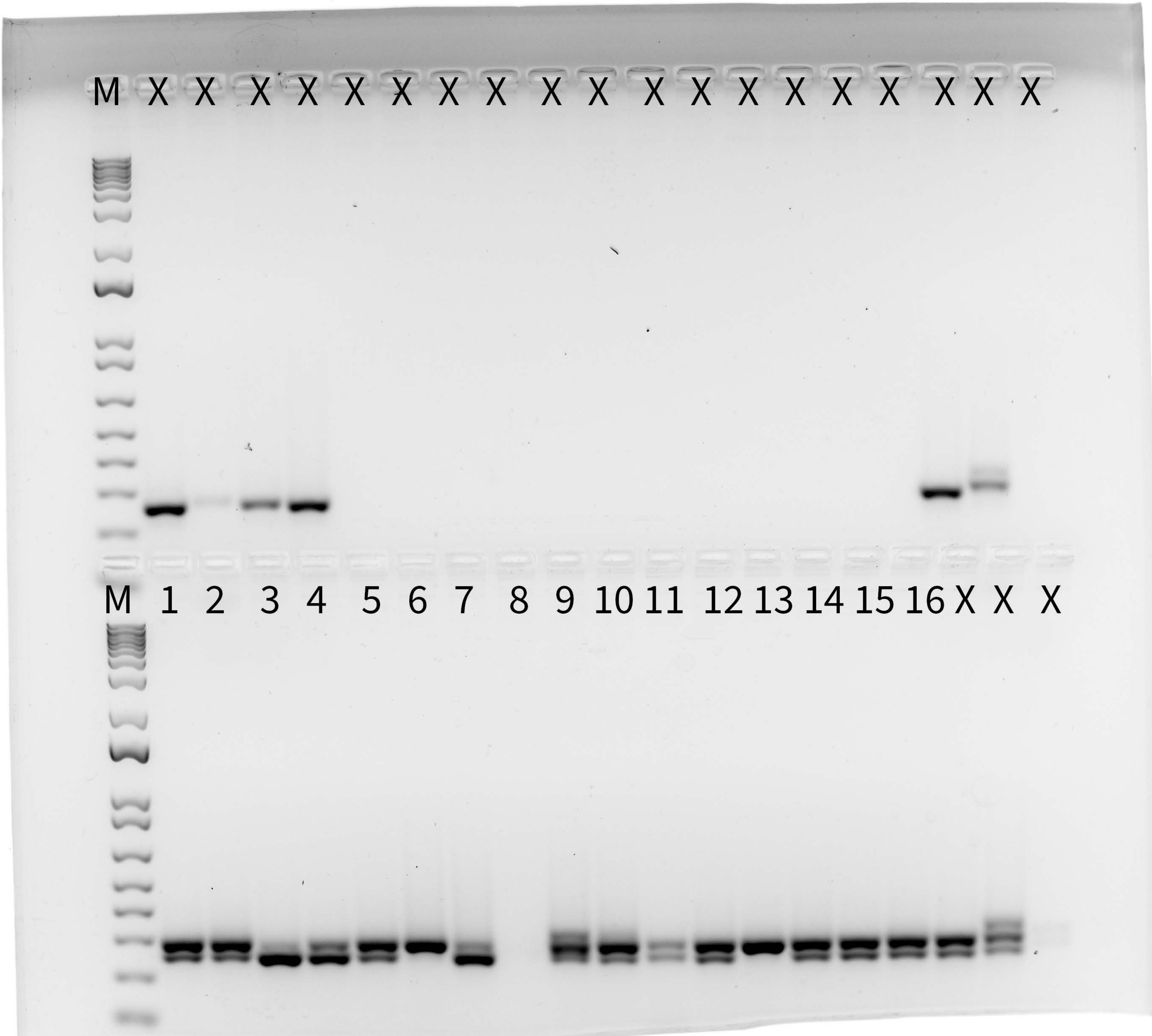

A 1 Kb Plus DNA Ladder (Invitrogen™, Thermo Fisher Scientific) was loaded in lane M and used as the molecular weight standard for estimation of PCR amplicon sizes. Gel images were acquired using an Invitrogen iBright™ 1500 Imaging System under standard imaging conditions. This original gel corresponds to S10 Fig., panel B. Lane identities are indicated above the gel, and any lanes not included in the final figure are marked with an "X" above the corresponding lane label. All annotations were added without obscuring any bands, background signal, or other image features

Source for S10 Fig. Panel B. Samples 17-42. attP GSH Ch3

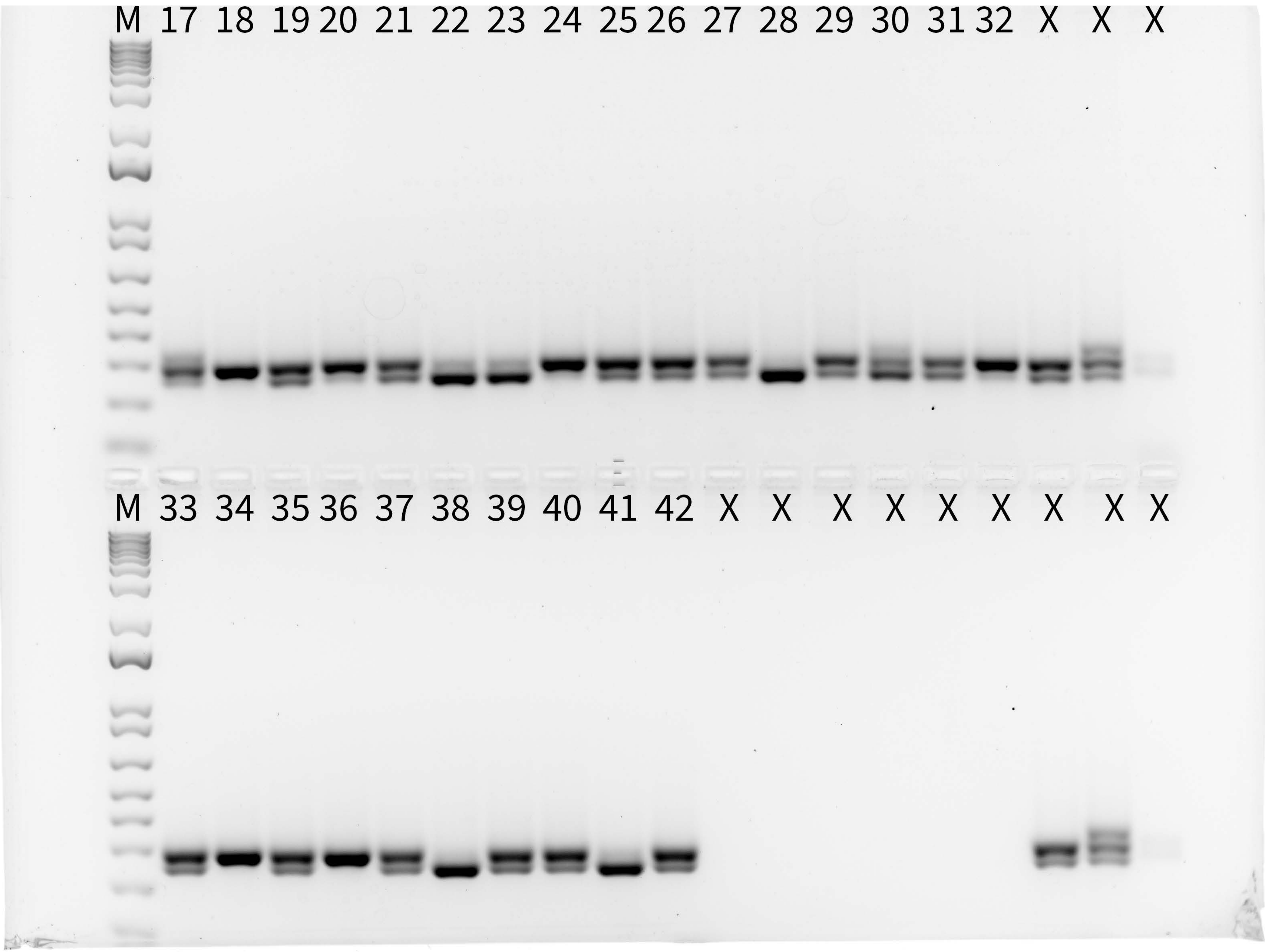

A 1 Kb Plus DNA Ladder (Invitrogen™, Thermo Fisher Scientific) was loaded in lane M and used as the molecular weight standard for estimation of PCR amplicon sizes. Gel images were acquired using an Invitrogen iBright™ 1500 Imaging System under standard imaging conditions. This original gel corresponds to S10 Fig., panel B. Lane identities are indicated above the gel, and any lanes not included in the final figure are marked with an "X" above the corresponding lane label. All annotations were added without obscuring any bands, background signal, or other image features

Source for S10 Fig. Panel B. Samples 1-32. T-DNA

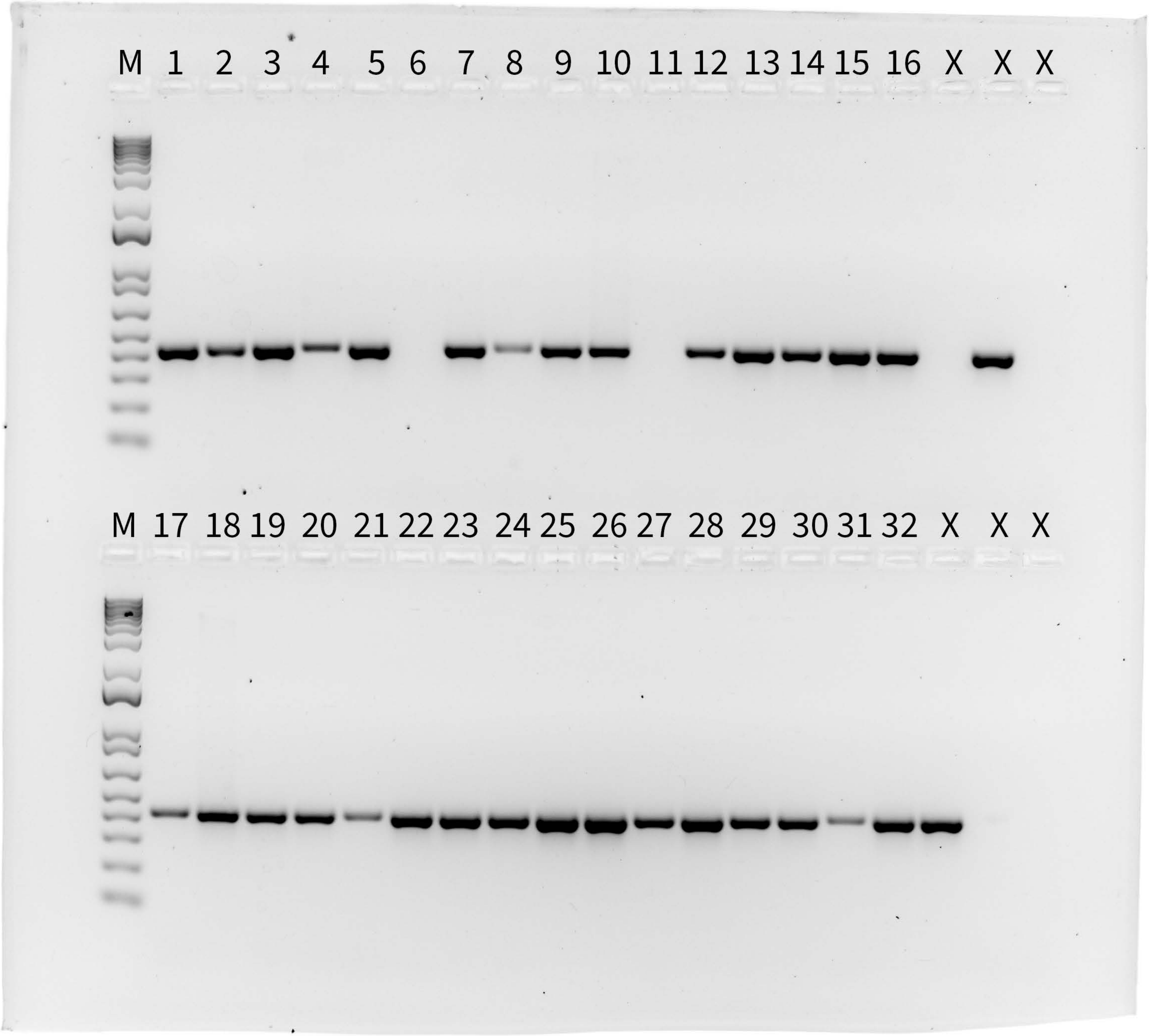

A 1 Kb Plus DNA Ladder (Invitrogen™, Thermo Fisher Scientific) was loaded in lane M and used as the molecular weight standard for estimation of PCR amplicon sizes. Gel images were acquired using an Invitrogen iBright™ 1500 Imaging System under standard imaging conditions. This original gel corresponds to S10 Fig., panel B. Lane identities are indicated above the gel, and any lanes not included in the final figure are marked with an "X" above the corresponding lane label. All annotations were added without obscuring any bands, background signal, or other image features

Source for S10 Fig. Panel B. Samples 33-42. T-DNA

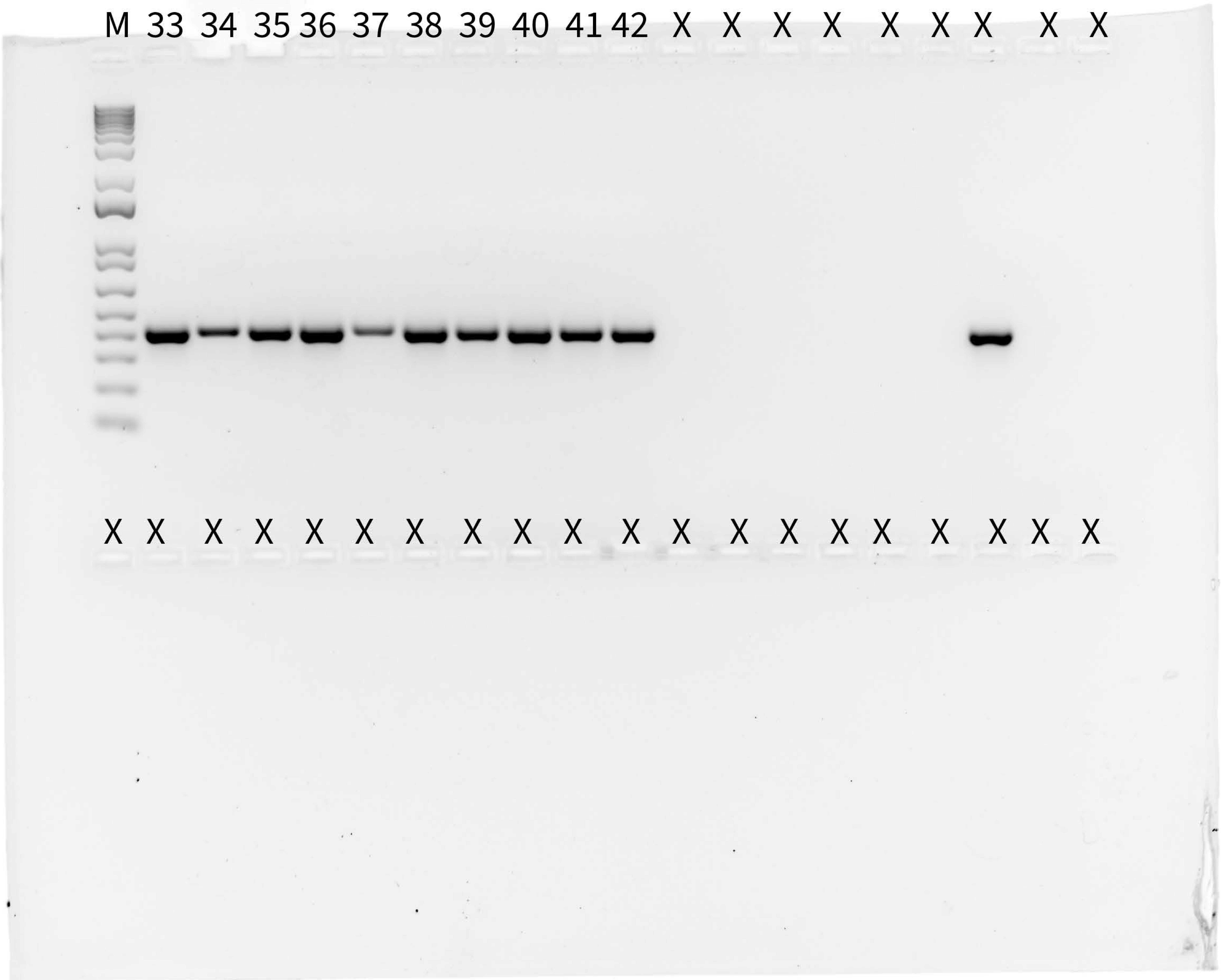

A 1 Kb Plus DNA Ladder (Invitrogen™, Thermo Fisher Scientific) was loaded in lane M and used as the molecular weight standard for estimation of PCR amplicon sizes. Gel images were acquired using an Invitrogen iBright™ 1500 Imaging System under standard imaging conditions. This original gel corresponds to S10 Fig., panel B. Lane identities are indicated above the gel, and any lanes not included in the final figure are marked with an "X" above the corresponding lane label. All annotations were added without obscuring any bands, background signal, or other image features

Source for S10 Fig. Panel C. Samples 1-32. attP GSH Chr 7

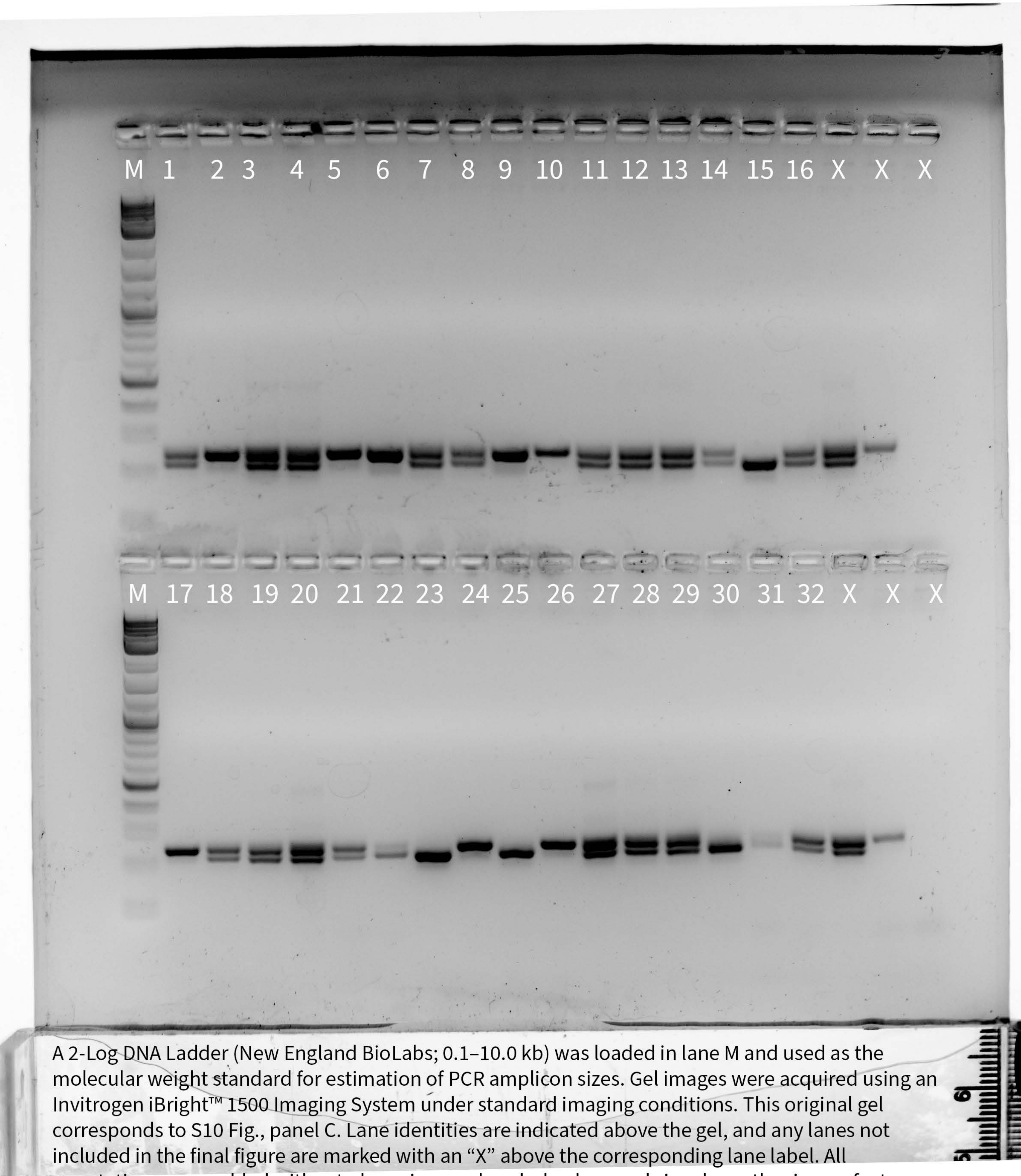

A 2-Log DNA Ladder (New England BioLabs; 0.1–10.0 kb) was loaded in lane M and used as the molecular weight standard for estimation of PCR amplicon sizes. Gel images were acquired using an Invitrogen iBright™ 1500 Imaging System under standard imaging conditions. This original gel corresponds to S10 Fig., panel C. Lane identities are indicated above the gel, and any lanes not included in the final figure are marked with an "X" above the corresponding lane label. All annotations were added without obscuring any bands, background signal, or other image features

Source for S10 Fig. Panel C. Samples 33-38. attP GSH Chr 7

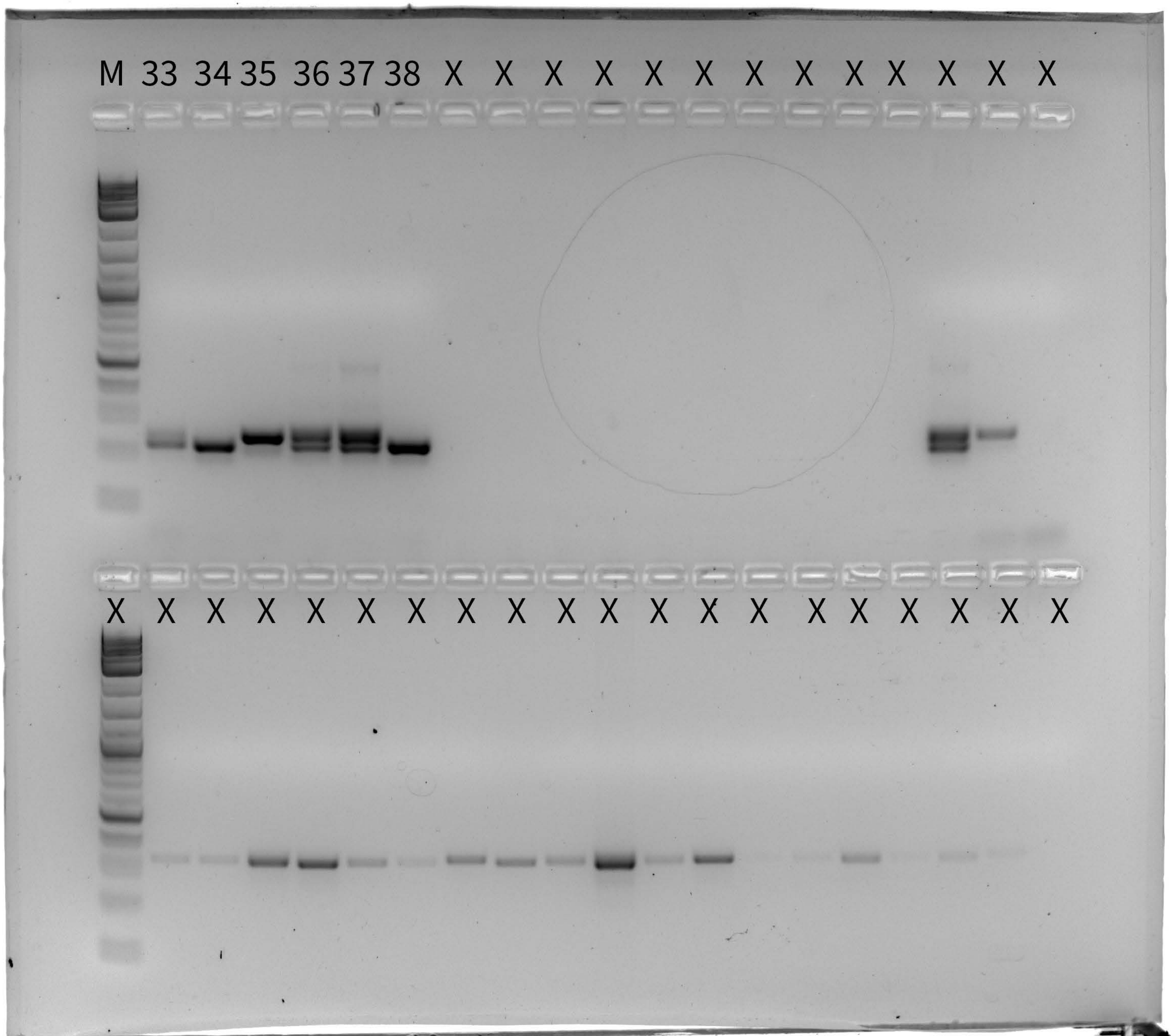

A 2-Log DNA Ladder (New England BioLabs; 0.1–10.0 kb) was loaded in lane M and used as the molecular weight standard for estimation of PCR amplicon sizes. Gel images were acquired using an Invitrogen iBright™ 1500 Imaging System under standard imaging conditions. This original gel corresponds to S10 Fig., panel C. Lane identities are indicated above the gel, and any lanes not included in the final figure are marked with an “X” above the corresponding lane label. All annotations were added without obscuring any bands, background signal, or other image features

Source for S10 Fig. Panel C. Samples 1-38. T-DNA

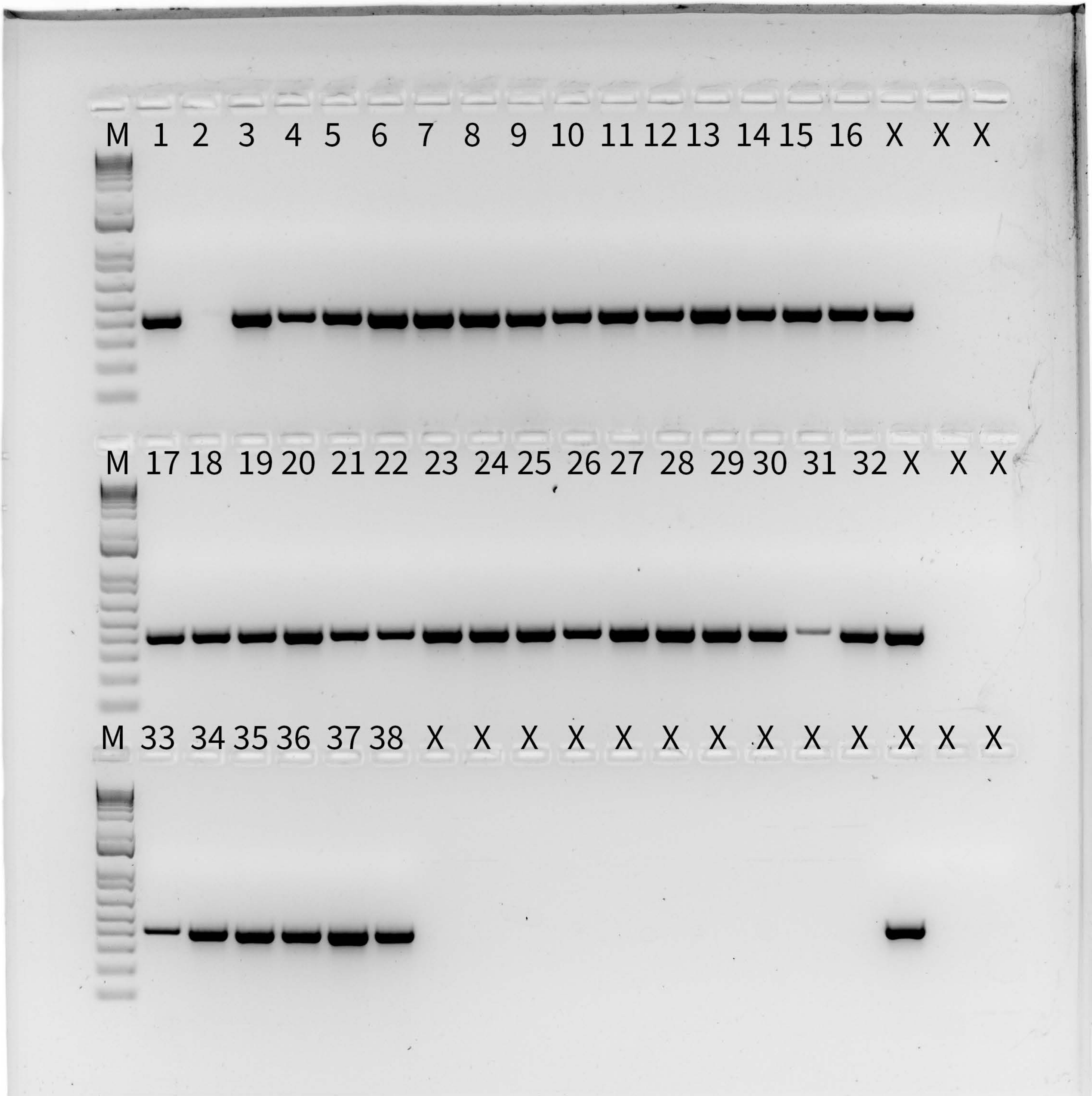

A 1 Kb Plus DNA Ladder (Invitrogen™, Thermo Fisher Scientific) was loaded in lane M and used as the molecular weight standard for estimation of PCR amplicon sizes. Gel images were acquired using an Invitrogen iBright™ 1500 Imaging System under standard imaging conditions. This original gel corresponds to S10 Fig., panel C. Lane identities are indicated above the gel, and any lanes not included in the final figure are marked with an "X" above the corresponding lane label. All annotations were added without obscuring any bands, background signal, or other image features

Source for S10 Fig. Panel C. Samples 39-70. attP GSH Chr 7

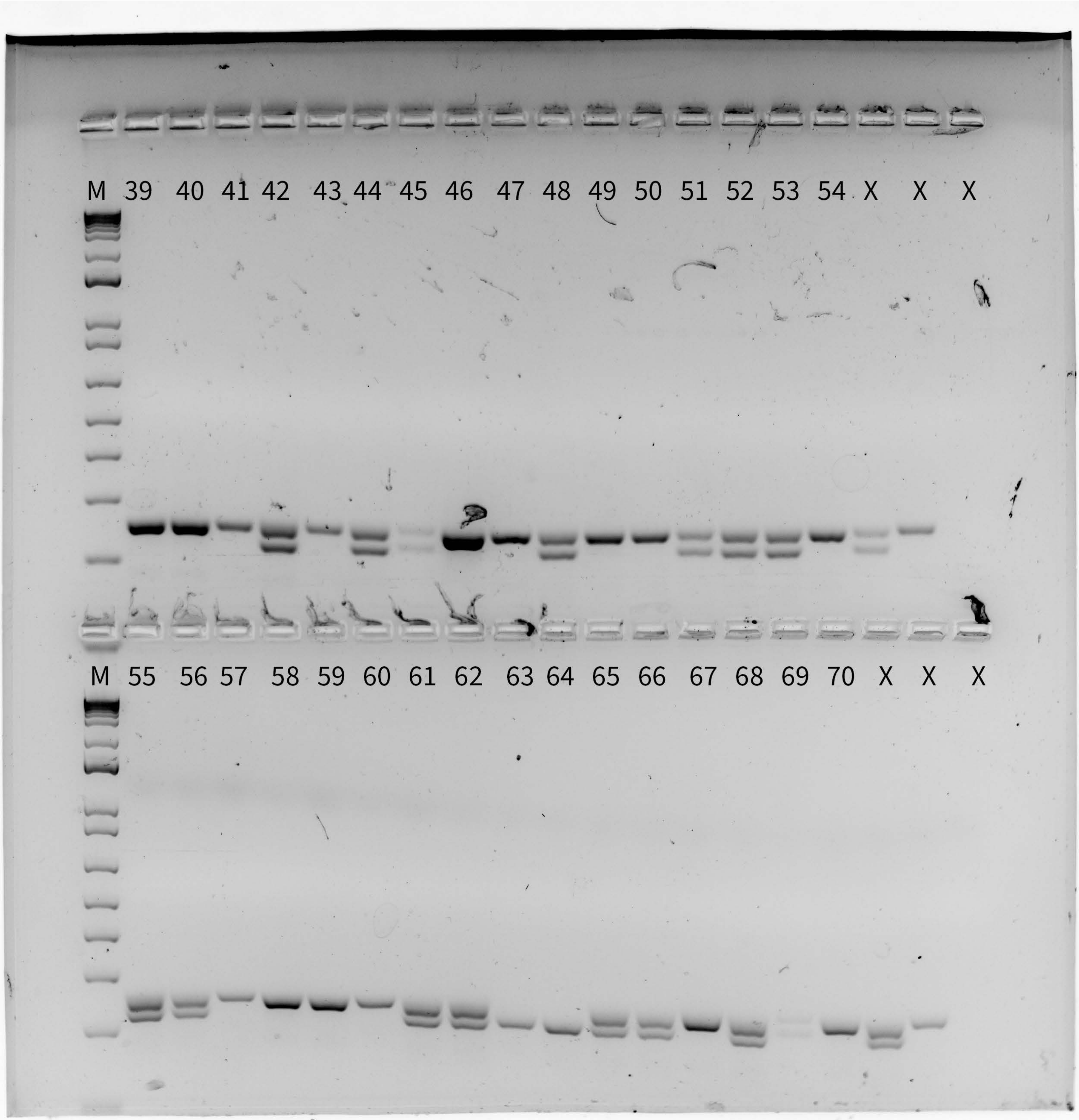

A 1 Kb Plus DNA Ladder (Invitrogen™, Thermo Fisher Scientific) was loaded in lane M and used as the molecular weight standard for estimation of PCR amplicon sizes. Gel images were acquired using an Invitrogen iBright™ 1500 Imaging System under standard imaging conditions. This original gel corresponds to S10 Fig., panel C. Lane identities are indicated above the gel, and any lanes not included in the final figure are marked with an "X" above the corresponding lane label. All annotations were added without obscuring any bands, background signal, or other image features

Source for S10 Fig. Panel C. Samples 39-70. T-DNA

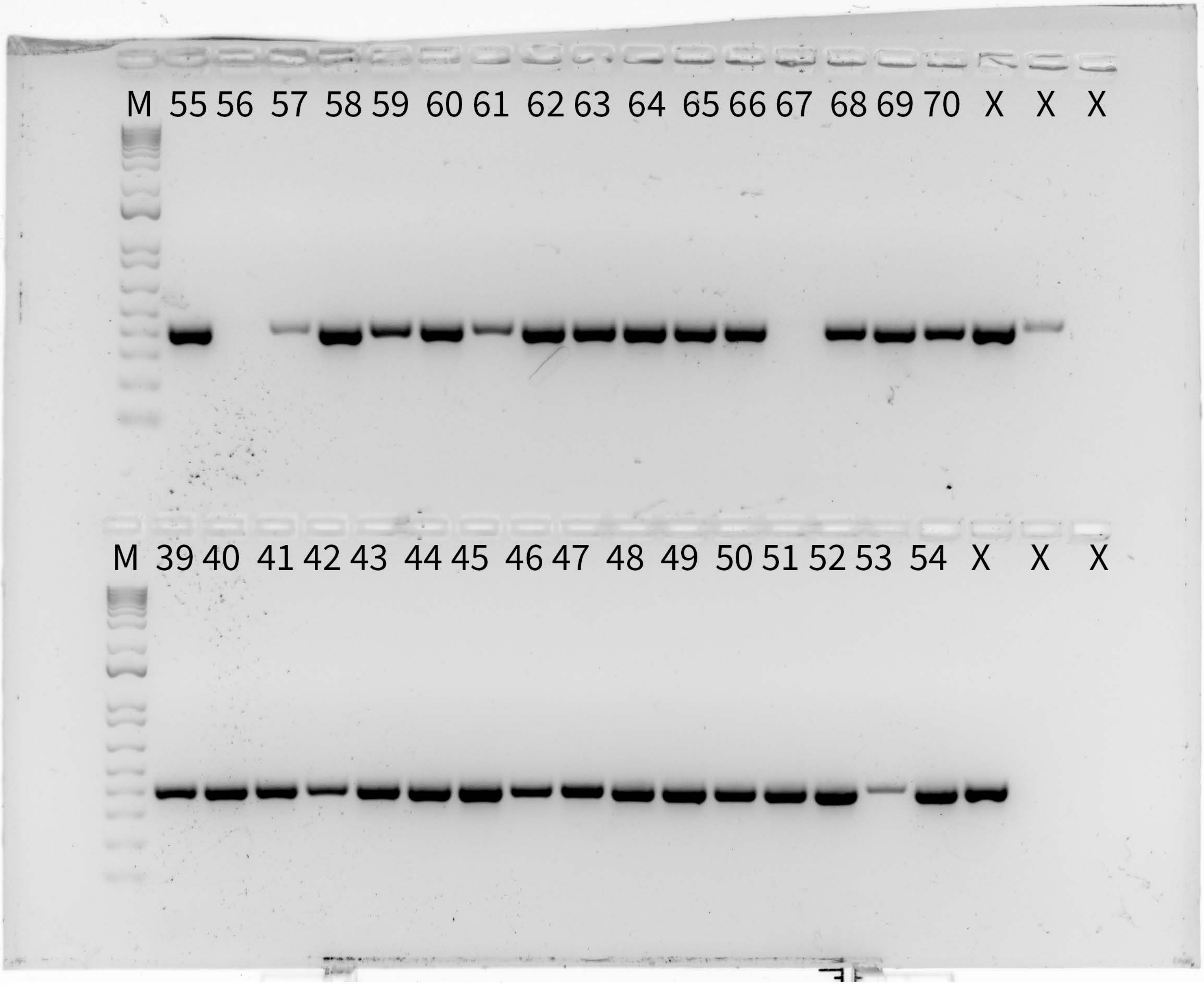

A 1 Kb Plus DNA Ladder (Invitrogen™, Thermo Fisher Scientific) was loaded in lane M and used as the molecular weight standard for estimation of PCR amplicon sizes. Gel images were acquired using an Invitrogen iBright™ 1500 Imaging System under standard imaging conditions. This original gel corresponds to S10 Fig., panel C. Lane identities are indicated above the gel, and any lanes not included in the final figure are marked with an “X” above the corresponding lane label. All annotations were added without obscuring any bands, background signal, or other image features

Source for S10 Fig. Panel D.

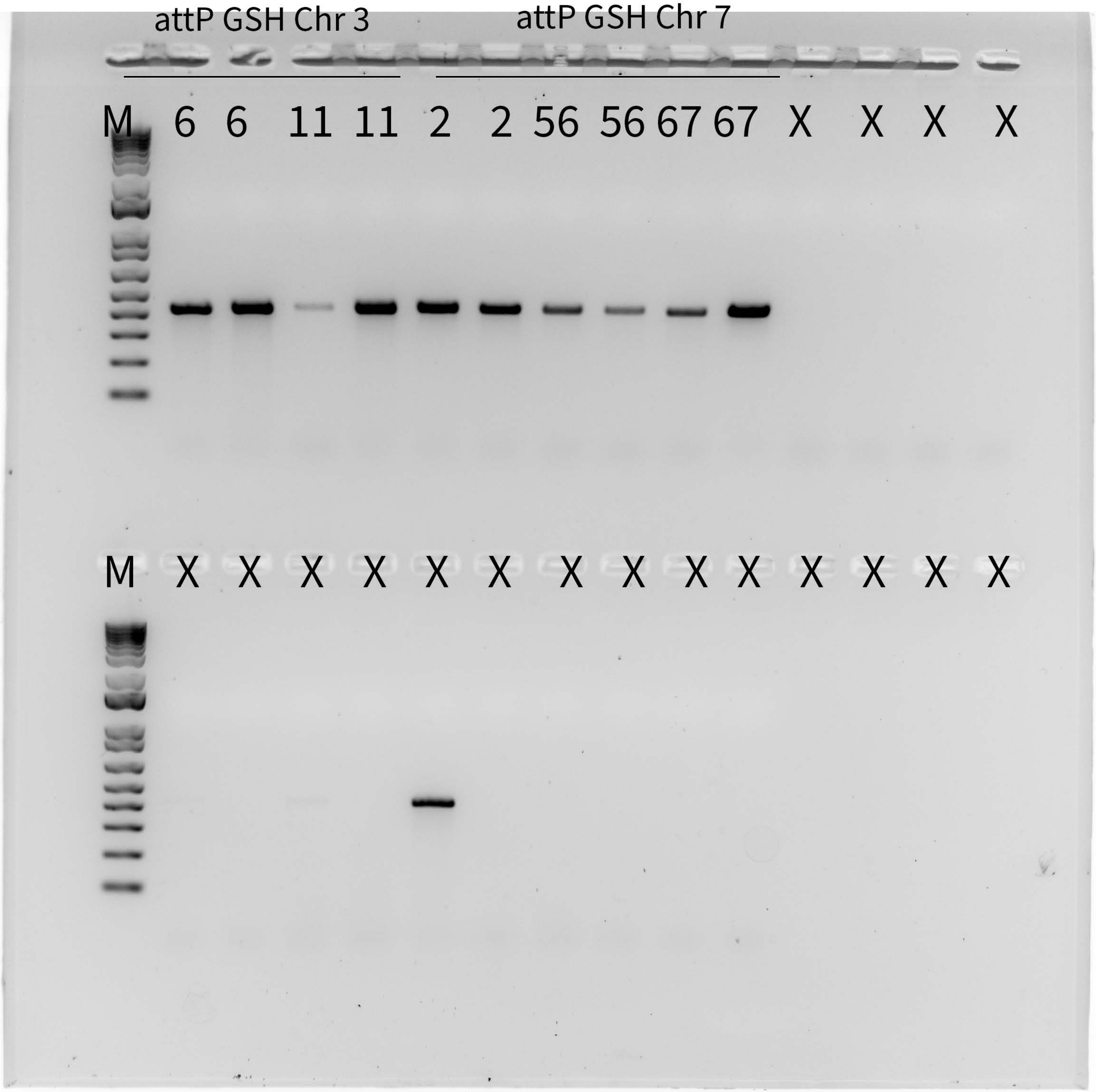

A 1 Kb Plus DNA Ladder (Invitrogen™, Thermo Fisher Scientific) was loaded in lane M and used as the molecular weight standard for estimation of PCR amplicon sizes. Gel images were acquired using an Invitrogen iBright™ 1500 Imaging System under standard imaging conditions. This original gel corresponds to S10 Fig., panel D. Lane identities are indicated above the gel, and any lanes not included in the final figure are marked with an "X" above the corresponding lane label. All annotations were added without obscuring any bands, background signal, or other image features

Source for S12 Fig. Panel D

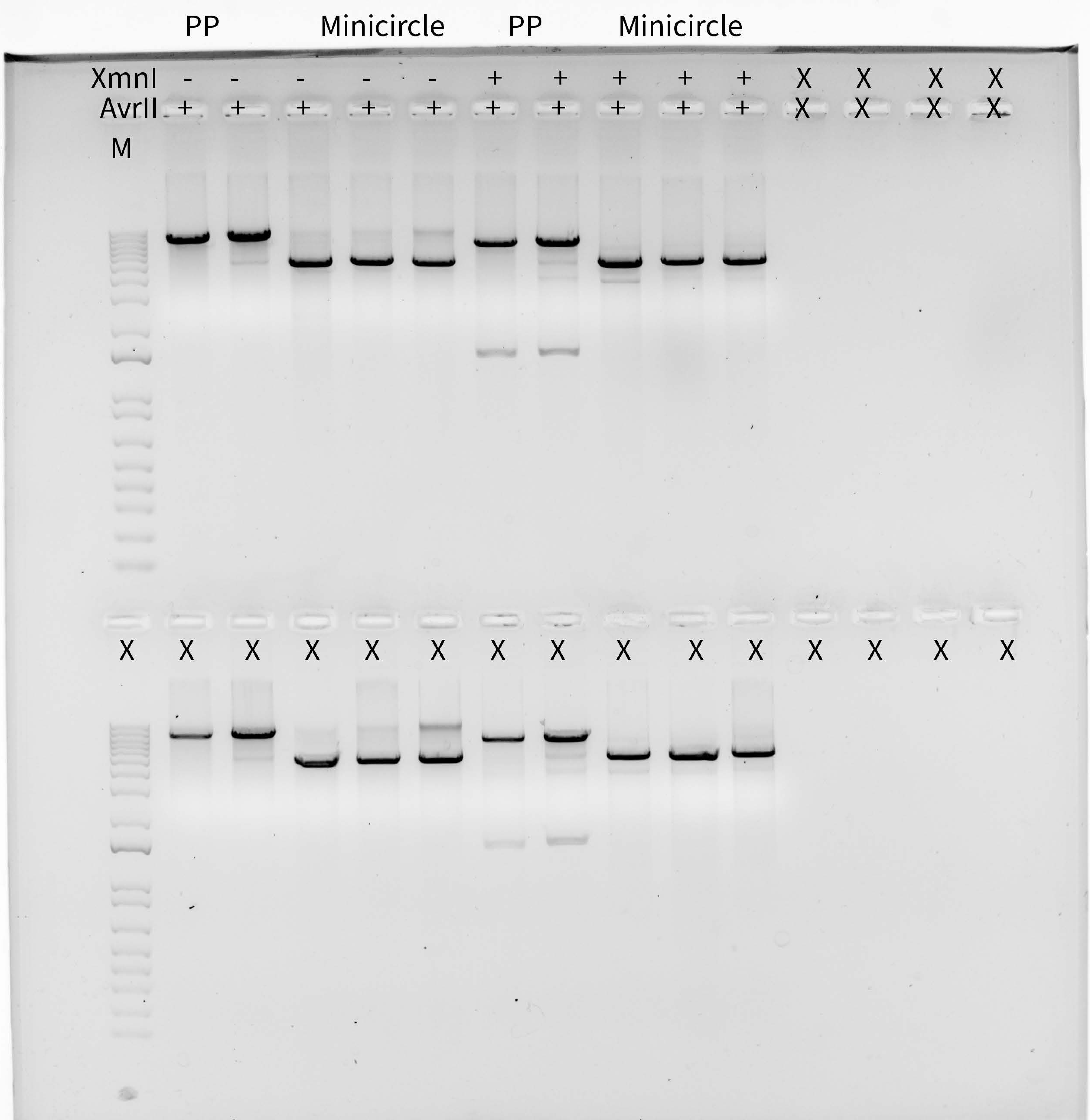

A 1 Kb Plus DNA Ladder (Invitrogen™, Thermo Fisher Scientific) was loaded in lane M and used as the molecular weight standard for estimation of PCR amplicon sizes. Gel images were acquired using an Invitrogen iBright™ 1500 Imaging System under standard imaging conditions. This original gel corresponds to S12 Fig., panel D. Lane identities are indicated above the gel, and any lanes not included in the final figure are marked with an "X" above the corresponding lane label. All annotations were added without obscuring any bands, background signal, or other image features

Source for S12 Fig. Panel F.

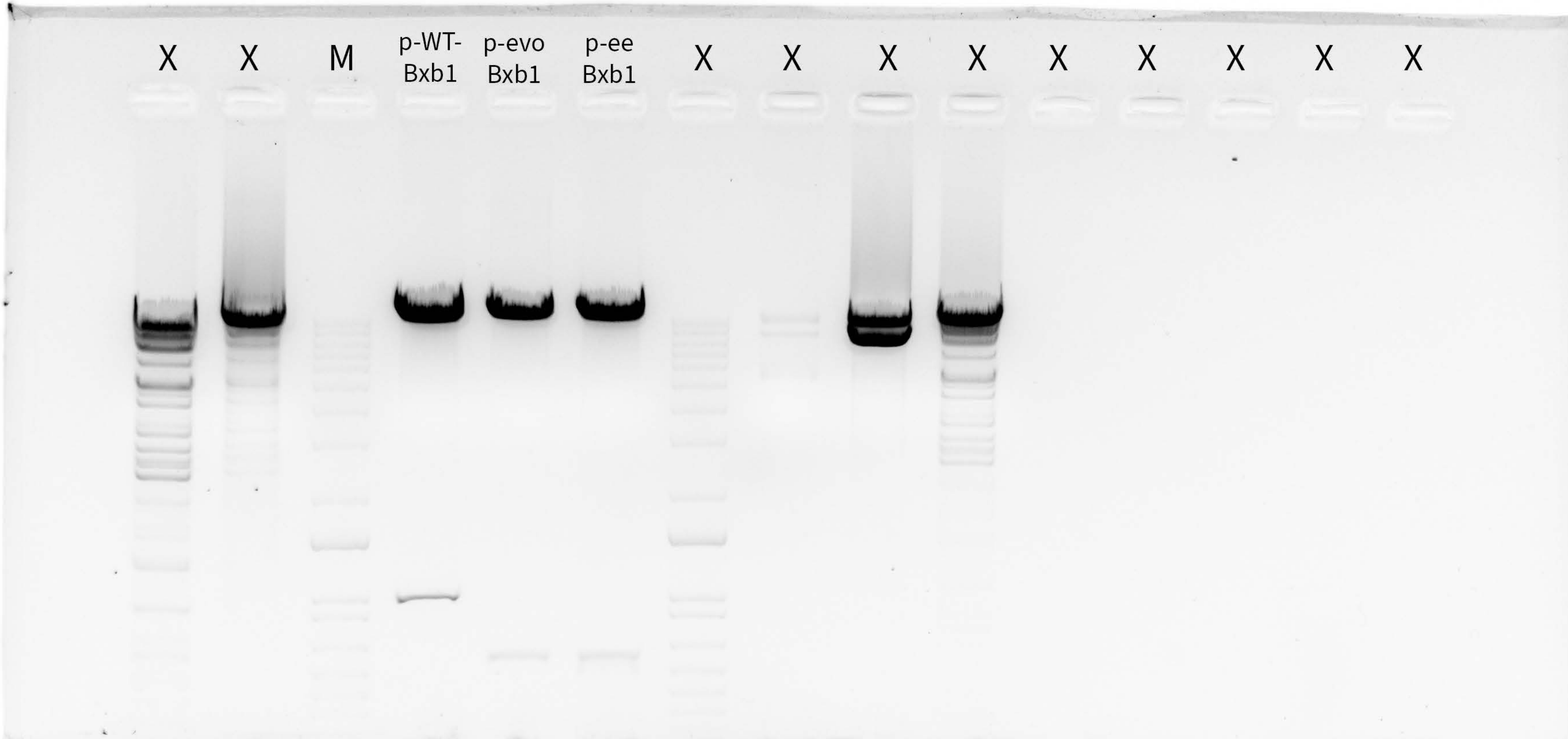

A 1 Kb Plus DNA Ladder (Invitrogen™, Thermo Fisher Scientific) was loaded in lane M and used as the molecular weight standard for estimation of PCR amplicon sizes. Gel images were acquired using an Invitrogen iBright™ 1500 Imaging System under standard imaging conditions. This original gel corresponds to S12 Fig., panel F. Lane identities are indicated above the gel, and any lanes not included in the final figure are marked with an "X" above the corresponding lane label. All annotations were added without obscuring any bands, background signal, or other image features

Source for S13 Fig. Panel B.

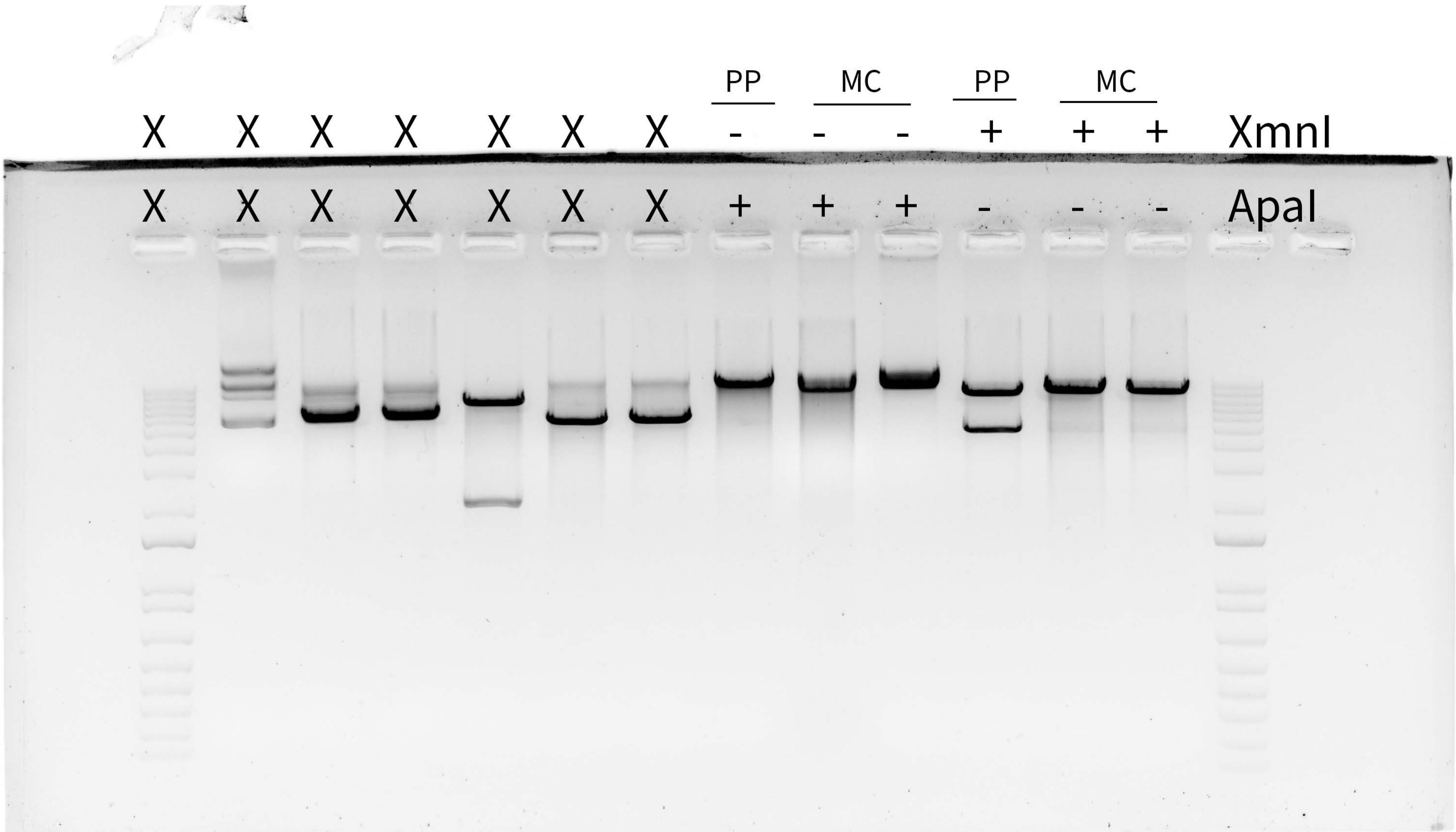

A 1 Kb Plus DNA Ladder (Invitrogen™, Thermo Fisher Scientific) was loaded in lane M and used as the molecular weight standard for estimation of PCR amplicon sizes. Gel images were acquired using an Invitrogen iBright™ 1500 Imaging System under standard imaging conditions. This original gel corresponds to S13 Fig., panel B. Lane identities are indicated above the gel, and any lanes not included in the final figure are marked with an "X" above the corresponding lane label. All annotations were added without obscuring any bands, background signal, or other image features

Source for S14 Fig. Panel A. Samples 26-28

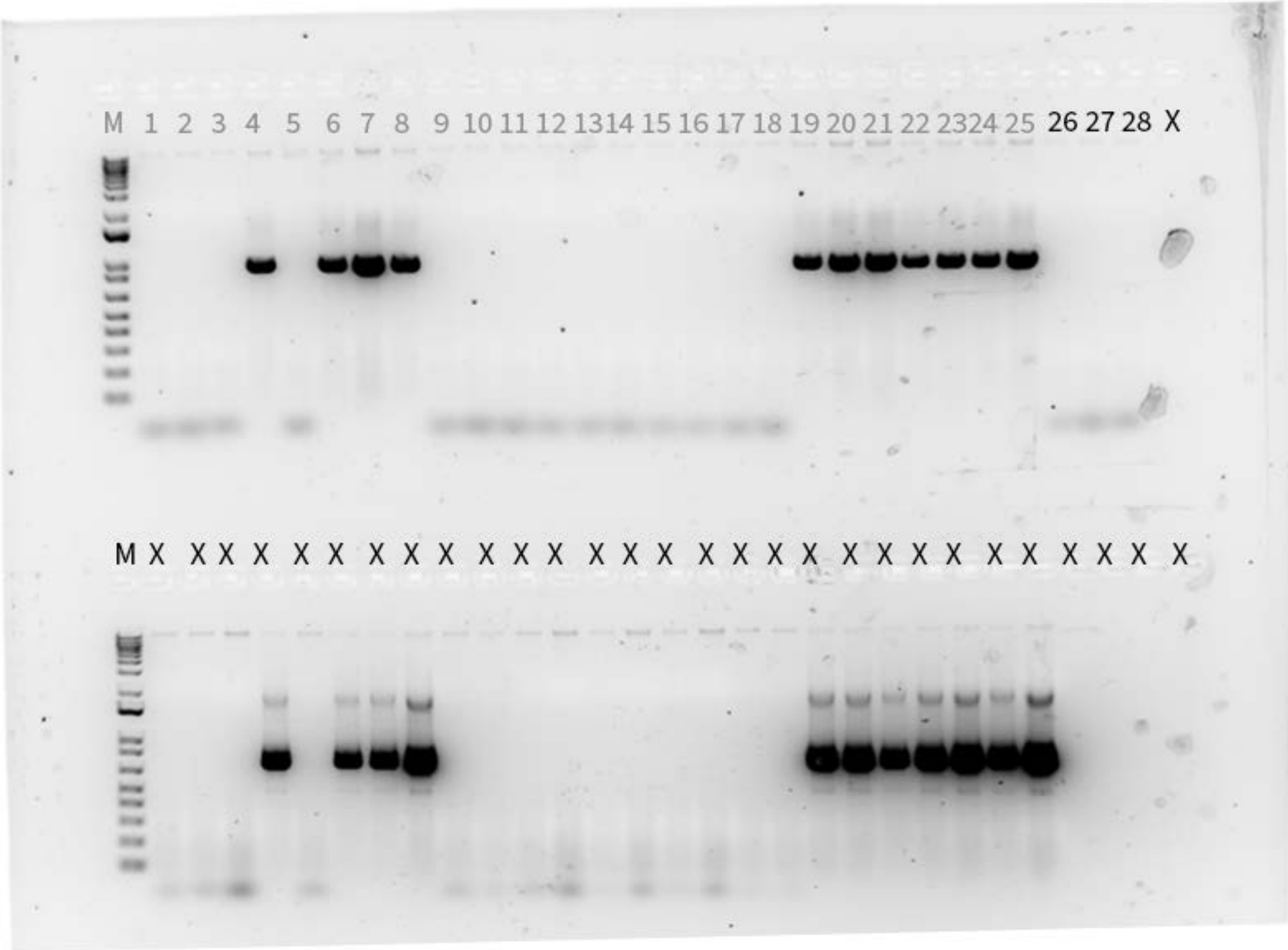

A 1 Kb Plus DNA Ladder (Invitrogen™, Thermo Fisher Scientific) was loaded in lane M and used as the molecular weight standard for estimation of PCR amplicon sizes. Gel images were acquired using an Invitrogen iBright™ 1500 Imaging System under standard imaging conditions. This original gel corresponds to S14 Fig., panel A. Lane identities are indicated above the gel, and any lanes not included in the final figure are marked with an "X" above the corresponding lane label. All annotations were added without obscuring any bands, background signal, or other image features

Source for S14 Fig. Panel A. Samples 29-53

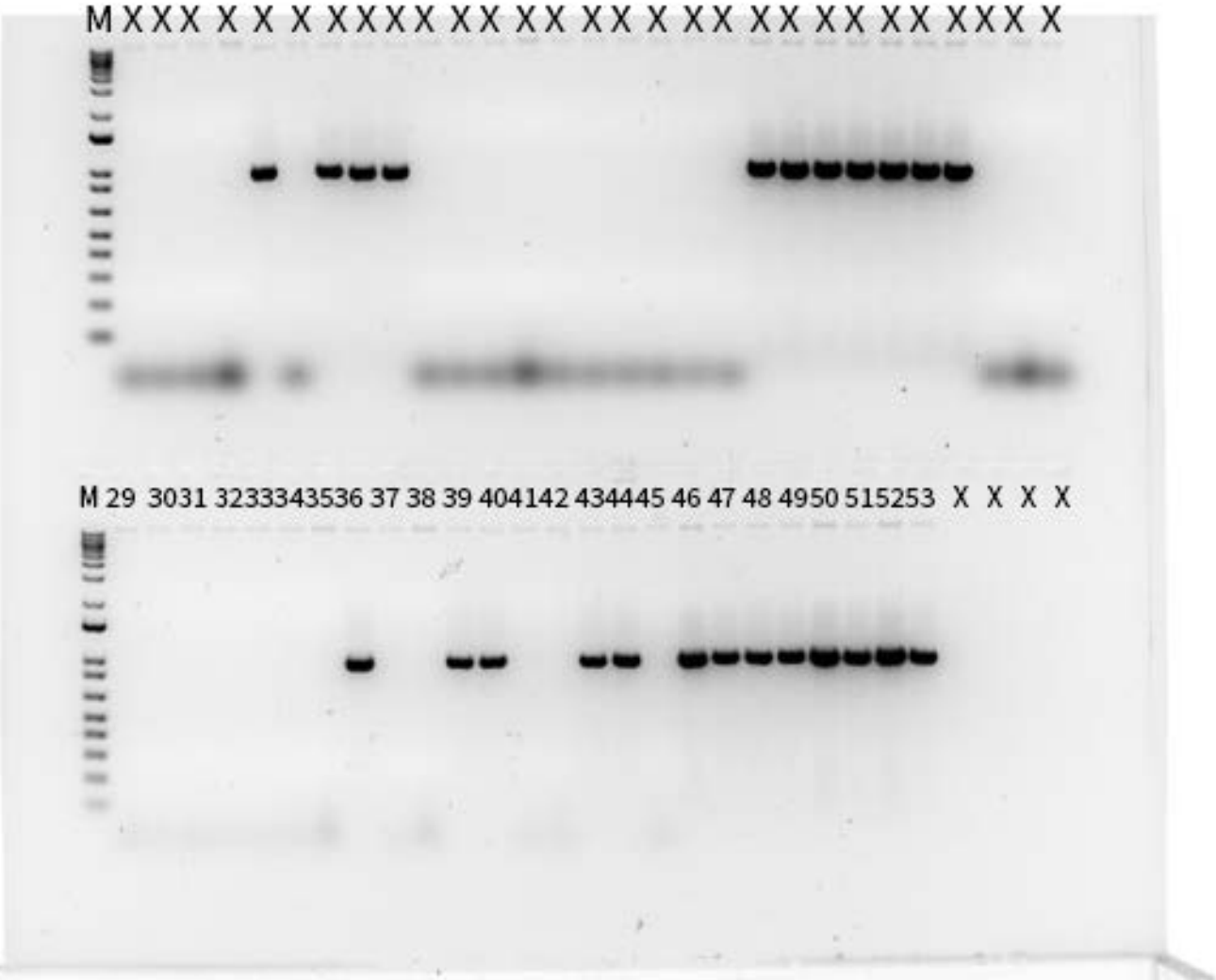

A 1 Kb Plus DNA Ladder (Invitrogen™, Thermo Fisher Scientific) was loaded in lane M and used as the molecular weight standard for estimation of PCR amplicon sizes. Gel images were acquired using an Invitrogen iBright™ 1500 Imaging System under standard imaging conditions. This original gel corresponds to S14 Fig., panel A. Lane identities are indicated above the gel, and any lanes not included in the final figure are marked with an "X" above the corresponding lane label. All annotations were added without obscuring any bands, background signal, or other image features

Source for S14 Fig. Panel B. Samples 26-53

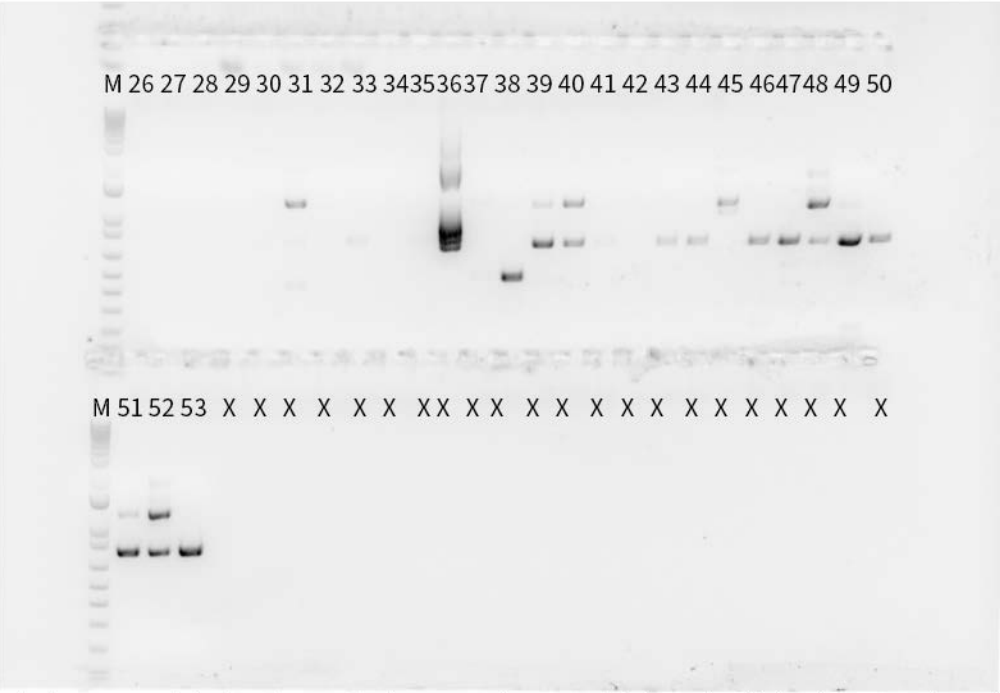

A 1 Kb Plus DNA Ladder (Invitrogen™, Thermo Fisher Scientific) was loaded in lane M and used as the molecular weight standard for estimation of PCR amplicon sizes. Gel images were acquired using an Invitrogen iBright™ 1500 Imaging System under standard imaging conditions. This original gel corresponds to S14 Fig., panel B. Lane identities are indicated above the gel, and any lanes not included in the final figure are marked with an "X" above the corresponding lane label. All annotations were added without obscuring any bands, background signal, or other image features

Source for S15 Fig. Samples 1-17

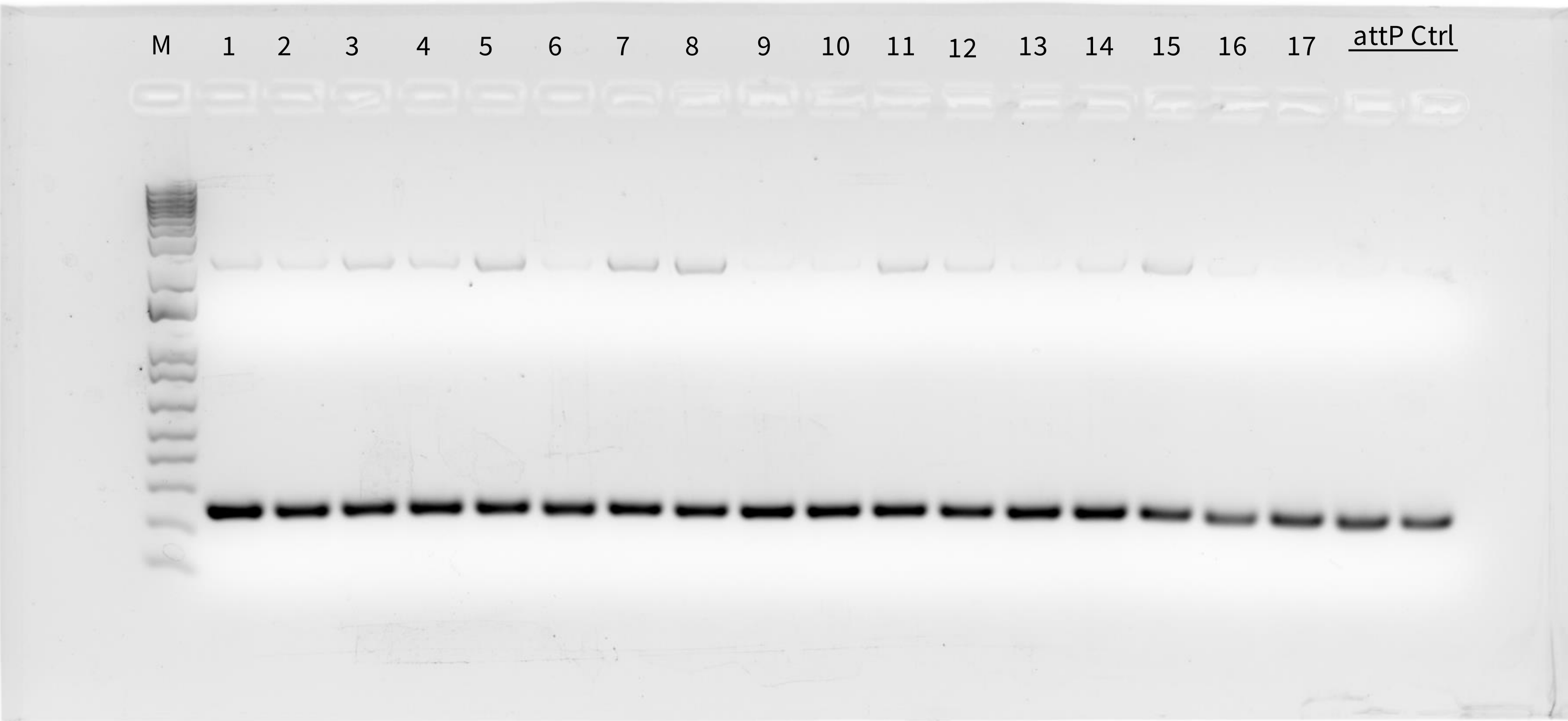

A 1 Kb Plus DNA Ladder (Invitrogen™, Thermo Fisher Scientific) was loaded in lane M and used as the molecular weight standard for estimation of PCR amplicon sizes. Gel images were acquired using an Invitrogen iBright™ 1500 Imaging System under standard imaging conditions. This original gel corresponds to S15 Fig., Samples 1-17. All annotations were added without obscuring any bands, background signal, or other image features.

Source for S15 Fig. Samples 18-34

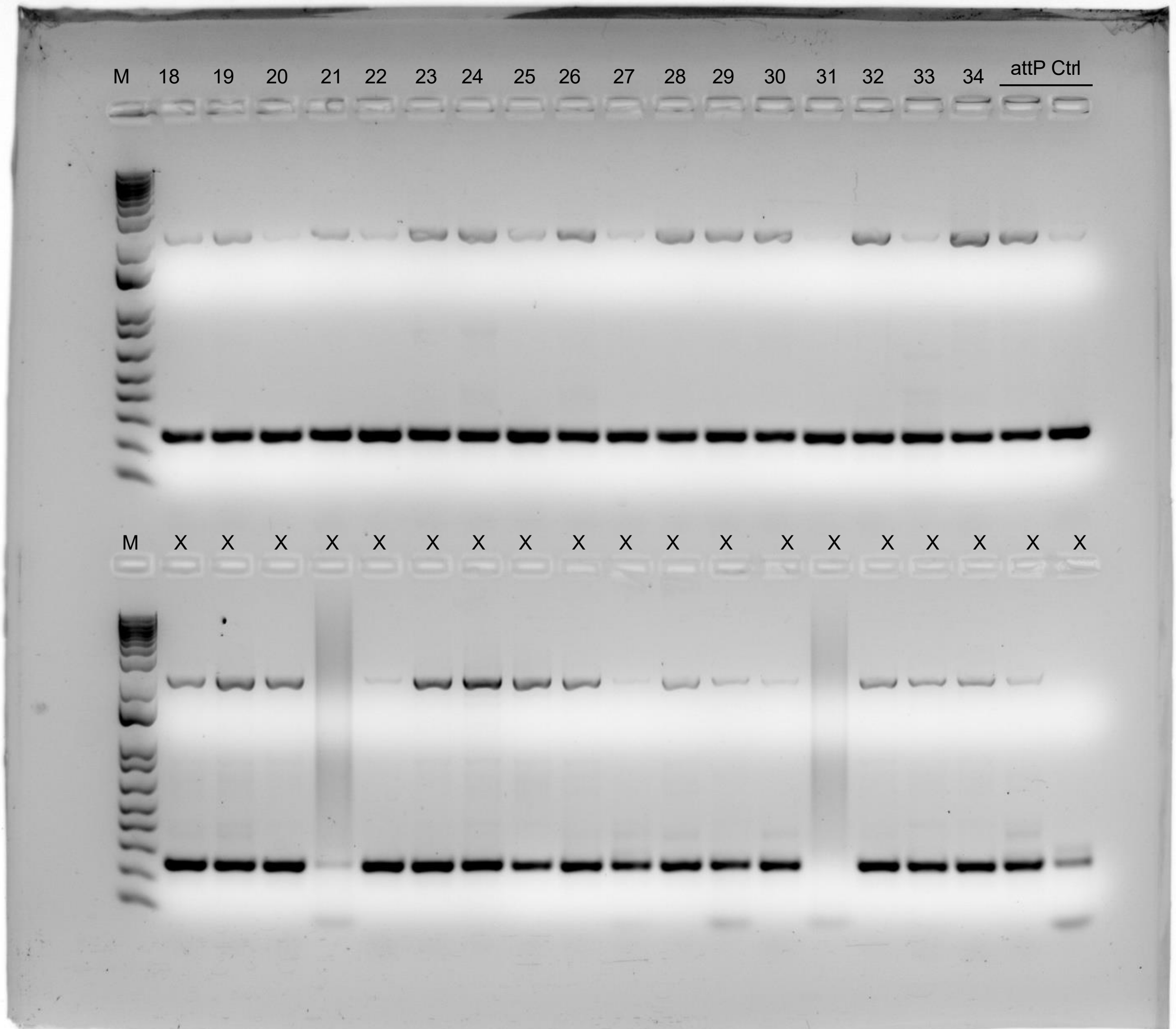

A 1 Kb Plus DNA Ladder (Invitrogen™, Thermo Fisher Scientific) was loaded in lane M and used as the molecular weight standard for estimation of PCR amplicon sizes. Gel images were acquired using an Invitrogen iBright™ 1500 Imaging System under standard imaging conditions. This original gel corresponds to S15 Fig. Samples 18-34. Lane identities are indicated above the gel, and any lanes not included in the final figure are marked with an "X" above the corresponding lane label. All annotations were added without obscuring any bands, background signal, or other image features.
