# Supplementary material for: PrimeStack editors enable efficient and site-specific integration of large multigene cargos in plants
Source: PLoS Biol. 2026 Oct 13;24(10):e3003778. doi: 10.1371/journal.pbio.3003778 (PMC13693989; doi:10.1371/journal.pbio.3003778)
Supplement: Supplementary file 2 [file pbio.3003778.s034.docx]

**Point-by-point Responses to the Reviewers’ Comments**

**Manuscript Number:** PBIOLOGY-D-26-01007R1

**Reviewer #1**

Precise, site-specific integration of large DNA sequences into plant genomes is a cornerstone of crop biotechnology and synthetic biology, yet remains constrained by random insertion, inefficient homologous recombination, and gene targeting. Here, the authors present PrimeStack, a DSB-independent platform that integrates prime editing with the unidirectional large serine integrase Bxb1, to achieve the programmable insertion of multigene, multikilobase cargos at predefined genomic safe harbors in rice. The author used the previously optimized prime editors first install attP landing sites with high fidelity and heritability followed by Bxb1-mediated recombination that generates irreversible integration of genetic information. PrimeStack achieves integration frequencies of approximately 43-46% (as detected by junction-specific PCR in rice calli). This study establishes a site-specific gene-stacking platform that enables predictable pathway construction at genomic safe harbors, thereby accelerating the development of improved and resilient crop varieties.

**Response:** We thank the reviewer for the positive evaluation and thoughtful summary of our study. In response to concerns regarding the interpretation of the data, we revised the manuscript to ensure that all claims are fully supported by the presented evidence. Specifically, statements regarding integration efficiencies and platform performance were clarified to indicate that the reported frequencies are based on junction-specific PCR and Sanger sequencing analyses and therefore represent qualitative detection of integration events rather than unbiased quantitative measurements. We also moderated comparative and forward-looking statements where direct experimental validation was not performed and expanded the Discussion to more clearly acknowledge current limitations and future validation needs, thereby improving the rigor, balance, and transparency of the manuscript.

However, the authors only provide the preliminary results with some claims either in the Abstract or in the text are not supported by the data. I have the following major and minor concerns to be addressed before consideration for acceptance of this manuscript for publication.

**Response:**

We thank the reviewer for the careful assessment of our manuscript and for highlighting concerns regarding the preliminary nature of some results and the interpretation of several claims. In response, we extensively revised the manuscript to ensure that all statements are fully supported by the presented data and that the limitations of the current study are clearly acknowledged.

Major Concerns

1. Page 10, the last paragraph and Page 11, the first paragraph, the authors should indicate how many regenerated lines are independent lines, and among these independent lines, how many lines are precisely edited (heterozygous and/or homozygous), how many lines are non-precisely edited, how many are WT lines, respectively. I would like to suggest the authors add a Table in the text or in Supplementary Data. In this way, you can evaluate the performances of three prime editor variants in rice stable lines. To be honest, I don't think employing amplicon NGS could provide statistical comparison of the performances of these three prime editor variants.

**Response:**

Thank you for this valuable suggestion. In response, we have added the **S7 Table** (also included below for convenience) to the Supplementary Data, summarizing the genotyping outcomes of regenerated independent rice lines obtained with each prime editor variant. The table includes the total number of independent lines analyzed, as well as the number of precisely edited lines (characterized as homozygous, heterozygous, or chimeric), non-precisely edited lines, and WT lines. This information has been incorporated into both the manuscript text and the Supplementary Data.

We agree with the reviewer that even the inclusion of amplicon deep sequencing (NGS) may not enable a robust statistical comparison of the performance of the three prime editor variants. Therefore, in addition to the newly added **S7 Table,** which provides an initial assessment of the relative performance of the three prime editor variants in stable rice lines. We produced the transgenic plants with all three prime editors, but only PE6c showed efficient *attP* insertion at the target sites. We then focused on PE6c and produced homozygous plants with *attP* sites.

We have removed the following statement from the first paragraph of page 11:

“Because genotyping was performed by conventional PCR rather than amplicon deep sequencing (NGS), the data do not permit per-plant quantification of allele frequency or discrimination between low-level chimerism and stable mono/bi-allelic insertion (Fig. 2E). Future studies employing amplicon NGS would provide higher-resolution quantification and enable formal statistical comparison of prime editor variants.”

Please also note that the primary objective of these transformation experiments was not to perform a comparative evaluation of the prime editor variants, but rather to generate rice lines carrying the *attP* insertion sites required for subsequent experiments.

**S7 Table**

| Prime editor | Target site | Independent lines | Homozygous precise | Heterozygous precise | Chimera | Non-precise | WT |
| --- | --- | --- | --- | --- | --- | --- | --- |
| PE6c | Chr 1-2 | 13 | 2 | 6 | 4 | 1 | 0 |
|  | Chr 3 | 12 | 1 | 8 | 1 | 2 | 0 |
|  | Chr 7 | 10 | 0 | 0 | 2 | 0 | 8 |
|  | Chr 11 | 400 | 1 | 0 | 0 | 35 | 364 |
|  | OsMOC1 | 8 | 1 | 0 | 0 | 0 | 7 |
| PE6d | Chr 1-2 | 5 | 0 | 0 | 0 | 0 | 5 |
|  | Chr 3 | 5 | 0 | 0 | 1 | 0 | 4 |
|  | Chr 7 | 5 | 0 | 0 | 0 | 0 | 5 |
|  | Chr 11 | 5 | 0 | 0 | 0 | 0 | 5 |
|  | OsMOC1 | 5 | 0 | 0 | 0 | 0 | 5 |
| PEmax | Chr 1-2 | 5 | 0 | 0 | 0 | 0 | 5 |
|  | Chr 3 | 5 | 0 | 0 | 0 | 0 | 5 |
|  | Chr 7 | 5 | 0 | 0 | 0 | 0 | 5 |
|  | Chr 11 | 5 | 0 | 0 | 0 | 0 | 5 |
|  | OsMOC1 | 5 | 0 | 0 | 0 | 0 | 5 |

2. Page 13, I am wondering if the authors only tested the performance of PrimeStack in calli with pre-installed attP site?

**Response:**

Thank you for this insightful comment. In this study, the performance of PrimeStack was evaluated in regenerated rice plants derived from calli carrying pre-installed *attP* landing pads. While page 13 describes our preliminary results in callus, pages 15-16 report the number of regenerated plants derived from shoot regeneration of the *attP*-engineered callus.

To be informative to the readers, the authors need to provide the information in the text on how many independent lines carrying the precisely integrated carotenoid synthesis cassettes, the ratios of heterozygous and/or homozygous lines, has been obtained? This information would benefit the readers to get an initial view on the efficiency of the PrimeStack platform established in this study.

**Response:**

To characterize targeted integration events, we performed junction-PCR followed by Sanger sequencing using primers specific to the insertion junctions, an approach selected due to the large size of the integrated cassette. However, this strategy does not allow accurate determination of zygosity. Therefore, we evaluated the presence of the unrecombined *attP*-containing allele using primer pair EC13 and EC14, which anneal to genomic regions flanking the *attP* site. Clear 263 bp amplicons were detected in all the 34 regenerated plants previously confirmed to be positive for both left and right integration junctions. Because these plants were also positive for the left and right integration junctions, the results suggest that the regenerated lines are likely heterozygous. However, the possibility that some regenerated plants are chimeric cannot be excluded.

A figure showing the identification of the *attP* allele in regenerated plants has been added as **S15 Fig** and it is also included below for convenience. Furthermore, a corresponding paragraph describing the analysis presented in S15 Fig. and explaining its results has been added to the manuscript. *attP* Ctrl; the homozygous plants carrying *attP* sites were used as a positive control.

Furthermore, are these precisely edited lines with carotenoid synthesis cassettes morphologically normal in comparison to the WT control? In addition, it would be also interesting to investigate whether the carotenoid content has been increased in the rice brown grains and polished grains.

**Response:**

We thank the reviewer for this important comment. Importantly, regenerated plants positive for the targeted integration junctions displayed normal morphology comparable to WT plants under the evaluated growth conditions, and no obvious phenotypic abnormalities were observed during growth. For reference, we provide a representative image of regenerated plants positive for the 5.3 kb carotenoid cassette integration alongside a WT control plant. The plants shown were photographed five weeks after transfer to soil, and no obvious phenotypic differences were observed. This image has been included as **S16 Fig**., and a corresponding statement describing the phenotypic evaluation has been added to the manuscript.

Plant 4

eeBxb1

Plant 2

evoBxb1

WT plant

In addition, the seeds derived from these T0 plants did not show an obvious phenotype associated with carotenoid production. However, further analysis is required to test the segregation patterns, homozygosity, transgenerational stability, and carotenoid accumulation in grains. We agree that these evaluations will be important for the comprehensive characterization of the generated lines and represent an important direction for future work.

3. Although this study establishes a platform, PrimeStack, which enables site-specific integration of kilobase-scale DNA into plant genomic safe harbors, benefiting the integration of foreign genes/cassettes at these safe harbors efficiently. The presences of pre-installed recognition sites attP landing sites inside the agriculturally important endogenous beneficial genes/alleles loci still raise biosafety concerns. I would suggest the authors present a few sentences to address the limitation of this study in the Discussion section.

**Response:**

Thank you for this important comment. We agree that the introduction of an exogenous *attP* sequence into a plant endogenous genomic loci may raise regulatory and biosafety considerations. In response, we have added a paragraph to the Discussion section acknowledging this limitation of the current study.

“Third, from a regulatory and biosafety standpoint, the Bxb1 *attP* and *attB* recognition sequences used here are 38-50 bp in length. As an example of one of the most stringent regulatory frameworks currently under discussion, the European Commission proposal regarding New Genomic Techniques (NGTs) (COM(2023) 411) generally limits Category 1 NGT plants to insertions or substitutions of no more than 20 nucleotides. Therefore, lines carrying these *attP* sites may not fall within these criteria and could remain subject to full GMO regulatory oversight depending on the jurisdiction and final genomic configuration of the engineered plants [73].”

Reference:

European Commission. Proposal for a Regulation of the European Parliament and of the Council on plants obtained by certain new genomic techniques and their food and feed, and amending Regulation (EU) 2017/625. Brussels2023.

4. Page 14, the last sentence in the first paragraph, the authors mentioned that 'Although regeneration of plants carrying the dual trait donor was not achieved under the tested selection conditions, the robust molecular validation at the callus stage establishes successful site-specific stacking of multiple traits at a single genomic locus'. The novelty of this study is somehow compromised without the obtainment of regenerated lines with precisely integrated dual gene cassettes. Are there any reasons underlying this phenomenon?

**Response:**

We appreciate the reviewer raising this important point. Several factors may have contributed to the inability to recover regenerated plants carrying the dual-trait cassette despite detection of targeted integration events in callus tissue. These may include: (i) suboptimal bispyribac sodium selection conditions, as excessively stringent selection could negatively affect callus viability and regeneration efficiency; (ii) the larger size and increased complexity of the dual-trait cassette, which may reduce the recovery of successfully regenerated events during prolonged tissue culture and selection; (iii) potential effects of construct architecture and co-delivery efficiency on transformation outcomes and stable regeneration; and (iv) chimeric or low-frequency targeted integration events that were detectable at the callus stage but were not efficiently maintained or recovered during plant regeneration.

Importantly, the detection of correctly targeted integration events in callus tissue supports the capacity of PrimeStack to mediate site-specific stacking of multiple traits at a single genomic locus. Nevertheless, recovery of regenerated dual-trait plants remains an area for future research.

5. Page 15 and 16, in the section 'Site-specific integration efficiencies using engineered Bxb1 variants'. As I mentioned previously, the authors need to add a Table either in the text or in Supplementary Data to provide the detailed information on independent lines obtained, of which, the number of precisely edited lines, heterozygous or homozygous, in order to evaluate the relative efficiency of each Bxb1 variants. Besides, to provide the proof of the concept that the integration of foreign gene/cassette at the identified safe harbor through PrimeStack indeed has no side-effects on agronomic traits and its utility in crop improvement, I am wondering whether these precisely edited lines with carotenoid synthesis cassettes morphologically normal in comparison to the WT control? In addition, it would be also interesting to investigate whether the carotenoid content has been increased in the rice brown grains and polished grains.

**Response:**

Thank you for this valuable comment. As noted in our response to Major Concern 2, we performed junction-PCR followed by Sanger sequencing using primers specific to the insertion junctions, an approach selected due to the large size of the integrated cassette. However, this strategy does not allow accurate determination of zygosity. Therefore, as described previously, we performed additional characterization of the *attP*-containing allele. All 34 plants positive for the left and right integration junctions were also positive for the *attP* allele, suggesting that the regenerated lines are likely heterozygous, although the possibility of chimerism cannot be excluded. In addition, we included representative images comparing regenerated plants with a WT control, showing no obvious phenotypic differences under the evaluated growth conditions.

In addition, the seeds derived from these T0 plants did not show an obvious phenotype associated with carotenoid production. However, further analysis is required to test the segregation patterns, homozygosity, transgenerational stability, and carotenoid accumulation in grains. We agree that these evaluations will be important for the comprehensive characterization of the generated lines and represent an important direction for future work.

Minor concerns

1. In the Abstract section, several phrases need to be revised. For example, 'comparing favorably with bidirectional Cre-lox systems' is there any data to support this claim?

**Response:**

We thank the reviewer for this important observation. We agree that the statement “comparing favorably with bidirectional Cre-lox systems” was not directly supported by experimental comparisons within the present study. In response, we have removed this statement from the Abstract to avoid overinterpretation and to more accurately reflect the scope of the data presented.

2. Remove all the Figure legends to the end of text.

**Response:**

Thank you for this comment. The figure legends are already organized in ascending numerical order following their respective citations in the manuscript, in accordance with the journal formatting guidelines.

3. The authors used biolistic co-delivery of a carotenoid minicircle donor and Bxb1 recombinase into attP engineered callus. I am wondering why the authors used biolistic delivery but not the Agrobacterium-mediated transformation. Is the biolistic delivery more efficient than Agrobacterium-mediated transformation in this case?

**Response:**

We thank the reviewer for this important question. In this study, we used biolistic co-delivery because the donor DNA was engineered as a minicircle construct lacking the *Agrobacterium* origin of replication and associated bacterial backbone sequences required for *Agrobacterium*-mediated transfer and maintenance (elements of the binary vectors). Therefore, the minicircle donor is not compatible with standard *Agrobacterium* delivery systems. In addition, biolistic transformation enabled the simultaneous physical co-delivery of both the minicircle donor DNA and the Bxb1 recombinase directly into the *attP*-engineered rice callus cells. This approach was selected to facilitate efficient transient delivery and recombination while minimizing the integration of unwanted vector backbone sequences. We do not necessarily claim that biolistic delivery is generally more efficient than *Agrobacterium*-mediated transformation. Rather, in this specific experimental setup, biolistic delivery was the most suitable method due to the structural design of the minicircle donor and the need for direct co-delivery of donor DNA and recombinase components.

4. Page 7, the authors mentioned 'The final panel comprised two targets within a genomic safe harbor shared across 33 rice genomes on chromosome 1'. Here, it is not clear about the meaning of 'across 33 rice genomes'.

**Response:**

We thank the reviewer for pointing out this lack of clarity. In the revised manuscript, we have modified this sentence to better explain that the selected genomic safe harbor region on chromosome 1 was identified as highly conserved across 33 rice genome assemblies analyzed in the referenced study, indicating that this locus is broadly maintained among diverse rice accessions.

Referenced study: Sun C, Lei Y, Li B, Gao Q, Li Y, Cao W, et al. Precise integration of large DNA sequences in plant genomes using PrimeRoot editors. Nature Biotechnology. 2024;42(2):316-27. doi: 10.1038/s41587-023-01769-w.

Reviewer #2

The authors showcase a novel recombinase-integration system for plant genome editing using the Bxb1 system, with prior prime-editing mediated recognitions site installation in genomic safe harbors. Compared to a similar system using Cre in plants, this work provides a directional and irreversible recombination system, which can have valuable applications in the future, though the present data shows a low level of recombination efficiency.

**Response:**

We thank the reviewer for recognizing the potential value of the PrimeStack platform for directional and irreversible site-specific integration in plants. We agree that, although the present study demonstrates successful Bxb1-mediated targeted integration in rice, further optimization will be required to improve recombination efficiencies and maximize the robustness of the system for broader applications. We believe that the modular nature of PrimeStack provides a foundation for future improvements in donor delivery, recombinase engineering, landing pad design, and plant regeneration strategies.

Major Comments

1. The abstract sentence on efficiency can be interpreted to mean 43-46% of regenerated lines have a stably heritable allele of the targeted large insertion. It is not as clear as in the body text in discussing that this is the % of calli with any detectable large insertion. Otherwise, the findings are suitable to support the assertions of efficient recombination installation by PE6c and a qualitative achievement of large-cargo Bxb1 insertion.

**Response:**

We thank the reviewer for this important observation. We agree that the original wording in the Abstract could be interpreted as implying stable heritable integration frequencies. The reported 43-46% values correspond to the percentage of regenerated rice plants in which targeted large-cargo integration events were detected by junction-specific PCR analyses. We changed the word “calli” to “plants” in the revised version of the manuscript. The revised sentence is as below:

“PrimeStack enabled targeted large-cargo integration in regenerated rice plants with efficiencies of up to 45%, as determined by junction-specific PCR analyses. The regenerated plants displayed normal phenotypes under the evaluated growth conditions.”

This revised wording more clearly indicates that the reported values correspond to PCR-detected targeted integration events in regenerated plants rather than stable heritable insertions.

We have also included a statement in the Results section (page 16) specifying that these percentages were derived from junction-specific PCR and Sanger sequencing analyses in order to more clearly reflect the qualitative nature of the data.

Minor Comments

1. Abstract text, line 1, "site-specific integration of large DNA sequences…" cannot be a "cornerstone" if it is hard to achieve. The idiomatic meaning of cornerstone implies it is a foundational aspect. Consider rewording "cornerstone" to "long-standing goal" or something else.

**Response:**

Thank you for this helpful suggestion. We agree that the term “cornerstone” may inaccurately imply that site-specific integration of large DNA sequences is already an established and routinely achievable capability in plant biotechnology. To better reflect the current state of the field, we have revised the text by replacing “cornerstone” with “long-standing goal.”

2. Please ensure consistency and accurate notation of genes in italics and proteins not in italics. Please ensure this for the figures as well. Example: figure 3 legend, "attB" not italicized.

**Response:**

We thank the reviewer for pointing out these inconsistencies. We have carefully revised the manuscript and figures to ensure consistent and accurate notation throughout, including italicization of gene names and DNA sequence elements where appropriate, while protein names remain non-italicized. The notation of elements such as *attB* and *attP* has also been corrected in the figure legends and throughout the manuscript.

3. Please ensure high resolution figures for the final submission.

**Response:**

We thank the reviewer for this comment. High-resolution versions of all figures have been prepared and will be provided for the final submission to ensure optimal image quality and readability.

Reviewer #3

This manuscript by Sánchez et al. reported the development of PrimeStack, a DSB-free genome engineering platform that combines prime editing (PE) with Bxb1 integrase and its evolved variants. This system enables the programmable and irreversible insertion of multi-gene cassettes into experimentally validated genomic safe harbors (GSHs) in rice. Nevertheless, multiple deficiencies need to be addressed and revised prior to acceptance for publication.

**Response:**

We thank the reviewer for the careful evaluation of our manuscript and for recognizing the development and potential applications of the PrimeStack platform for DSB-free programmable genome engineering in plants. We appreciate the reviewer’s constructive comments and suggestions, which have helped us improve the clarity, accuracy, and overall quality of the manuscript. We have carefully addressed each concern raised and revised the manuscript accordingly. Detailed point-by-point responses are provided below.

1. The authors designed eight GSH loci and two sites adjacent to translational regions as attP target sites, and acquired transgenic materials with attP insertion at four GSH loci and the MOC1-proximal site. However, only the Chr1-2 line was selected for large-fragment insertion testing, and the attP-inserted line near the MOC1 locus was not included as a control. With only one single locus (Chr1-2) and two insertion events (5.3 Kb and 9.4 Kb) characterized, it remains insufficient to fully validate the reliability and stability of this large DNA fragment editing system.

**Response:**

Thank you for this important comment. We agree that evaluation across additional genomic loci would further strengthen validation of the PrimeStack platform and provide insight into locus-dependent effects on integration efficiency and stability.

In this study, our primary objective was to establish PrimeStack-mediated large DNA integration in plants. For downstream experiments, we selected the Chr1-2 line because it consistently showed robust *attP* installation and favorable T-DNA segregation compared to the other candidate loci. Using this site, we demonstrated targeted integration of two independent cargo sizes (5.3 kb and 9.4 kb), supporting the feasibility of kilobase-scale DNA insertion in rice.

Importantly, successful *attP* installation was achieved across multiple GSH loci, indicating that the first step of the PrimeStack workflow is not restricted to a single genomic position. Due to the extensive timeline associated with rice transformation, regeneration, and molecular characterization, a broader multi-locus integration analysis was not included in the present study. In addition, previous studies, including the reported PrimeRoot system (ref), have demonstrated the feasibility of prime editing-based strategies for targeted large DNA integration in plants, although using different recombination architectures.

Future work will further evaluate PrimeStack performance across additional genomic loci and endogenous genomic contexts.

Reference:

Sun C, Lei Y, Li B, Gao Q, Li Y, Cao W, et al. Precise integration of large DNA sequences in plant genomes using PrimeRoot editors. Nature Biotechnology. 2024;42(2):316-27. doi: 10.1038/s41587-023-01769-w.

2. Three PE systems (PEmax, PE6c and PE6d) were constructed for attP insertion, and three recombinases (eeBxb1, evoBxb1 and wild-type Bxb1) were adopted in parallel. Notably, Sanger sequencing was exclusively used for post-transformation comparison of different systems, which only supports qualitative analysis. The rationale for not adopting NGS for quantitative analysis is unclear, especially considering that eeBxb1, evoBxb1 and Bxb1 exhibited highly similar performance in 5.3 Kb large-fragment insertion.

**Response:**

We thank the reviewer for this important observation. We agree that amplicon deep sequencing (NGS) would provide higher-resolution quantitative information regarding editing frequencies, allelic distributions, and potential differences among recombinase variants. However, due to the substantial experimental and financial requirements associated with large-scale NGS analysis of regenerated rice lines carrying kilobase-scale integrations, such analyses were not performed in the present study. To avoid overinterpretation, we revised the manuscript to moderate statements implying quantitative comparison among recombinase variants and clarified the qualitative nature of the current analyses.

Our primary objective was to establish the feasibility of PrimeStack-mediated targeted large DNA integration in plants. Our analyses of large DNA integration systems in plants have relied on junction PCR and Sanger sequencing for validation of targeted insertion events as shown in previous studies^1,2^.

References:

1. Sun C, Lei Y, Li B, Gao Q, Li Y, Cao W, et al. Precise integration of large DNA sequences in plant genomes using PrimeRoot editors. Nature Biotechnology. 2024;42(2):316-27. doi: 10.1038/s41587-023-01769-w.

2. Dong OX, Yu S, Jain R, Zhang N, Duong PQ, Butler C, et al. Marker-free carotenoid-enriched rice generated through targeted gene insertion using CRISPR-Cas9. Nature Communications. 2020;11(1):1178. doi: 10.1038/s41467-020-14981-y

3. The authors have obtained transgenic lines with 5.3 kb fragment insertion and confirmed the insertion accuracy by sequencing, but no phenotypic data have been provided to verify and demonstrate the functional effects of exogenous gene insertion.

**Response:**

We thank the reviewer for this important comment. The regenerated plants carrying the 5.3 kb carotenoid cassette integration did not display obvious morphological differences compared to WT plants under the evaluated growth conditions. To illustrate this observation, we included a representative image of regenerated plants positive for the 5.3 kb carotenoid cassette integration alongside a WT control plant as **S16 Fig**. and added a corresponding statement in the manuscript.

Plant 4

eeBxb1

Plant 2

evoBxb1

WT plant

In addition, the seeds derived from these T0 plants did not show an obvious phenotype associated with carotenoid production. However, further analysis is required to test the segregation patterns, homozygosity, transgenerational stability, and carotenoid accumulation in grains. We agree that these evaluations will be important for the comprehensive characterization of the generated lines and represent an important direction for future work.

4. Three different recombinases were also applied for 9.4 Kb large-fragment insertion, and successful fragment integration was detected at the rice callus stage. However, comparative data among the three recombinases are missing. The performance and functional differences of eeBxb1, evoBxb1 and Bxb1 in facilitating 9.4 Kb large-fragment insertion should be supplemented and clarified.

**Response:**

We thank the reviewer for this important observation. We agree that comparative analyses among eeBxb1, evoBxb1, and wild-type Bxb1 would provide valuable insight into their relative performance in mediating 9.4 kb large-fragment integration. We collected ten pieces of calli and pooled them into a single sample. A total of ten pooled samples were genotyped for each recombinase. The data is presented in the **S8 Table**, as shown below. The following sentence is revised in the manuscript “Sanger sequencing confirmed accurate left and right recombination junctions (Fig 4E-F), with efficiency 40-50% (S8 Table).”

| Recombinase variant | Total number of tested calli | Positive calli | Efficiency (%) |
| --- | --- | --- | --- |
| eeBxb1 | 10 | 4 | 40 |
| evoBxb1 | 10 | 5 | 50 |
| WT Bxb1 | 10 | 4 | 40 |
