# Supplementary material for: PrimeStack editors enable efficient and site-specific integration of large multigene cargos in plants
Source: PLoS Biol. 2026 Oct 13;24(10):e3003778. doi: 10.1371/journal.pbio.3003778 (PMC13693989; doi:10.1371/journal.pbio.3003778)
Supplement: Supplementary file 3 [file pbio.3003778.s035.docx]

**Response to the Reviewers**

**Manuscript ID: PBIOLOGY-D-26-01007R2**

**Title:** “High-efficiency, site-specific integration of kilobase-scale DNA into plant genomic safe harbors via PrimeStack editors”

Dear Reviewers,

We sincerely thank you for the careful evaluation of our revised manuscript and for your constructive comments and suggestions. We have carefully considered every comment and revised the manuscript accordingly.

Below, each reviewer comment is reproduced in full in italics, followed by our response. All corresponding revisions are shown in the separate “Revised Article with Changes Highlighted” file, and the relevant locations in the revised manuscript are indicated in our responses.

**Reviewer #2**

*The authors have addressed all my points. I have no more concerns.*

**Response:** We thank Reviewer #2 for the positive assessment and for their earlier feedback, which substantially improved the manuscript. No further changes were requested.

**Reviewer #3**

We thank Reviewer #3 for these detailed comments. We address each point below.

**Comment 1.**

*Concerns regarding edited plants and editing efficiency. Only 5 independent lines were obtained for both PE6d and PEmax, representing an insufficient sample size that precludes a robust and reliable comparison of editing efficiency between the two systems.*

**Response:**

We thank the reviewer for this important comment. We agree that the number of regenerated plants evaluated at each target site was insufficient for a statistically robust comparison of the intrinsic editing efficiencies of PE6c, PE6d, and PEmax. We have therefore revised the Results section and removed the statement that PE6c “consistently outperformed” the other editors. **The revised text reports** the observed outcomes without presenting them as a statistically supported **comparison of editing efficiencies.**

We would also like to clarify the experimental design. During the initial screening, all three prime editors were evaluated at five target sites (Chr1-2, Chr3, Chr7, Chr11, and *OsMOC1*), with five regenerated plants analyzed per editor at each site, corresponding to 25 plants per editor across the five sites. This initial screen was intended to identify an editor suitable for installing the *attP* landing pad, rather than to provide a statistically powered comparison of editing efficiencies. Under our experimental conditions, PE6c yielded regenerated plants carrying correctly installed *attP* alleles across multiple loci, whereas precise installation was detected in only one plant generated using PE6d and was not detected among the plants analyzed using PEmax. Based on these observed outcomes, PE6c was therefore selected for further experiments. Additional regenerated plants were subsequently screened using PE6c to recover precise attP insertions, including homozygous lines suitable for the downstream experiments.

The conclusions of the study do not depend on **establishing an efficiency** ranking among the three prime editors. **PE6c, the editor used throughout the remainder of the study, was evaluated in additional regenerated plants** across four loci (**S7** Table: n=19 at **Chr1-2**, n=16 at **Chr3**, n=33 at **Chr7**, and n=400 at **Chr11**). **S7** Table summarizes all **regenerated plants** analyzed for each editor, including **those classified as precise *attP*-positive plants classified as** homozygous, heterozygous, chimeric, or of undetermined zygosity, as well as non-precisely edited, **and wild type plants.** We have tempered every comparative statement accordingly.

The text on page 10 of the Results section has therefore been revised as follows:

“During the initial screening, five regenerated plants were analyzed for each prime editor at each of the five target sites. PE6c yielded regenerated plants carrying precisely installed *attP* alleles at multiple loci, whereas PE6d yielded one regenerated plant with a precisely installed *attP* allele, and no precise *attP* installations were detected among the regenerated plants analyzed using PEmax under our experimental conditions (Fig. 2C and S7 Table). Given the limited number of regenerated plants evaluated per editor at each target site, these observations do not support a statistically robust comparison of the relative editing efficiencies of the three prime editing systems. Based on the observed outcomes, PE6c was selected for subsequent experiments because it reproducibly supported precise *attP* installation across multiple loci. Additional regenerated plants were subsequently screened using PE6c, yielding a total of 25 plants carrying correctly installed attP alleles across four GSH loci: 12 at Chr 1-2, 10 at Chr 3, two at Chr 7, and one at Chr 11 (Fig. 2C and S7 and S9 Tables).”

**Comment 2.**

*Issue with vector sequence annotation. Based on the main text and supplementary sequence data, the authors used the evopreQ1 motif. According to established literature, an 8 bp linker is typically placed between the RTT-PBS region and evopreQ1, but this linker sequence is absent from the provided sequence information.*

**Response:**

We thank the reviewer for pointing out this ambiguity. We would like to clarify that the epegRNAs used in this study contained the trimmed evopreQ1 (tevopreQ1) motif rather than the full-length evopreQ1 motif. tevopreQ1 was **generated by removing five nucleotides from** evopreQ1 **to reduce potential misfolding while retaining its ability to enhance prime editing**.

**In the initial epegRNA designs reported by Nelson et al., evopreQ1 and other structural motifs were appended to the 3′ end of the PBS through a common 8-nt linker. However, the authors subsequently evaluated pegLIT-designed linkers and found that their inclusion with evopreQ1- or tevopreQ1-containing epegRNAs did not significantly increase editing efficiency relative to the corresponding epegRNAs without a linker.** Therefore, our **epegRNAs were designed with tevopreQ1 directly appended to the PBS**, without an intervening linker. The absence of an 8-nt linker **from** the provided sequences is **thus** intentional **and** not an annotation omission.

**To eliminate ambiguity**, we have revised the manuscript and Supplementary Information to consistently **identify the structural motif as** tevopreQ1 **and to explicitly indicate that no intervening linker was included in our epegRNA design. We also corrected the typographical error “ttreQ1” in the S4 Fig legend to “tevopreQ1.”**

**Referenced study: Nelson JW, Randolph PB, Shen SP, Everette KA, Chen PJ, Anzalone AV, et al. Engineered pegRNAs improve prime editing efficiency. *Nature Biotechnology.* 2022;40:402–410. Doi: 10.1038/s41587-021-01039-7.**

**Comment 3.**

*Issue with the gel electrophoresis image in Fig. S5A. The precisely edited sequence is 8 bp shorter than the wild-type (WT) sequence. In the right panel, the middle band corresponds to the WT control, and the right lane shows the digested product of the precisely edited allele. A faint band is visible in this lane: if it represented an incompletely digested edited product, it would be expected to migrate faster (appear smaller) than the WT band, yet it appears slightly larger than the WT band in the middle lane.*

**Response:**

We thank the reviewer for this careful observation and have revised Fig. S5A to clarify the analysis. Because the edited *attP* allele at this locus differs from the WT allele by only 8 bp (335 bp versus 343 bp), the two undigested amplicons cannot be reliably distinguished by migration on this gel. We therefore genotyped this locus by HincII digestion. The Bxb1 *attP* sequence contains a HincII recognition site, resulting in cleavage of the edited amplicon into the expected 192-bp and 143-bp fragments, whereas the WT amplicon lacks this site and remains uncut at 343 bp. These two diagnostic digestion products are indicated by red arrowheads in the revised figure.

We agree that the identity of the additional faint upper band in the digested sample cannot be conclusively determined from its apparent migration. Given its low intensity and the small 8-bp difference between the WT and edited undigested amplicons, we cannot reliably assign it as either residual WT product or an incompletely digested edited product. We have therefore avoided assigning an identity to this band and **revised the Fig. S5 legend accordingly.** Precise installation of the attP sequence was independently confirmed by Sanger sequencing (Fig. S5B).

**Because the unresolved faint band prevents definitive determination of the T_0_ event’s zygosity, we no longer classify this event as homozygous. In Fig. S5C, it is now described as a plant carrying a precise** attP **insertion, and the corresponding legend states that its zygosity could not be conclusively determined. We also revised S7 Table by adding a “Precise, zygosity undetermined” category and reclassified the PE6c-generated** OsMOC1 **event accordingly. The event remains counted as one sequence-confirmed precise** attP**-positive plant among the eight independent plants analyzed, corresponding to an installation frequency of 12.5%.**

**To maintain consistency, we revised the relevant text in the Results part of the manuscript and the description of S7 Table to include precise events of undetermined zygosity. We also revised the Methods to clarify that HincII digestion was used to detect the restriction site introduced within the** attP **sequence and thereby support the identification of** attP**-containing alleles, rather than to conclusively determine zygosity. Finally, Fig. S5D and its legend were revised to describe the T₁ progeny as derived from the** OsMOC1 attP**-positive T_0_ event, without referring to the parental event as homozygous.**

**Comment 4.**

*Issue with the gel electrophoresis image in Fig. S5D. The WT control band in the second-to-last lane is extremely faint. Under this condition, the failure to amplify the T-DNA fragment cannot be confidently interpreted as a true negative; it may instead result from poor DNA quality of the sample.*

**Response:**

We thank the reviewer for this observation. We agree that the WT control band in the *attP/OsMOC1* PCR is faint. However, the identification of T-DNA-free T_1_ plants was not based on the intensity of this separate WT control. For each T₁ plant, the same genomic DNA preparation was analyzed by both attP/OsMOC1 PCR and T-DNA-specific PCR. The plants indicated by red arrowheads showed successful amplification of the genomic attP/OsMOC1 locus, demonstrating that the corresponding DNA samples were suitable for PCR, whereas no T-DNA-specific amplicon was detected. Therefore, the absence of T-DNA amplification in these samples is unlikely to have resulted from poor DNA quality. To make this interpretation explicit, we revised the legend of Fig. S5D to clarify the criteria used to identify T-DNA-free plants.

**Comment 5.**

*Although the study presents phenotypic data for plants with large fragment insertions, only seedling-stage phenotypes are shown (Fig. S16). These data do not demonstrate the functional effect or biological consequence of the inserted fragment.*

**Response:**

We thank the reviewer for this important comment and agree that the seedling-stage images presented in Fig. S16 do not demonstrate the functional activity or biological consequence of the integrated carotenoid cassette. **We did not intend to make such a claim.** The primary objective of this study was to demonstrate the technical feasibility of the PrimeStack platform for the precise, site-specific integration of large DNA sequences at predefined genomic loci in rice. The 5.3-kb carotenoid biosynthesis cassette was selected as a proof-of-concept multikilobase cargo, and its targeted integration at the Chr 1-2 genomic safe harbor was supported by amplification of both recombination junctions and Sanger sequencing of the corresponding junction products.

**The limited purpose of S16 Fig. is to document that regenerated plants carrying the targeted 5.3-kb integration showed no obvious morphological differences relative to WT plants at five weeks after transfer to soil under the evaluated growth conditions.** Because the carotenoid cassette is driven by endosperm-specific promoters, these vegetative-stage images were not intended to assess its functional activity. We have revised the Results section where S16 Fig. is cited to clarify this limited purpose.

We also revised the Results to state explicitly that seeds obtained from these T_0_ plants did not display an obvious visible phenotype associated with carotenoid **accumulation.** Because transgene expression and carotenoid accumulation were not **further evaluated**, we draw no conclusions regarding the functional activity **of** the integrated carotenoid cassette. Although expression of the cassette could potentially be influenced by genomic, epigenetic, or physiological factors, these possibilities were not investigated in the present study, and we therefore do not attribute the absence of a visible carotenoid phenotype to any specific mechanism.

Finally, we added this limitation to the Discussion and identified molecular and biochemical characterization of cassette expression and carotenoid accumulation as objectives for future work. The conclusions of the present study are therefore restricted to the targeted integration of the 5.3-kb cassette, as supported by junction-specific PCR and Sanger sequencing, and do not include claims regarding its functional output.

**Comment 6.**

*Sample sizes are excessively small for many transformation events in the study. For instance, only 5 independent lines were analyzed for attP knock-in mediated by PE6d and PEmax, respectively. Similarly, only 10 calli per group were examined in the 9.4 kb large fragment insertion assay for eeBxb1, evoBxb1 and WT Bxb1.*

**Response:**

We thank the reviewer for pointing out this important limitation. We agree that the relatively small sample sizes in some of the transformation experiments limit the strength of quantitative comparisons among the different editing and recombination systems.

As discussed in our response to Comment 1, **for each of the five target sites (Chr1-2, Chr3, Chr7, Chr11, and *OsMOC1*), five independent regenerated plants were initially analyzed for each prime editor (PE6c, PE6d, and PEmax), resulting in a total of 25 regenerated plants analyzed per prime editor across the five target sites. Thus, the sample size of five refers to the number of independent regenerated plants analyzed per target site for each editor, rather than the total number of plants analyzed per editor. Nevertheless, we agree that five regenerated plants per editor at an individual target site provides limited statistical power for locus-specific quantitative comparisons of editing efficiency.** We have therefore revised the Results section to avoid presenting these data as a statistically robust comparison of prime editing efficiencies and instead report the observed editing outcomes descriptively. Based on these outcomes, PE6c was selected for the subsequent *attP* landing-pad installation experiments.

We also acknowledge that the 9.4-kb integration experiment was performed using 10 pooled callus samples per recombinase group, with each pooled sample consisting of 10 callus pieces, for eeBxb1, evoBxb1, and WT Bxb1. This experiment was intended as an initial proof-of-concept assessment of whether the larger 9.4-kb cargo could be integrated at the pre-installed *attP* landing site, rather than as a comprehensive quantitative comparison of recombination efficiencies among the three Bxb1 variants. Therefore, the proportion of pooled samples positive for targeted integration in this experiment should be interpreted descriptively and not as evidence of statistically significant differences among eeBxb1, evoBxb1, and WT Bxb1. To more accurately describe the experimental design and avoid overinterpretation of these data, we revised the corresponding Results section to specify the pooling strategy and sample size and to clarify that the 40-50% values represent the proportion of pooled callus samples in which targeted integration was detected, rather than a statistically robust comparison of recombination efficiencies among the three Bxb1 variants.

**Additional editorial and policy items**

In addition to addressing the reviewer’s comments, we made the following changes in response to the editorial and technical requests.

**Literature review.** We reviewed the literature published since the original submission and did not identify additional directly relevant studies requiring citation.

**Data availability.** In accordance with PLOS’ Data Policy, the numerical source data underlying the figures, including the positive counts and total sample numbers used to calculate the reported percentages, are provided in the Supporting Information tables.

**Blot and gel data.** In accordance with the journal’s blot-and-gel reporting requirements, we prepared the original, uncropped, and minimally adjusted images supporting all gel-based panels in the main and Supporting Information figures. These images are provided as a separate Supporting Information file and are clearly labeled and mapped to their corresponding figure panels.

**BioRender citations and licenses.** Following the technical request, Figs. 1, 3, S12, and S17 were updated in BioRender under Edith Sanchez Hernandez’s account, and a separate open-access publication license was generated for each completed figure. The corresponding figure legends were revised to include the figure-specific citations provided in the new licenses, as listed below. Copies of all four publication licenses are provided with the revision.

- Fig. 1: Created in BioRender. Sanchez hernandez, E. (2026) [https://BioRender.com/uwdtl51](https://biorender.com/uwdtl51)
- Fig. 3: Created in BioRender. Sanchez hernandez, E. (2026) [https://BioRender.com/xi0j927](https://biorender.com/xi0j927)
- Fig. S12: Created in BioRender. Sanchez hernandez, E. (2026) [https://BioRender.com/8knw1ak](https://biorender.com/8knw1ak)
- Fig. S17: Created in BioRender. Sanchez hernandez, E. (2026) [https://BioRender.com/kwbygr8](https://biorender.com/kwbygr8)

**Copyright permission for Fig. S16.** The photographs presented in Fig. S16 were taken by co-author Haroon Butt. The photographer and all authors authorize their publication under the Creative Commons Attribution 4.0 International (CC BY 4.0) License. This confirmation was also provided directly in response to the technical-request email.

We are grateful to the reviewers and editors for their careful and constructive evaluation. We believe that the revisions have clarified the molecular evidence, appropriately aligned the conclusions with the supporting data, and strengthened the manuscript. We hope that the revised manuscript is now suitable for publication in *PLOS Biology*, and we would be happy to provide any additional information the editors or reviewers may require.

On behalf of all authors,

Magdy M. Mahfouz

Corresponding author

[magdy.mahfouz@kaust.edu.sa](mailto:magdy.mahfouz@kaust.edu.sa)
