# Supplementary material for: PrimeStack editors enable efficient and site-specific integration of large multigene cargos in plants
Source: PLoS Biol. 2026 Oct 13;24(10):e3003778. doi: 10.1371/journal.pbio.3003778 (PMC13693989; doi:10.1371/journal.pbio.3003778)
Supplement: Peer review history

# Editor report: Decision Letter 0

**DOI:** 10.1371/journal.pbio.3003778.r001

**Authors:** Richard Hodge

10 Apr 2026

Dear Dr Mahfouz,

Thank you for submitting your manuscript entitled "High-efficiency, site-specific integration of kilobase-scale DNA into plant genomic safe harbors via PrimeStack editors" for consideration as a Research Article by PLOS Biology. Please accept my sincere apologies for the delay in getting back to you with feedback.

Your manuscript has now been evaluated by the PLOS Biology editorial staff and I am writing to let you know that we would like to send your submission out for external peer review.

However, before we can send your manuscript to reviewers, we need you to complete your submission by providing the metadata that is required for full assessment. To this end, please login to Editorial Manager where you will find the paper in the 'Submissions Needing Revisions' folder on your homepage. Please click 'Revise Submission' from the Action Links and complete all additional questions in the submission questionnaire.

Once your full submission is complete, your paper will undergo a series of checks in preparation for peer review. After your manuscript has passed the checks it will be sent out for review. To provide the metadata for your submission, please Login to Editorial Manager (https://www.editorialmanager.com/pbiology) within two working days, i.e. by Apr 12 2026 11:59PM.

If your manuscript has been previously peer-reviewed at another journal, PLOS Biology is willing to work with those reviews in order to avoid re-starting the process. Submission of the previous reviews is entirely optional and our ability to use them effectively will depend on the willingness of the previous journal to confirm the content of the reports and share the reviewer identities. Please note that we reserve the right to invite additional reviewers if we consider that additional/independent reviewers are needed, although we aim to avoid this as far as possible. In our experience, working with previous reviews does save time.

If you would like us to consider previous reviewer reports, please edit your cover letter to let us know and include the name of the journal where the work was previously considered and the manuscript ID it was given. In addition, please upload a response to the reviews as a 'Prior Peer Review' file type, which should include the reports in full and a point-by-point reply detailing how you have or plan to address the reviewers' concerns.

During the process of completing your manuscript submission, you will be invited to opt-in to posting your pre-review manuscript as a bioRxiv preprint. Visit http://journals.plos.org/plosbiology/s/preprints for full details. If you consent to posting your current manuscript as a preprint, please upload a single Preprint PDF.

Feel free to email us at plosbiology@plos.org if you have any queries relating to your submission.

Kind regards,

Richard

Richard Hodge, PhD

Senior Editor, PLOS Biology

rhodge@plos.org

PLOS

Empowering researchers to transform science

Carlyle House, Carlyle Road, Cambridge, CB4 3DN, United Kingdom

California (U.S.) corporation #C2354500, based in San Francisco

# Peer review: Decision Letter 1

**DOI:** 10.1371/journal.pbio.3003778.r002

**Authors:** Richard Hodge

12 May 2026

Dear Dr Mahfouz,

Thank you for your patience while your manuscript "High-efficiency, site-specific integration of kilobase-scale DNA into plant genomic safe harbors via PrimeStack editors" was peer-reviewed at PLOS Biology. It has now been evaluated by the PLOS Biology editors, an Academic Editor with relevant expertise, and by several independent reviewers.

In light of the reviews, which you will find at the end of this email, we would like to invite you to revise the work to thoroughly address the reviewers' reports.

As you will see, the reviewers expressed interest in the reported method, but raised several important concerns. The major points related to the comprehensiveness of the performance evaluation and the insertion validation experiments, as well as aspects of the study in need of clarification. In your revision, you should thoroughly address the reviewers' concerns.

Given the extent of revision needed, we cannot make a decision about publication until we have seen the revised manuscript and your response to the reviewers' comments. Your revised manuscript is likely to be sent for further evaluation by all or a subset of the reviewers.

In addition to these revisions, you will need to complete some formatting changes, which you will receive in a follow up email. A member of our team will be in touch with a set of requests shortly.

We expect to receive your revised manuscript within 3 months. Please email us (plosbiology@plos.org) if you have any questions or concerns, or would like to request an extension.

At this stage, your manuscript remains formally under active consideration at our journal; please notify us by email if you do not intend to submit a revision so that we may withdraw it.

**IMPORTANT - SUBMITTING YOUR REVISION**

Your revisions should address the specific points made by each reviewer. Please submit the following files along with your revised manuscript:

1. A 'Response to Reviewers' file - this should detail your responses to the editorial requests, present a point-by-point response to all of the reviewers' comments, and indicate the changes made to the manuscript.

*NOTE: In your point-by-point response to the reviewers, please provide the full context of each review. Do not selectively quote paragraphs or sentences to reply to. The entire set of reviewer comments should be present in full and each specific point should be responded to individually, point by point.

You should also cite any additional relevant literature that has been published since the original submission and mention any additional citations in your response.

2. In addition to a clean copy of the manuscript, please also upload a 'track-changes' version of your manuscript that specifies the edits made. This should be uploaded as a "Revised Article with Changes Highlighted" file type.

*Re-submission Checklist*

When you are ready to resubmit your revised manuscript, please refer to this re-submission checklist: https://plos.io/Biology_Checklist

To submit a revised version of your manuscript, please go to https://www.editorialmanager.com/pbiology/ and log in as an Author. Click the link labelled 'Submissions Needing Revision' where you will find your submission record.

Please make sure to read the following important policies and guidelines while preparing your revision:

*Published Peer Review*

Please note while forming your response, if your article is accepted, you may have the opportunity to make the peer review history publicly available. The record will include editor decision letters (with reviews) and your responses to reviewer comments. If eligible, we will contact you to opt in or out. Please see here for more details:

https://blogs.plos.org/plos/2019/05/plos-journals-now-open-for-published-peer-review/

*PLOS Data Policy*

Please note that as a condition of publication PLOS' data policy (http://journals.plos.org/plosbiology/s/data-availability) requires that you make available all data used to draw the conclusions arrived at in your manuscript. If you have not already done so, you must include any data used in your manuscript either in appropriate repositories, within the body of the manuscript, or as supporting information (N.B. this includes any numerical values that were used to generate graphs, histograms etc.). For an example see here: http://www.plosbiology.org/article/info%3Adoi%2F10.1371%2Fjournal.pbio.1001908#s5

*Blot and Gel Data Policy*

We require the original, uncropped and minimally adjusted images supporting all blot and gel results reported in an article's figures or Supporting Information files. We will require these files before a manuscript can be accepted so please prepare them now, if you have not already uploaded them. Please carefully read our guidelines for how to prepare and upload this data: https://journals.plos.org/plosbiology/s/figures#loc-blot-and-gel-reporting-requirements

*Protocols deposition*

To enhance the reproducibility of your results, we recommend that if applicable you deposit your laboratory protocols in protocols.io, where a protocol can be assigned its own identifier (DOI) such that it can be cited independently in the future. Additionally, PLOS ONE offers an option for publishing peer-reviewed Lab Protocol articles, which describe protocols hosted on protocols.io. Read more information on sharing protocols at https://plos.org/protocols?utm_medium=editorial-email&utm_source=authorletters&utm_campaign=protocols

Thank you again for your submission to our journal. We hope that our editorial process has been constructive thus far, and we welcome your feedback at any time. Please don't hesitate to contact us if you have any questions or comments.

Sincerely,

Taylor Hart, PhD -- on behalf of the main handling editor, Richard Hodge, who is out of the office

Associate Editor, PLOS Biology

thart@plos.org

Richard

Richard Hodge, PhD

Senior Editor

PLOS Biology

rhodge@plos.org

------------------------------------

REVIEWS:

Reviewer #1: Manuscript Number: PBIOLOGY-D-26-01007R1

Title: High-efficiency, site-specific integration of kilobase-scale DNA into plant genomic safe harbors via PrimeStack editors

Precise, site-specific integration of large DNA sequences into plant genomes is a cornerstone of crop biotechnology and synthetic biology, yet remains constrained by random insertion, inefficient homologous recombination, and gene targeting. Here, the authors present PrimeStack, a DSB-independent platform that integrates prime editing with the unidirectional large serine integrase Bxb1, to achieve the programmable insertion of multigene, multikilobase cargos at predefined genomic safe harbors in rice. The author used the previously optimized prime editors first install attP landing sites with high fidelity and heritability followed by Bxb1-mediated recombination that generates irreversible integration of genetic information. PrimeStack achieves integration frequencies of approximately 43-46% (as detected by junction-specific PCR in rice calli). This study establishes a site-specific gene-stacking platform that enables predictable pathway construction at genomic safe harbors, thereby accelerating the development of improved and resilient crop varieties. However, the authors only provide the preliminary results with some claims either in the Abstract or in the text are not supported by the data. I have the following major and minor concerns to be addressed before consideration for acceptance of this manuscript for publication.

Major Concerns

1.Page 10, the last paragraph and Page 11, the first paragraph, the authors should indicate how many regenerated lines are independent lines, and among these independent lines, how many lines are precisely edited (heterozygous and/or homozygous), how many lines are non-precisely edited, how many are WT lines, respectively. I would like to suggest the authors add a Table in the text or in Supplementary Data. In this way, you can evaluate the performances of three prime editor variants in rice stable lines. To be honest, I don't think employing amplicon NGS could provide statistical comparison of the performances of these three prime editor variants.

2.Page 13, I am wondering if the authors only tested the performance of PrimeStack in calli with pre-installed attP site? To be informative to the readers, the authors need to provide the information in the text on how many independent lines carrying the precisely integrated carotenoid synthesis cassettes, the ratios of heterozygous and/or homozygous lines, has been obtained? This information would benefit the readers to get an initial view on the efficiency of the PrimeStack platform established in this study. Furthermore, are these precisely edited lines with carotenoid synthesis cassettes morphologically normal in comparison to the WT control? In addition, it would be also interesting to investigate whether the carotenoid content has been increased in the rice brown grains and polished grains.

3.Although this study establishes a platform, PrimeStack, which enables site-specific integration of kilobase-scale DNA into plant genomic safe harbors, benefiting the integration of foreign genes/cassettes at these safe harbors efficiently. The presences of pre-installed recognition sites attP landing sites inside the agriculturally important endogenous beneficial genes/alleles loci still raise biosafety concerns. I would suggest the authors present a few sentences to address the limitation of this study in the Discussion section.

4.Page 14, the last sentence in the first paragraph, the authors mentioned that 'Although regeneration of plants carrying the dual trait donor was not achieved under the tested selection conditions, the robust molecular validation at the callus stage establishes successful site-specific stacking of multiple traits at a single genomic locus'. The novelty of this study is somehow compromised without the obtainment of regenerated lines with precisely integrated dual gene cassettes. Are there any reasons underlying this phenomenon?

5.Page 15 and 16, in the section 'Site-specific integration efficiencies using engineered Bxb1 variants'. As I mentioned previously, the authors need to add a Table either in the text or in Supplementary Data to provide the detailed information on independent lines obtained, of which, the number of precisely edited lines, heterozygous or homozygous, in order to evaluate the relative efficiency of each Bxb1 variants. Besides, to provide the proof of the concept that the integration of foreign gene/cassette at the identified safe harbor through PrimeStack indeed has no side-effects on agronomic traits and its utility in crop improvement, I am wondering whether these precisely edited lines with carotenoid synthesis cassettes morphologically normal in comparison to the WT control? In addition, it would be also interesting to investigate whether the carotenoid content has been increased in the rice brown grains and polished grains.

Minor concerns

1.In the Abstract section, several phrases need to be revised. For example, 'comparing favorably with bidirectional Cre-lox systems' is there any data to support this claim?

2.Remove all the Figure legends to the end of text.

3.The authors used biolistic co-delivery of a carotenoid minicircle donor and Bxb1 recombinase into attP engineered callus. I am wondering why the authors used biolistic delivery but not the Agrobacterium-mediated transformation. Is the biolistic delivery more efficient than Agrobacterium-mediated transformation in this case?

4.Page 7, the authors mentioned 'The final panel comprised two targets within a genomic safe harbor shared across 33 rice genomes on chromosome 1'. Here, it is not clear about the meaning of 'across 33 rice genomes'.

Reviewer #2: The authors showcase a novel recombinase-integration system for plant genome editing using the Bxb1 system, with prior prime-editing mediated recognitions site installation in genomic safe harbors. Compared to a similar system using Cre in plants, this work provides a directional and irreversible recombination system, which can have valuable applications in the future, though the present data shows a low level of recombination efficiency.

Major Comments

1.The abstract sentence on efficiency can be interpreted to mean 43-46% of regenerated lines have a stably heritable allele of the targeted large insertion. It is not as clear as in the body text in discussing that this is the % of calli with any detectable large insertion. Otherwise, the findings are suitable to support the assertions of efficient recombination installation by PE6c and a qualitative achievement of large-cargo Bxb1 insertion.

Minor Comments

1.Abstract text, line 1, "site-specific integration of large DNA sequences…" cannot be a "cornerstone" if it is hard to achieve. The idiomatic meaning of cornerstone implies it is a foundational aspect. Consider rewording "cornerstone" to "long-standing goal" or something else.

2.Please ensure consistency and accurate notation of genes in italics and proteins not in italics. Please ensure this for the figures as well. Example: figure 3 legend, "attB" not italicized.

3.Please ensure high resolution figures for the final submission.

Reviewer #3: This manuscript by Sánchez et al. reported the development of PrimeStack, a DSB-free genome engineering platform that combines prime editing (PE) with Bxb1 integrase and its evolved variants. This system enables the programmable and irreversible insertion of multi-gene cassettes into experimentally validated genomic safe harbors (GSHs) in rice. Nevertheless, multiple deficiencies need to be addressed and revised prior to acceptance for publication.

1.The authors designed eight GSH loci and two sites adjacent to translational regions as attP target sites, and acquired transgenic materials with attP insertion at four GSH loci and the MOC1-proximal site. However, only the Chr1-2 line was selected for large-fragment insertion testing, and the attP-inserted line near the MOC1 locus was not included as a control. With only one single locus (Chr1-2) and two insertion events (5.3 Kb and 9.4 Kb) characterized, it remains insufficient to fully validate the reliability and stability of this large DNA fragment editing system.

2.Three PE systems (PEmax, PE6c and PE6d) were constructed for attP insertion, and three recombinases (eeBxb1, evoBxb1 and wild-type Bxb1) were adopted in parallel. Notably, Sanger sequencing was exclusively used for post-transformation comparison of different systems, which only supports qualitative analysis. The rationale for not adopting NGS for quantitative analysis is unclear, especially considering that eeBxb1, evoBxb1 and Bxb1 exhibited highly similar performance in 5.3 Kb large-fragment insertion.

3.The authors have obtained transgenic lines with 5.3 kb fragment insertion and confirmed the insertion accuracy by sequencing, but no phenotypic data have been provided to verify and demonstrate the functional effects of exogenous gene insertion.

4.Three different recombinases were also applied for 9.4 Kb large-fragment insertion, and successful fragment integration was detected at the rice callus stage. However, comparative data among the three recombinases are missing. The performance and functional differences of eeBxb1, evoBxb1 and Bxb1 in facilitating 9.4 Kb large-fragment insertion should be supplemented and clarified.

# Author comment: Author response to Decision Letter 2

**DOI:** 10.1371/journal.pbio.3003778.r003

9 Jun 2026

Attachment Submitted filename: 0601-26 Rebuttal letter revised.docx

# Peer review: Decision Letter 2

**DOI:** 10.1371/journal.pbio.3003778.r004

**Authors:** Richard Hodge

18 Jul 2026

Dear Dr Mahfouz,

Thank you for your patience while we considered your revised manuscript "High-efficiency, site-specific integration of kilobase-scale DNA into plant genomic safe harbors via PrimeStack editors" for publication as a Research Article at PLOS Biology. Please accept my sincere apologies for the delays that you have experienced during this round of the peer review process. Your revised study has been evaluated by the PLOS Biology editors, the Academic Editor and two the original reviewers.

In light of the reviews, which you will find at the end of this email, we would like to invite you to revise the work to thoroughly address the reviewers' reports.

As you will see, while Reviewer #2 is now satisfied with the revised version, Reviewer #3 continues to raise some important concerns. This includes the small sample sizes to draw conclusions about editing efficiency between the PE6d and PEmax prime editing systems and the transformation events, so the reviewer notes that this should be increased. In addition, the reviewer raises several concerns with the gel electrophoresis images in Figure S5 and notes that the additional phenotypic data does not demonstrate a functional effect of the inserted fragment.

Given the extent of revision needed, we cannot make a decision about publication until we have seen the revised manuscript and your response to the reviewers' comments. Your revised manuscript is likely to be sent for further evaluation by all or a subset of the reviewers.

In addition to these revisions, you will need to complete some formatting changes, which you will receive in a follow up email. A member of our team will be in touch with a set of requests shortly.

We expect to receive your revised manuscript within 3 months. Please email us (plosbiology@plos.org) if you have any questions or concerns, or would like to request an extension.

At this stage, your manuscript remains formally under active consideration at our journal; please notify us by email if you do not intend to submit a revision so that we may withdraw it.

**IMPORTANT - SUBMITTING YOUR REVISION**

Your revisions should address the specific points made by each reviewer. Please submit the following files along with your revised manuscript:

1. A 'Response to Reviewers' file - this should detail your responses to the editorial requests, present a point-by-point response to all of the reviewers' comments, and indicate the changes made to the manuscript.

*NOTE: In your point-by-point response to the reviewers, please provide the full context of each review. Do not selectively quote paragraphs or sentences to reply to. The entire set of reviewer comments should be present in full and each specific point should be responded to individually, point by point.

You should also cite any additional relevant literature that has been published since the original submission and mention any additional citations in your response.

2. In addition to a clean copy of the manuscript, please also upload a 'track-changes' version of your manuscript that specifies the edits made. This should be uploaded as a "Revised Article with Changes Highlighted" file type.

*Re-submission Checklist*

When you are ready to resubmit your revised manuscript, please refer to this re-submission checklist: https://plos.io/Biology_Checklist

To submit a revised version of your manuscript, please go to https://www.editorialmanager.com/pbiology/ and log in as an Author. Click the link labelled 'Submissions Needing Revision' where you will find your submission record.

Please make sure to read the following important policies and guidelines while preparing your revision:

*Published Peer Review*

Please note while forming your response, if your article is accepted, you may have the opportunity to make the peer review history publicly available. The record will include editor decision letters (with reviews) and your responses to reviewer comments. If eligible, we will contact you to opt in or out. Please see here for more details:

https://blogs.plos.org/plos/2019/05/plos-journals-now-open-for-published-peer-review/

*PLOS Data Policy*

Please note that as a condition of publication PLOS' data policy (http://journals.plos.org/plosbiology/s/data-availability) requires that you make available all data used to draw the conclusions arrived at in your manuscript. If you have not already done so, you must include any data used in your manuscript either in appropriate repositories, within the body of the manuscript, or as supporting information (N.B. this includes any numerical values that were used to generate graphs, histograms etc.). For an example see here: http://www.plosbiology.org/article/info%3Adoi%2F10.1371%2Fjournal.pbio.1001908#s5

*Blot and Gel Data Policy*

We require the original, uncropped and minimally adjusted images supporting all blot and gel results reported in an article's figures or Supporting Information files. We will require these files before a manuscript can be accepted so please prepare them now, if you have not already uploaded them. Please carefully read our guidelines for how to prepare and upload this data: https://journals.plos.org/plosbiology/s/figures#loc-blot-and-gel-reporting-requirements

*Protocols deposition*

To enhance the reproducibility of your results, we recommend that if applicable you deposit your laboratory protocols in protocols.io, where a protocol can be assigned its own identifier (DOI) such that it can be cited independently in the future. Additionally, PLOS ONE offers an option for publishing peer-reviewed Lab Protocol articles, which describe protocols hosted on protocols.io. Read more information on sharing protocols at https://plos.org/protocols?utm_medium=editorial-email&utm_source=authorletters&utm_campaign=protocols

Thank you again for your submission to our journal. We hope that our editorial process has been constructive thus far, and we welcome your feedback at any time. Please don't hesitate to contact us if you have any questions or comments.

Best regards,

Richard

Richard Hodge, PhD

Senior Editor, PLOS Biology

rhodge@plos.org

------------------------------------

REVIEWS:

Reviewer #2: The authors have addressed all my points. I have no more concerns.

Reviewer #3: 1.Concerns regarding edited plants and editing efficiency. Only 5 independent lines were obtained for both PE6d and PEmax, representing an insufficient sample size that precludes a robust and reliable comparison of editing efficiency between the two systems.

2.Issue with vector sequence annotation. Based on the main text and supplementary sequence data, the authors used the evopreQ1 motif. According to established literature, an 8 bp linker is typically placed between the RTT-PBS region and evopreQ1, but this linker sequence is absent from the provided sequence information.

3.Issue with the gel electrophoresis image in Fig. S5A. The precisely edited sequence is 8 bp shorter than the wild‑type (WT) sequence. In the right panel, the middle band corresponds to the WT control, and the right lane shows the digested product of the precisely edited allele. A faint band is visible in this lane: if it represented an incompletely digested edited product, it would be expected to migrate faster (appear smaller) than the WT band, yet it appears slightly larger than the WT band in the middle lane.

4.Issue with the gel electrophoresis image in Fig. S5D. The WT control band in the second‑to‑last lane is extremely faint. Under this condition, the failure to amplify the T‑DNA fragment cannot be confidently interpreted as a true negative; it may instead result from poor DNA quality of the sample.

5.Although the study presents phenotypic data for plants with large fragment insertions, only seedling‑stage phenotypes are shown (Fig. S16). These data do not demonstrate the functional effect or biological consequence of the inserted fragment.

6.Sample sizes are excessively small for many transformation events in the study. For instance, only 5 independent lines were analyzed for attP knock‑in mediated by PE6d and PEmax, respectively. Similarly, only 10 calli per group were examined in the 9.4 kb large fragment insertion assay for eeBxb1, evoBxb1 and WT Bxb1.

# Author comment: Author response to Decision Letter 3

**DOI:** 10.1371/journal.pbio.3003778.r005

1 Sep 2026

Attachment Submitted filename: 0829-26 REBUTTAL LETTER C. REVISED.docx

# Editor report: Decision Letter 3

**DOI:** 10.1371/journal.pbio.3003778.r006

**Authors:** Richard Hodge

4 Sep 2026

Dear Dr Mahfouz,

Thank you for your patience while we considered your revised manuscript "High-efficiency, site-specific integration of kilobase-scale DNA into plant genomic safe harbors via PrimeStack editors" for publication as a Research Article at PLOS Biology. This revised version of your manuscript has been evaluated by the PLOS Biology editors and the Academic Editor.

Based on our Academic Editor's assessment of your revision, I am pleased to say that we are likely to accept this manuscript for publication, provided you satisfactorily address the following data and other policy-related requests that I have provided below (A-H):

(A) We routinely suggest changes to titles to ensure maximum accessibility for a broad, non-specialist readership. In this case, we would suggest the following edit to the title. Please ensure you change both the manuscript file and the online submission system, as they need to match for final acceptance:

“PrimeStack editors enable efficient and site-specific integration of large multigene cargos in plants”

(B) After discussion within the editorial team, we think that your manuscript would be a better fit as a 'Methods and Resources Article' at the journal. I have therefore changed the article type during this round of revision, but please do let me know if you have any questions or concerns about this.

(C) You may be aware of the PLOS Data Policy, which requires that all data be made available without restriction: http://journals.plos.org/plosbiology/s/data-availability. For more information, please also see this editorial: http://dx.doi.org/10.1371/journal.pbio.1001797

Note that we do not require all raw data. Rather, we ask that all individual quantitative observations that underlie the data summarized in the figures and results of your paper be made available in one of the following forms:

-Supplementary files (e.g., excel). Please ensure that all data files are uploaded as 'Supporting Information' and are invariably referred to (in the manuscript, figure legends, and the Description field when uploading your files) using the following format verbatim: S1 Data, S2 Data, etc. Multiple panels of a single or even several figures can be included as multiple sheets in one excel file that is saved using exactly the following convention: S1_Data.xlsx (using an underscore).

-Deposition in a publicly available repository. Please also provide the accession code or a reviewer link so that we may view your data before publication.

Regardless of the method selected, please ensure that you provide the individual numerical values that underlie the summary data displayed in the following figure panels as they are essential for readers to assess your analysis and to reproduce it:

Figure 2C-E, 5F

NOTE: the numerical data provided should include all replicates AND the way in which the plotted mean and errors were derived (it should not present only the mean/average values).

(D) Please also ensure that each of the relevant figure legends in your manuscript include information on *WHERE THE UNDERLYING DATA CAN BE FOUND*, and ensure your supplemental data file/s has a legend.

(E) Please deposit the Sanger sequencing data in a public repository, such as the GEO. We ask that you please make the sequencing data publicly available at this stage before publication.

(F) Please ensure that your Data Statement in the submission system accurately describes where your data can be found and is in final format, as it will be published as written there.

(G) Per journal policy, if you have generated any custom code during the course of this investigation, please make it available without restrictions. Please ensure that the code is sufficiently well documented and reusable, and that your Data Statement in the Editorial Manager submission system accurately describes where your code can be found. More information on our Code Policy, what and how to share can be found here: https://journals.plos.org/plosbiology/s/code-availability

Please note that we cannot accept sole deposition of code in GitHub, as this could be changed after publication. However, you can archive this version of your publicly available GitHub code to Zenodo. Once you do this, it will generate a DOI number, which you will need to provide in the Data Accessibility Statement (you are welcome to also provide the GitHub access information). See the process for doing this here: https://docs.github.com/en/repositories/archiving-a-github-repository/referencing-and-citing-content

(H) Please note that per journal policy, the species studied should be clearly stated in the abstract of your manuscript (Oryza sativa).

------------------------------------------------------------------------

As you address these items, please take this last chance to review your reference list to ensure that it is complete and correct. If you have cited papers that have been retracted, please include the rationale for doing so in the manuscript text, or remove these references and replace them with relevant current references. Any changes to the reference list should be mentioned in the cover letter that accompanies your revised manuscript.

In addition to these revisions, you may need to complete some formatting changes, which you will receive in a follow up email. A member of our team will be in touch with a set of requests shortly. If you do not receive a separate email within a few days, please assume that checks have been completed, and no additional changes are required.

We expect to receive your revised manuscript within two weeks.

To submit your revision, please go to https://www.editorialmanager.com/pbiology/ and log in as an Author. Click the link labelled 'Submissions Needing Revision' to find your submission record. Your revised submission must include the following:

- a cover letter that should detail your responses to any editorial requests, if applicable, and whether changes have been made to the reference list

- a Response to Reviewers file that provides a detailed response to the reviewers' comments (if applicable, if not applicable please do not delete your existing 'Response to Reviewers' file.)

- a track-changes file indicating any changes that you have made to the manuscript.

NOTE: If Supporting Information files are included with your article, note that these are not copyedited and will be published as they are submitted. Please ensure that these files are legible and of high quality (at least 300 dpi) in an easily accessible file format. For this reason, please be aware that any references listed in an SI file will not be indexed. For more information, see our Supporting Information guidelines:

https://journals.plos.org/plosbiology/s/supporting-information

*Published Peer Review History*

Please note that you may have the opportunity to make the peer review history publicly available. The record will include editor decision letters (with reviews) and your responses to reviewer comments. If eligible, we will contact you to opt in or out. Please see here for more details:

https://plos.org/published-peer-review-history/

*Press*

Should you, your institution's press office or the journal office choose to press release your paper, please ensure you have opted out of Early Article Posting on the submission form. We ask that you notify us as soon as possible if you or your institution is planning to press release the article.

*Protocols deposition*

To enhance the reproducibility of your results, we recommend that if applicable you deposit your laboratory protocols in protocols.io, where a protocol can be assigned its own identifier (DOI) such that it can be cited independently in the future. Additionally, PLOS ONE offers an option for publishing peer-reviewed Lab Protocol articles, which describe protocols hosted on protocols.io. Read more information on sharing protocols at https://plos.org/protocols?utm_medium=editorial-email&utm_source=authorletters&utm_campaign=protocols

Please do not hesitate to contact me should you have any questions.

Best regards,

Richard

Richard Hodge, PhD

Senior Editor, PLOS Biology

rhodge@plos.org

PLOS

Empowering researchers to transform science

Carlyle House, Carlyle Road, Cambridge, CB4 3DN, United Kingdom

California (U.S.) corporation #C2354500, based in San Francisco

# Author comment: Author response to Decision Letter 4

**DOI:** 10.1371/journal.pbio.3003778.r007

18 Sep 2026

Attachment Submitted filename: 0829-26_REBUTTAL_LETTER_C._REVISED_auresp_4.docx

# Editor report: Decision Letter 4

**DOI:** 10.1371/journal.pbio.3003778.r008

**Authors:** Richard Hodge

21 Sep 2026

Dear Magdy,

On behalf of my colleagues and the Academic Editor, Jacob Corn, I am pleased to say that we can accept your manuscript for publication, provided you address any remaining formatting and reporting issues. These will be detailed in an email you should receive within 2-3 business days from our colleagues in the journal operations team; no action is required from you until then. Please note that we will not be able to formally accept your manuscript and schedule it for publication until you have completed any requested changes.

Please take a minute to log into Editorial Manager at http://www.editorialmanager.com/pbiology/, click the "Update My Information" link at the top of the page, and update your user information to ensure an efficient production process.

PRESS

We frequently collaborate with press offices. If your institution or institutions have a press office, please notify them about your upcoming paper at this point, to enable them to help maximise its impact. If the press office is planning to promote your findings, we would be grateful if they could coordinate with biologypress@plos.org. If you have previously opted in to the early version process, we ask that you notify us immediately of any press plans so that we may opt out on your behalf.

We also ask that you take this opportunity to read our Embargo Policy regarding the discussion, promotion and media coverage of work that is yet to be published by PLOS. As your manuscript is not yet published, it is bound by the conditions of our Embargo Policy. Please be aware that this policy is in place both to ensure that any press coverage of your article is fully substantiated and to provide a direct link between such coverage and the published work. For full details of our Embargo Policy, please visit http://www.plos.org/about/media-inquiries/embargo-policy/.

Thank you again for choosing PLOS Biology for publication and supporting Open Access publishing. We look forward to publishing your study.

Best wishes,

Richard

Richard Hodge, PhD

Senior Editor, PLOS Biology

rhodge@plos.org

PLOS

Empowering researchers to transform science

Carlyle House, Carlyle Road, Cambridge, CB4 3DN, United Kingdom

California (U.S.) corporation #C2354500, based in San Francisco
